# Supplementary material for: CBLN2 promoter enables genetic access to wide-field neurons of the tree shrew superior colliculus
Source: Cell Rep Methods. 2026 Mar 6;6(3):101309. doi: 10.1016/j.crmeth.2026.101309 (PMC13030960; doi:10.1016/j.crmeth.2026.101309)
Supplement: Document S1. Figures S1–S4 and Data S1–S3 [file mmc1.pdf]

Cell Reports Methods, Volume *6*

Supplemental information

CBLN2 promoter enables genetic access to wide-field neurons of the tree shrew superior colliculus

Arda Kipcak and Alev Erisir

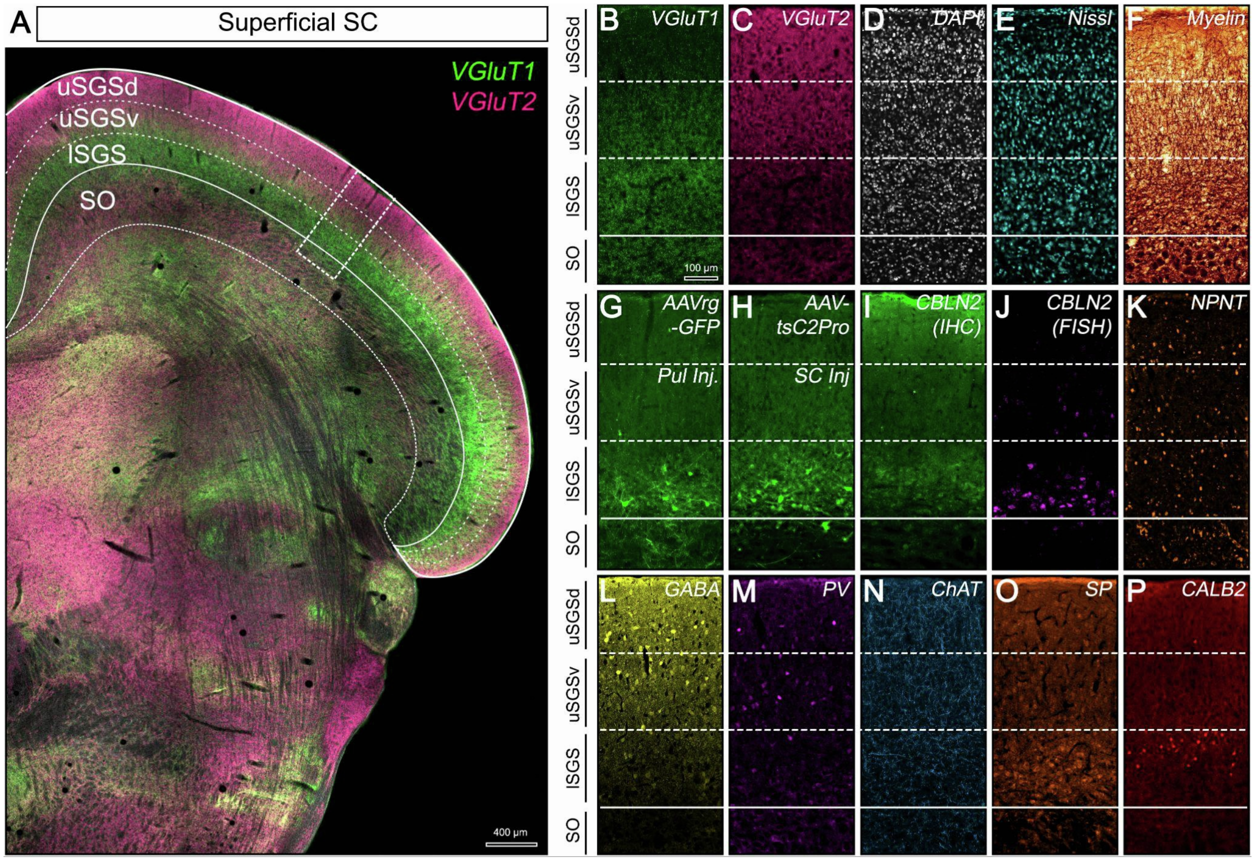

Figure S1. Expression of different markers across tree shrew superficial SC layers, related to Figure 1 and 2.

(A) Tree shrew SC coronal section highlighting superficial layers (outlined area) of SC stained with VGluT1 (Green) and VGluT2 (Purple).

(B-P) Distribution of different markers expression across the sSC. All panels are IHC staining except G: viral GFP expression; H: viral GFP expression and J: smRNA-FISH. Abbreviations (L-P): GABA: Gamma-aminobutyric acid; PV: Parvalbumin; ChAT: Choline Acetyltransferase; SP: Substance P; CALB2: Calbindin 2 (Calretinin).

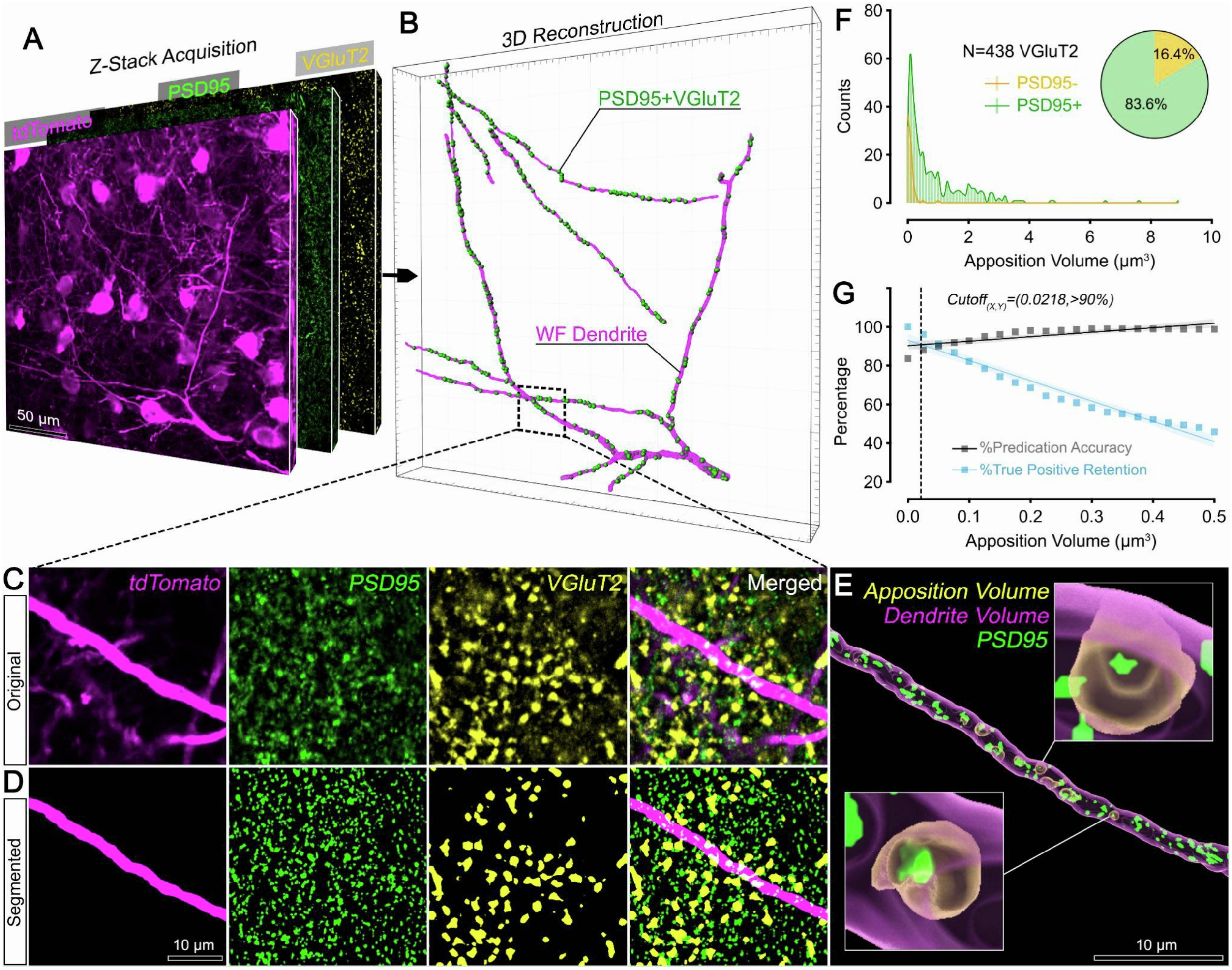

Figure S2. Experimental pipeline verification for synaptic apposition analysis, related to Figure 4.
(A) Confocal Z-Stack of viral RFP expression (AAV9-CAG-FLEX-tdTomato + AAV9-tsC2ProT-mScarlet3-Cre, magenta), IHC against PSD95 (green) and VGluT2 (yellow) to visualize WF dendrites, postsynaptic and presynaptic sites respectively.
(B) 3D reconstruction of WF dendrites (magenta), and PSD95+VGluT2 appositions (green spheres).
(C-D) Representative 2D field of view for the original image (C) and segmentation (D) of a WF dendritic branch, PSD95 and VGluT2. The merged panel at the end is used to illustrate the fidelity of the segmentation.
(E) 3D view of the same branch (magenta), showing VGluT2 appositions (yellow, i.e. not the entire VGluT2+ bouton volume), colocalizing with PSD95 (green).
(F) Distribution of PSD95+ and PSD95- VGluT2 apposition volumes, highlighting that the PSD95- appositions constitute the smallest volumes. Pie chart showing the composition of all VGluT2 appositions.
(G) X-Y plot showing the relationship between %Prediction Accuracy and %True Positive Sample Retention, as a function of (Cutoff) volumes. Solid lines with lighter colored-bands represent regressions and 95% confidence intervals respectively. Vertical dashed line shows the crossing point of the two functions (X=0.0218), that is used to filter out false-positive appositions.

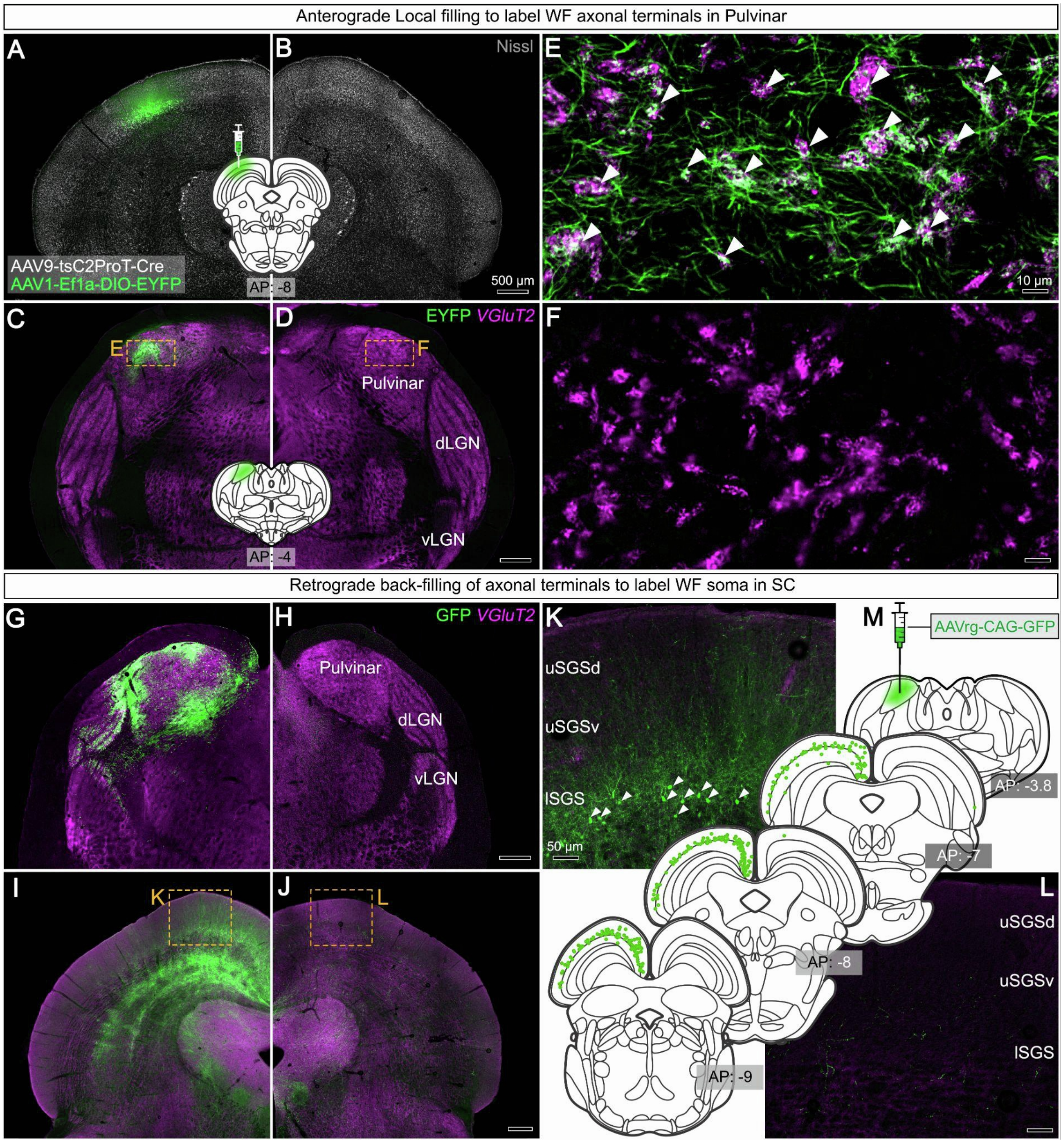

Figure S3. Tree shrew tectopulvinar projections are ipsilateral, related to Figure 3.

(A-B) Superior Colliculi showing unilateral injection of AAV9-tsC2ProT-mScarlet3-Cre + AAV1-Ef1a-DIO-EYFP in the left hemisphere. Nissl is used as counterstain.
(C-D) Thalamic sections showing EYFP-labeled axons only in the ipsilateral pulvinar. VGluT2 is used to delineate nuclei borders. dLGN: Dorsal Lateral Geniculate Nucleus; vLGN: Ventral Lateral Geniculate Nucleus.
(E-F) Higher magnification images from the ipsilateral (E) and contralateral (F) pulvinar.
(G-H) Thalamic sections showing unilateral pulvinar injection of AAVrg-CAG-GFP in the left hemisphere.
(I-J) SC sections showing retrograde GFP labeling in the ipsilateral hemisphere.
(K-L) Higher magnification images from the (K) left SC showing WF somata (white arrows) in the lower SGS and no soma labeling in the right SC (L).
(M) Schematic illustrating the pulvinar Injection and the location of the resulting retrogradely labeled WF cell bodies across the antero-posterior axis.

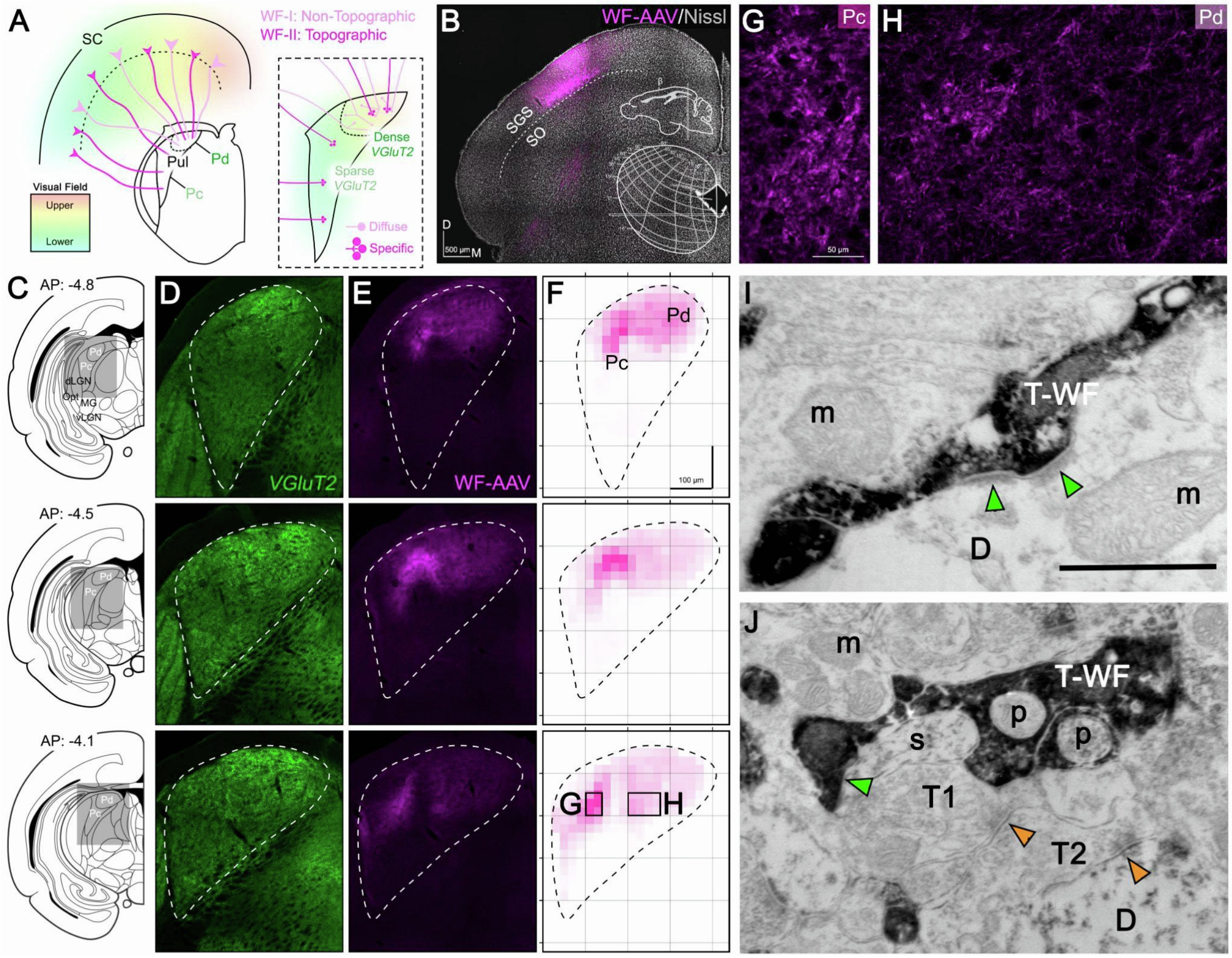

Figure S4. CBLN2 promoter-driven AAV labels two distinct tectopulvinar projection patterns, related to Figure 3.

(A) Tree shrew tectopulvinar circuit described by Luppino et al (1988)[34] and later proposed by Chomsung et al (2008)[36]. According to this model, there are two WF subtypes in the tree shrew: WF-I that non-topographically project to dorsal pulvinar (Pd) and WF-II that topographically project to the entire tectorecipient pulvinar (Pd+Pc). Visual Field topography is reflected in both structures wherein lateral to medial axis corresponds to lower to upper visual field. WF-I projections appear diffuse as unitary boutons whereas WF-II projections are specific clustered boutons. While Pc displays sparse VGluT2 originating from WF-II, Pd displays dense VGluT2 due to combined inputs from WF-I and -II.
(B) Confocal image of the SC injected with AAV9-tsC2ProT-mScarlet3-Cre+AAV1-Ef1a-DIO-EYFP (pseudocolored magenta). Inset shows the position of the section relative to parasaggittal and horizontal planes.
(C) Coronal atlas schematics showing the anatomical coordinates of imaged sections in the following panels.
(D) Pulvinar sections with VGluT2 IHC are used to help identify the borders of the pulvinar and other thalamic nuclei.
(E) Same pulvinar sections displaying the projection pattern of AAV-labeled WF axons in Pd and Pc. In all sections, fiber labeling was diffuse or diffuse+patchy in Pd and patchy in Pc.
(F) Fluorescence signal heatmaps, illustrating small intense labeling in the center and diffuse labeling in the entire Pd.
(G-H) Higher magnification image of the (G) Pc, showing clustered (specific) boutons and (H) Pd, showing both clustered (specific) boutons as well as diffusely distributed varicose fibers.
(I) Immuno-electromicrograph of AAV-labeled axon (T-WF) in Pd subdivision of pulvinar. Most axons in Pd were en passant, synapsing (green arrowheads) on large caliber dendrites (D) containing mitochondria (m). Scale bar in I=500 nm and also applies to panel J.
(J) AAV labeled axons in Pc displayed unlabeled protrusions (p), as described before by Chomsung et al (2008)[36], and formed synapses (green arrowheads) on dendrites and spines. These are often engaged in complex synaptic arrangements involving other unlabeled terminals (T1 and T2) that synapse on larger caliber dendrites (T2 ⇒D) and terminal boutons (T2⇒T1).

Data S1. CBLN2 CDS of the tree shrew, human and mouse, related to Figure 1.

Tree shrew CBLN2 CDS

```
>TSDBTID.7682.2_cds_TSDBPID.7682.2    [gene=CBLN2]    [protein=cerebellin    2    precursor]
[protein_id=TSDBPID.7682.2] [location=chr12 11137202 ~ 11147062]
ATGCCGGCGCCCGGCCGGGGCCCCTGCCGGCCGCGGCTGACCATGCCCGGGCGCCGGGGGGCGCTGCACGAGGTGGCCGGTTGCGGCTCCTGCCTGG
GGGCGGCACTGGCCCTGCTGCTGCTGCTGCTGCCCGCCTGCTGCCCGGTGCGGGCGCAGAACGACACCGAGCCCATCGTGCTGGAGGGCAAGTGCCT
GGTGGTGTGCGACTCCAGCCCGTCGGCGGACGGCGCCGTCACCTCCTCCCTGGGCATCTCCGTGCGCTCTGGCAGCGCCAAGGTGGCCTTCTCCGCC
ACGCGGAGCACCAACCACGAACCCTCCGAGATGAGCAACCGCACCATGACCATCTACTTCGACCAGGTGTTAGTAAATATTGGCAACCATTTTGATC
TTGCTTCCAGTATATTTGTAGCACCAAGAAAAGGGATTTATAGCTTCAGCTTCCACGTGGTCAAAGTGTATAACAGACAAACCATCCAGGTCAGTTT
AATGCAGAACGGCTACCCAGTGATCTCGGCGTTTGCAGGAGACCAGGACGTTACCAGGGAAGCTGCCAGCAATGGCGTCTTGCTGCTCATGGAAAGG
GAAGACAAAGTGCATCTGAAGCTGGAAAGGGGCAACCTGATGGGGGGCTGGAAGTACTCCACGTTCTCGGGCTTCTTGGTTTTTCCTCTATAA
```

Human CBLN2 CDS

```
>lcl|NM_182511.4_cds_NP_872317.1_1  [gene=CBLN2]  [db_xref=CCDS:CCDS11999.1]  [protein=cerebellin-2
precursor] [protein_id=NP_872317.1] [location=578..1252] [gbkey=CDS]
ATGCAGGCGCCCGGCCGGGGGCCACTCGGGCTGCGGCTGATGATGCCCGGGCGCCGGGGGGCGCTGCGCGAGCCGGGCGGCTGCGGATCCTGCCTGG
GGGTGGCGCTGGCCCTGCTGTTGCTGCTACTGCCCGCCTGCTGCCCCGTGCGGGCGCAGAACGACACGGAGCCCATCGTGCTGGAGGGCAAGTGCCT
GGTGGTGTGCGACTCCAGCCCGTCGGCGGACGGCGCCGTCACCTCCTCCCTAGGCATCTCCGTGCGCTCCGGCAGCGCCAAGGTGGCCTTCTCCGCC
ACGCGGAGCACCAACCACGAGCCGTCCGAGATGAGCAACCGCACCATGACCATCTATTTCGACCAGGTATTAGTAAATATTGGCAACCACTTTGATC
TTGCTTCCAGTATATTTGTAGCACCGAGAAAAGGGATTTATAGCTTCAGCTTCCACGTGGTCAAAGTGTATAACAGACAAACCATCCAGGTCAGTTT
AATGCAGAATGGCTACCCAGTGATCTCGGCCTTTGCAGGAGACCAGGATGTCACCAGAGAAGCTGCTAGCAATGGCGTGCTGCTGCTCATGGAAAGG
GAAGACAAAGTGCATCTCAAACTTGAGAGAGGCAACCTCATGGGGGGCTGGAAATACTCCACATTCTCGGGCTTCTTGGTGTTTCCTCTATAA
```

Mouse CBLN2 CDS

```
>lcl|NM_172633.4_cds_NP_766221.1_1  [gene=Cbln2]  [db_xref=CCDS:CCDS29388.1]  [protein=cerebellin-2
precursor] [protein_id=NP_766221.1] [location=372..1046] [gbkey=CDS]
ATGCCCGCGCCTGGCCAGGGCCCCAGAGGGCCGCTGCTGAGCATGCCCGGGCGCCGGGGGGCGCTGCGTGAGCCAGCCGACTTTGGCTCCAGCCTGG
GGGCGGTGCTGGCCCTGCTGTTGCTGCTGCTGCCCGCCTGCTGCCCCGTAAGGGCTCAGAACGACACGGAGCCCATCGTGCTAGAGGGCAAGTGCCT
GGTAGTGTGCGATTCCAGCCCATCGGGGGATGGCGCCGTCACTTCTTCCCTGGGCATTTCTGTGCGCTCAGGCAGTGCCAAGGTGGCCTTCTCCGCT
ACTCGGAGCACCAACCACGAGCCGTCAGAGATGAGCAACCGTACCATGACCATCTACTTCGACCAGGTCTTAGTAAACATTGGCAACCACTTTGACC
TTGCCTCCAGTATATTTGTAGCACCAAGAAAGGGAATTTATAGCTTCAGCTTCCACGTGGTCAAAGTGTACAACAGACAAACTATCCAGGTCAGCTT
AATGCAGAATGGCTACCCGGTGATCTCTGCATTTGCCGGAGACCAGGATGTTACCAGGGAAGCAGCCAGCAATGGTGTTCTGCTGCTCATGGAAAGA
GAAGACAAAGTTCATCTCAAACTAGAGAGAGGCAACCTCATGGGAGGCTGGAAATACTCCACATTCTCGGGCTTCTTGGTTTTTCCTCTATAG
```

Data S2. CBLN2 promoter sequence of the tree shrew, human and mouse, related to Figure 1.

Tree shrew CBLN2 Promoter

```
>tsC2Pro KIZversion3 chr12:11145679-11147494:+ len=1816
GTCCCTTTAGTAAAGATTCTATCCATTTTCCACCCAAAAAACAAATCCCAGGAAGTTACTTAGGGTGAAAAAGACTAAAAAGGGAGAGAGAAAAAAA
AAAAAAAGCAAAGACAGAGGGACTGAGGAAGGAGCGAGAGACAGAGGGACCAAAGCCAAATACCCACTTTCAGGTCTCCGCAGCCGCTGTCTGATCC
CTGCGAGAAACCCCGCGTCCCCCTCTCTGCCAAGGGCAGGCTCCAGCGACGGGAATCCACGCTCGGCGTATTCCCCAGCTATTGTTCTGAGCCGGTC
CGGCCTCCAGGGCTGGGAGGCAGCACTTGTGGGGTTCGGCTAACTTACGCGTCCGTTTCGACTTTCCCCGTCCCTCTTCTCCCACTAAGCCAGACAG
GGCGTTTCAATTCCCAGCCCAGAAACAAAACGCACAGAAGCCTCCCCCCTCCCCGCCCACGGCCTCCGAACAACTCTTGCAATATTCTTTCCGAGAA
GACAGAAGTTGCTTCTCGGCTAACACCCTGTCGTCAGCCCAGGGCAGACCGACCGAGAGGCGCGCCCGCTGCCTCTGGCTCTTGCACCCCAGAACGC
AGGCTCGCCACGCTCCTCGTTAGCTCGTACGCAAGAAAGCCATGAAGCCACGATCCCGAAGCGCACGGCCGGGCTCCGTTACCTGGAACTGCCATGA
TGGGCGAGCACTGCTGTCCGCGAAGTTCTGCTCAGAGAAAATGAGGCGGACGGGAGCTGGCGGGAGGAGGACTCGGAGCGCGACCCTTCTGCCCGCA
CGGCCAATAACCGCGCCGCCCCGCCCTGCCGCTTTCCCGCGCCGGCCGAGCGGGCTCCGCACGCACCGACGCCCCGCACTCGTGCGCCGCTGCGCCC
GGTCCGGCCCGGGCTCTGGCCCCGGCTCCGGCCCCAGCTCCGGCCCCAGCTCCGGTCCCGTGCGCTGCGACCCGGCACCGCAGAAGCCCAGCGCCAC
TACCGAAGGCTTCGCGCCCGGCACGGAGCCCTCACCCAGCTTCGGCCGGCTGCTCCGCGCGGCCCGAGTGCTCCCTAACCCGCGTGTCCACAGCGCG
GCTCTGGCGGGGACCCTCAGTCCCAGCCTCCCCGCCAGACTCCCGAGGGCTCCGCCGAGCGCTGCCCTCACCCTGAGGACTCCGCGCACCGCGCTCC
TCCCGCCCTGATGCCCCAGCCCGCACCCGCCGCACCCTGCAACGCCCTGGCCAGGCTCTCACCCAAACACCCCGAGCGAAATCCTCCCAGACGCCGC
TAAGATTAGGCGCCCTCGGAAGCACCGGTCGCGTCTCTTCTTTTAGATTGTTTCTTTTCTTTTCCAAATTCCCTCCAAGTCTTAGTCTTGGATAGTG
TGACCACTTTCATAATGAAGGGGGGAAAAAACAGTTCTCTTAAGGAAAAGACTGAGATGGAGAGAAAACGCCTGGGGACGTAATGATGGGAGGGCTG
TGGTTCCGGAATCTCCAGTTTGGGAAATGCCAGGCTTTGGGCAGGGATCGAAGAGGTGCCTCTCTGGGTTTATGTGTCTGCTCCTCTGAACCTCCGG
GCTCGCGGAGCGCGTTGGCACTGGTGCTTATTTATTAAAATGCGGGTTCTGAGCCCTGATGACCGGGGAGACGCGCCGGGTTTCCGAGCTGCGCCTG
GAGCGCGGGCGAGACGCACAAGCACGGACGCGGGGCGCCACCTACGGGCGCTGGGGCGTCCCTGCCGCAGGCTGCGGGCAAGGACTTGGCAGAAGAG
GGGGAGTCCTAGAGACAAGGACGAGGTTTGCTGATGGGCTTTCACCCCAAAAAACAGTGCTGGCCTCTGC
```

Human CBLN2 Promoter

```
>HuC2Pro hg38 chr18:72542815-72544612 strand=+ len=1798
GTCCCTTTGGTACAGATTTTATCTGTTTTCCACCCACAGAACAAATCCCAGGAATCTCCTTGGGGCGAATAAGATTACTAGGGGGGAAATGACACCA
CACACACACACACACACACACACACACACACACACACACACACACACACGGGCAGTAAAGCCAAACACCTACTTCCAAGGTCCCTGCAGCCACTAGCGA
GCCCCCTTGGGTGAAACCCCTGGGATTCCTCTCTTAGGCGAAGGACCGATTCCAACGACGGAATCCCAGGCTTGGGTTATTCTCCAGCTCTGGTTTC
CAGACCACGGGGATTCTCTCTTTCTCAGCGGGGCGGCAGCCCCTGCAGGTTTCTGCGAACTTACGCGCCCGTCTGCACTTTTGCCCCGTCCCCGCTC
CTCCCAGGAAAGCAGACAGGCCATTTCAATACCAGCCCAGAGACACGCAGAGAAGCCGCCCCCTCGCCGCCCACAGCCTCCCATTAACTCTTCCAGT
ATTCTTTCTAAGAACAGAGAAGTTGGCTCTTGATAAATATCCGCTGTCCGCAGCCCCGATCCTACATGATTTCCCTTCTCTCTCTCCACCTCCTCCA
CCCCTCGATCTGGACCAGGGAGAGTCTTCGTTAAAATCCACGCAGAAGGCGCTTGCATGCGGAGGGGAGGGCAGGTCGGGGGTGGTTACCTGGAACA
TCCATGCTGGGCGAGCTCCGCTGTCCGCGAAGTTGCTCTGCTTAGAGAAAATGAGGCGAGTGGGAGCTGTCGGGAGGAGGACACGGAGCGCGACCCT
GCTCCCAGCGCGTGGCCAATAACCGCGCCGCCCCGCCCTGCCGCTTTCCCGCGCCAGCCTGCGCCGCTTCAGGGGTGCACCACGCCCCGCGCGCCCG
CTTAGGCGCCGCGCCCGGGACCGGGAACCCCGCGTCTCGCCCGGCTCAGCGCCCCGCGCTGTGCGCCCAGGTGCCTCCAACCCCTGGGCTTCGCGCC
CGCACCGCTGCCTGGGGCCCCTCGAGCTCCCGCGCTCAGCGCGTCCGCAGCGCGGCTCCCTCGCGGGTCCCCTCGGCCCCGCAGCCCCGCCAGTCTC
CAGAGGGCATAGCCGAGCGCTGCCGCCTCCCTAGGACTCGGAACCTTCCCTGCTCCCAAGCCCGCCGCACCCTCCAACGCCCCGGTCCTGCTCACCC
AAACGCCCAGAGCAAAATACTCCCAGACGTCTCTTTTGAAAATTAGACACCCTTAAAAGCACCGGTCGCTTCTCTTCTTTTAGATTCTTTCTTTTCT
TTCCCCAATTCCCTCCAAGTCTTAATATTGAATGGCGTGACCACTTTCATAATGACAGGAGCGAAAAAAACAATAAGTTAAAAAAAAAAAAAAAGAAG
AAGAAGACTGGGATGGCTGGGACGAAGAGAGGGAAAAAAAAAGCTTGAGAATTTGATGATTTAGGAGCTGTGGTTCCAGAGTCCCAGTTGGGGAACT
GTCCGGTTCCCGCAGGGCTAGAAGAGGGGCCTCCGGCCCGGGTCTGTGTGTCTGCTCCTCTGGACCTCGGACTGGTGGAGCGGCTGGCGCTTGCGCT
TATTTATTAAATTGCGGTGTCCGCGCTCGCTCAAAGGACCCGGGGAGACGCGCTGGGTTTCCCAGCTGCGCCTGGAGCTCGGCTGAGGGCGCAAACA
CGGACGTGGGGCGCCACCTGCGGGTGCCCGGGCCTCCCTGCCGCAGGCTGCGGACCGGCGGACTCCGACAGAAGACGGGGGAGTCCTGGCCGCAAGG
CTGAGGTTTGCTGATGGTCTTCTTTCACCCCAAAAGCAGCGCTGACCTCTGC
```

Mouse CBLN2 Promoter

```
>MsC2Pro mm39 chr18:86728752-86729905 strand=+ len=1154
CTGTTTTAGTTGGGGTGAAGGAAAAGAAAATCCTCAAACCTCACCTTTGTCCCTGGGTACTCCCCCATACTGAGTAGGCTTGTTCGCAGCCCGAGGA
CGGCAGGCTCCAGCGCCCATAGGTGGCGCCCCTCGTCCGTGCTTGCGCATCTCCCGGCTCTCCAGGCGCAGCTCGGAACCCCGCGTGTCTCCCCGGG
TCTTTAGAGCCCAGAGACTGCGTTTTAATAAATACGAGTGGTGCCAACGCGCTCCAGCCCCGGGGTTCAGAGCAGCAGACACATAAACCCAGGCAGG
AAGAGCCTCTTTAATGTCTGCCAAAAGCTGACATTTCACTAACCGGAGATTTTGGAATCGCAGTTTAAAACCATTAAATTACCGAGGCTTTTCCTCT
CTTAAAAAATACTTTTCCTTTTTTTTTTTTTTTTTTTTTTTTTTTTTGGTACCCTCTCCTCTTTAAAATTATGAATGTGGCCACTCCGTTCAAGATCA
AGACTTGGAGGGAATTTGGAAAAGAAAAAGAATCTGGAAGAAGAAACTCGGCTGGTGCTTCGGAGGGCGCCTAGTTTTAAGAAGGTACCTAAGAGAA
CTGTCCTAGATGTCTAGGTGAGAGACGGATATCGGGGCTTGGCAAGGTGCTACGGGGAAGTCACAGGGGACTCCACAGGGCTGTGGATTCTAGCACG
GAGGGAGCGAGGAGTCCTTAGTTGCCAGCTCCGAGCTGTGCTTTCTGGAGACTGGAGCTGAGGAGGGGACCCAGGGATCCTGTTATTGAGGTTCAGG
ATAGGTGTACTGACTGGGGAACTATTCCGGACCCGAAGGAGGCAAACTGCGCGCCTTGGTGCGGTGCGGCTCCTGGCTTCGGCGCGGCTCAACCCGA
GCGGGTAGCAAGTAGGTTCCAGGCACGGTCGTGCGTGCGCTGAGCAGCGCTCGGTGTGGGGCTCTCTGGGCTCTCGGGGCCGGGGCGTGGTGTGCTT
GCGGAGCGGGATCGGACCGAGAGGAGAGGAGGCAGGGCGGGGCGGAGCAGTTATTGGCCAGGCGCTGGGAGAAGGGTCGTGCTCCGAGGTGTCCTCC
TCCCGCTAGCTCCCGCCTGCCTCATTTTCTCTAAGCAGAACAACTTCTCCGACATCGGTACTCGCCCGTCATGGACATTCCAGGTAA
```

Data S3. CBLN2-promoter driven AAV vector sequences, related to Figure 2 and 3

AAV1-tsC2Pro-GFP (6345 bp)

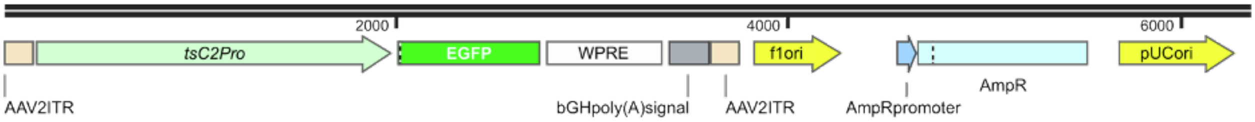

cctgcaggcagctgcgcgctcgctcgctcactgaggccgcccgggcaaagcccgggcgtcgggcgacctttggtcgcccggcctcagtgagcgagcgagcgcgcagagagg
gagtggccaactccatcactaggggttccttctagacaactttgtatagaaaagttggcagaggccagcactgtttttttggggtgaaagcccatcagcaaacctcgtcctt
gtctctaggactcccccctcttctgccaagtccttgcccgcagcctgcggcagggacgccccagcgcccgtaggtggcgccccgcgtccgtgcttgtgcgtctcgcccgcgc
tccaggcgcagctcggaaacccggcgcgtctccccggtcatcagggctcagaacccgcattttaataaataagcaccagtgccaacgcgctccgcgagcccggaggttcag
aggagcagacacataaacccagagaggcacctcttcgatccctgcccaaagcctggcatttcccaaactggagattccggaaccacagccctcccatcattacgtccccag
gcgttttctctccatctcagtcttttccttaagagaactgtttttttccccccttcattatgaaagtggtcacactatccaagactaagacttggagggaatttggaaaaga
aaagaaacaatctaaaagaagagacgcgaccggtgcttccgagggcgcctaatcttagcggcgtctgggaggatttcgctcggggtgtttgggtgagagcctggccagggc
gttgcagggtgcggcgggtgcgggctggggcatcagggcgggaggagcgcggtgcgcggagtcctcagggtgagggcagcgctcggcggagccctcgggagtctggcgggg
aggctgggactgagggtccccgccagagccgcgctgtggacacgcgggttagggagcactcgggccgcgcggagcagccggccgaagctgggtgagggctccgtgccgggc
gcgaagccttcggtagtggcgctgggcttctgcggtgccgggtcgcagcgcacgggaccggagctggggccggagctggggccggagccggggccagagcccgggccggac
cgggcgcagcggcgcacgagtgcggggcgtcggtgcgtgcggagcccgctcggccggcgcgggaaagcggcagggcggggcggcgcggttattggccgtgcgggcagaagg
gtcgcgctccgagtcctcctcccgccagctcccgtccgcctcatttctctgagcagaacttcgcggacagcagtgctcgcccatcatggcagttccaggtaacggagccc
ggccgtgcgcttcgggatcgtggcttcatggctttcttgcgtacgagctaacgaggagcgtggcgagcctgcgttctggggtgcaagagccagaggcagcgggcgcgcctc
tcggtcggtctgccctgggctgacgacagggtgttagccgagaagcaacttctgtcttctcggaaagaatattgcaagagttgttcggaggccgtgggcggggaggggga
ggcttctgtgcgttttgtttctgggctgggaattgaaacgccctgtctggcttagtgggagaagagggacggggaaagtcgaaacggacgcgtaagttagccgaaccccac
aagtgctgcctcccagccctggaggccggaccggctcagaacaatagctggggaatacgccgagcgtggattcccgtcgctggagcctgcccttggcagagaggggggacgc
ggggtttctcgcagggatcagacagcggctgcggagacctgaaagtgggtatttggctttggtccctctgtctctcgctccttcctcagtccctctgtctttgctttttttt
tttttttctctctcccttttagtctttttcaccctaagtaacttcctgggatttgtttttttgggtggaaaatggatagaatctttactaaaggaccaagtttgtacaaa
aaagcaggctgccaccatggtgagcaagggcgaggagctgttcaccggggtggtgcccatcctggtcgagctggacggcgacgtaaacggccacaagttcagcgtgtccgg
cgagggcgagggcgatgccacctacggcaagctgaccctgaagttcatctgcaccaccggcaagctgcccgtgccctggcccaccctcgtgaccaccctgacctacggcgt
gcagtgcttcagccgctaccccgaccacatgaagcagcacgacttcttcaagtccgccatgcccgaaggctacgtccaggagcgcaccatcttcttcaaggacgacggcaa
ctacaagacccgcgccgaggtgaagttcgagggcgacaccctggtgaaccgcatcgagctgaagggcatcgacttcaaggaggacggcaacatcctggggcacaagctgga
gtacaactacaacagccacaacgtctatatcatggccgacaagcagaagaacggcatcaaggtgaacttcaagatccgccacaacatcgaggacggcagcgtgcagctcgc
cgaccactaccagcagaacacccccatcggcgacggccccgtgctgctgcccgacaaccactacctgagcacccagtccgccctgagcaaagaccccaacgagaagcgcga
tcacatggtcctgctggagttcgtgaccgccgccgggatcactctcggcatggacgagctgtacaagtaaacccagctttcttgtacaaagtgggaattccgataatcaac
ctctggattacaaaatttgtgaaagattgactggtattcttaactatgttgctccttttacgctatgtggatacgctgctttaatgcctttgtatcatgctattgcttccc
gtatggctttcattttctcctccttgtataaatcctggttgctgtctctttatgaggagttgtggcccgttgtcaggcaacgtggcgtggtgtgcactgtgtttgctgacg
caacccccactggttggggcattgccaccacctgtcagctcctttccgggactttcgctttccccctccctattgccacggcggaactcatcgccgcctgccttgcccgct
gctggacaggggctcggctgttgggcactgacaattccgtggtgttgtcggggaagctgacgtcctttccatggctgctcgcctgtgttgccacctggattctgcgcggga
cgtccttctgctacgtcccttcggccctcaatccagcggaccttccttcccgcggcctgctgccggctctgcggcctcttccgcgtcttcgccttcgccctcagacgagtc
ggatctccctttgggccgcctccccgcatcgggaattcctagagctcgctgatcagcctcgactgtgccttctagttgccagccatctgttgtttgcccctcccccgtgcc
ttccttgaccctggaaggtgccactcccactgtcctttcctaataaaatgaggaaattgcatcgcattgtctgagtaggtgtcattctattctggggggtggggtggggca
ggacagcaagggggaggattgggaagagaatagcaggcatgctggggagggccgcaggaacccctagtgatggagttggccactccctctctgcgcgctcgctcgctcact
gaggccgggcgaccaaaggtcgcccgacgcccgggctttgcccgggcggcctcagtgagcgagcgagcgcgcagctgcctgcaggggcgcctgatgcggtattttctcctt
acgcatctgtgcggtatttcacaccgcatacgtcaaagcaaccatagtacgcgccctgtagcggcgcattaagcgcggcgggtgtggtggttacgcgcagcgtgaccgcta
cacttgccagcgccttagcgcccgctcctttcgctttcttcccttcctttctcgccacgttcgccggctttccccgtcaagctctaaatcgggggctccctttagggttcc
gatttagtgctttacggcacctcgaccccaaaaaacttgatttgggtgatggttcacgtagtgggccatcgccctgatagacggtttttcgccctttgacgttggagtcca
cgttctttaatagtggactcttgttccaaactggaacaacactcaactctatctcgggctattcttttgatttataagggattttgccgatttcggtctattggttaaaaa
atgagctgatttaacaaaaatttaacgcgaattttaacaaaatattaacgtttacaattttatggtgcactctcagtacaatctgctctgatgccgcatagttaagccagc
cccgacacccgccaacacccgctgacgcgccctgacgggcttgtctgctcccggcatccgcttacagacaagctgtgaccgtctccgggagctgcatgtgtcagaggtttt
caccgtcatcaccgaaacgcgcgagacgaaagggcctcgtgatacgcctatttttataggttaatgtcatgataataatggtttcttagacgtcaggtggcacttttcggg
gaaatgtgcgcggaacccctatttgtttatttttctaaatacattcaaatatgtatccgctcatgagacaataaccctgataaatgcttcaataatattgaaaaaggaaga
gtatgagtattcaacatttccgtgtcgcccttattcccttttttgcggcattttgccttcctgtttttgctcacccagaaacgctggtgaaagtaaaagatgctgaagatc
agttgggtgcacgagtgggttacatcgaactggatctcaacagcggtaagatccttgagagttttcgccccgaagaacgttttccaatgatgagcacttttaaagttctgc
tatgtggcgcggtattatcccgtattgacgccgggcaagagcaactcggtcgccgcatacactattctcagaatgacttggttgagtactcaccagtcacagaaaagcatc
ttacggatggcatgacagtaagagaattatgcagtgctgccataaccatgagtgataacactgcggccaacttacttctgacaacgatcggaggaccgaaggagctaaccg
cttttttgcacaacatgggggatcatgtaactcgccttgatcgttgggaaccggagctgaatgaagccataccaaacgacgagcgtgacaccacgatgcctgtagcaatgg
caacaacgttgcgcaaactattaactggcgaactacttactctagcttcccggcaacaattaatagactggatggaggcggataaagttgcaggaccacttctgcgctcgg
cccttccggctggctggtttattgctgataaatctggagccggtgagcgtggaagccgcggtatcattgcagcactggggccagatggtaagccctcccgtatcgtagtta
tctacacgacggggagtcaggcaactatggatgaacgaaatagacagatcgctgagataggtgcctcactgattaagcattggtaactgtcagaccaagtttactcatata
tactttagattgatttaaaacttcatttttaatttaaaaggatctaggtgaagatcctttttgataatctcatgaccaaaatcccttaacgtgagttttcgttccactgag
cgtcagaccccgtagaaaagatcaaaggatcttcttgagatcctttttttctgcgcgtaatctgctgcttgcaaacaaaaaaaccaccgctaccagcggtggtttgtttgc
cggatcaagagctaccaactctttttccgaaggtaactggcttcagcagagcgcagataccaaatactgttcttctagtgtagccgtagttaggccaccacttcaagaact
ctgtagcaccgcctacatacctcgctctgctaatcctgttaccagtggctgctgccagtggcgataagtcgtgtcttaccgggttggactcaagacgatagttaccggata
aggcgcagcggtcgggctgaacggggggttcgtgcacacagcccagcttggagcgaacgacctacaccgaactgagatacctacagcgtgagctatgagaaagcgccacgc
ttcccgaagggagaaaggcggacaggtatccggtaagcggcagggtcggaacaggagagcgcacgagggagcttccagggggaaacgcctggtatctttatagtcctgtcg
ggtttcgccacctctgacttgagcgtcgatttttgtgatgctcgtcaggggggcggagcctatggaaaaacgccagcaacgcggcctttttacggttcctggccttttgct
ggccttttgctcacatgt

Data S3. CBLN2-promoter driven AAV vector sequences, related to Figure 2 and 3, continued.

AAV9-tsC2ProT-mScarlet3-Cre (6775 bp)

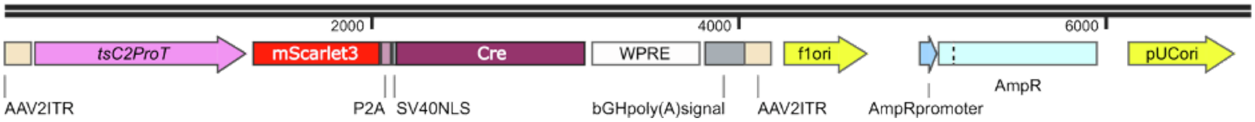

cctgcaggcagctgcgcgctcgctcgctcactgaggccgcccgggcaaagcccgggcgtcgggcgacctttggtcgcccggcctcagtgagcgagcgagcgcgcagagagg
gagtggccaactccatcactaggggttccttctagacaactttgtatagaaaagttggcagaggccagcactgtttttggggtgaaagcccatcagcaaacctcgtcctt
gtctctaggactcccctcttctgccaagtccttgcccgcagcctgcggcagggacgccccagcgcccgtaggtggcgccccgcgtccgtgcttgtgcgtctcgcccgcgc
tccaggcgcagctcggaaacccggcgcgtctccccggtcatcagggctcagaacccgcattttaataaataagcaccagtgccaacgcgctccgcgagcccggaggttcag
aggagcagacacataaacccagagaggcacctcttcgatccctgcccaaagcctggcatttcccaaactggagattccggaaccacagccctcccatcattacgtccccag
gcgttttctctccatctcagtcttttccttaagagaactgtttttcccccttcattatgaaagtggtcacactatccaagactaagacttggagggaatttggaaaaga
aaagaaacaatctaaaagaagagacgcgaccggtgcttccgagggcgcctaatcttagcggcgtctgggaggatttcgctcggggtgtttgggtgagagcctggccagggc
gttgcagggtgcggcgggtgcgggctggggcatcagggcgggaggagcgcggtgcgcggagtcctcagggtgagggcagcgctcggcggagccctcgggagtctggcgggg
aggctgggactgagggtccccgccagagccgcgctgtggacacgcgggttagggagcactcgggccgcgcggagcagccggccgaagctgggtgagggctccgtgccgggc
gcgaagccttcggtagtggcgctgggcttctgcggtgccgggtcgcagcgcacgggaccggagctggggccggagctggggccggagccggggccagagcccgggccggac
cgggcgcagcggcgcacgagtgcggggcgtcggtgcgtgcggagcccgctcggccggcgcgggaaagcggcagggcggggcggcgcggttattggccgtgcgggcagaagg
gtcgcgctccgagtcctcctcccgccagctcccgtccgcctcattttctctgagcagaacttcgcggacagcagtgctcgcccatcatggcagttccaggtaaccaagttt
gtacaaaaaagcaggctgccaccatggatagcaccgaggcagtgatcaaggagttcatgcggttcaaggtgcacatggagggctccatgaacggccacgagttcgagatcg
agggcgagggcgagggccgcccctacgagggcacccagaccgccaagctgagggtgaccaagggtggcccccctgcccttctcctgggacatcctgtcccctcagttcatgt
acggctccagggccttcacgaagcaccccgccgacatccccgactactggaagcagtccttccccgagggcttcaagtgggagcgcgtgatgaacttcgaggacggcggcg
ccgtgtccgtggcccaggacacctccctggaggacggcaccctgatctacaaggtgaagctccgcggcaccaacttccctcctgacggccccgtaatgcagaagaagacaa
tgggctgggaagcatccaccgagcggttgtaccccgaggacgtcgtgctgaagggcgacattaagatggccctgcgcctgaaggacggcggccgctacctggcggacttca
agaccacctacagggccaagaagcccgtgcagatgcccggcgccttcaacatcgaccgcaagttggacatcacatcccacaacgaggactacaccgtggtggaacagtacg
aacgctccgtggcccgccactccaccggcggctccggtggctccggaagcggagccacgaacttctctctgttaaagcaagcaggagatgttgaagaaaaccccggccta
tgggcccaaagaagaagagaaaggtttcgaatttactgaccgtacaccaaaatttgcctgcattaccggtcgatgcaacgagtgatgaggttcgcaagaacctgatggaca
tgttcagggatcgccaggcgttttctgagcatacctggaaaatgcttctgtccgtttgccggtcgtgggcggcatggtgcaagttgaataaccggaaatggtttcccgcag
aacctgaagatgttcgcgattatcttctatatcttcaggcgcgcggtctggcagtaaaaactatccagcaacatttgggccagctaaacatgcttcatcgtcggtccgggc
tgccacgaccaagtgacagcaatgctgtttcactggttatgcggcggatccgaaaagaaaacgttgatgccggtgaacgtgcaaaacaggctctagcgttcgaacgcactg
atttcgaccaggttcgttcactcatggaaaatagcgatcgctgccaggatatacgtaatctggcatttctggggattgcttataacaccctgttacgtatagccgaaattg
ccaggatcagggttaaagatatctcacgtactgacggtgggagaatgttaatccatattggcagaacgaaaacgctggttagcaccgcaggtgtagagaaggcacttagcc
tgggggtaactaaactggtcgagcgatggatttccgtctctggtgtagctgatgatccgaataactacctgttttgccgggtcagaaaaaatggtgttgccgcgccatctg
ccaccagccagctatcaactcgcgccctggaagggatttttgaagcaactcatcgattgatttacggcgctaaggatgactctggtcagagatacctggcctggtctggac
acagtgcccgtgtcggagccgcgcgagatatggcccgcgctggagtttcaataccggagatcatgcaagctggtggctggaccaatgtaaatattgtcatgaactatatcc
gtaacctggatagtgaaacaggggcaatggtgcgcctgctggaagatggcgattagacccagctttcttgtacaaagtgggaattccgataatcaacctctggattacaaa
atttgtgaaagattgactggtattcttaactatgttgctccttttacgctatgtggatacgctgctttaatgcctttgtatcatgctattgcttcccgtatggctttcatt
ttctcctccttgtataaatcctggttgctgtctctttatgaggagttgtggcccgttgtcaggcaacgtggcgtggtgtgcactgtgtttgctgacgcaacccccactggt
tggggcattgccaccacctgtcagctcctttccgggactttcgctttccccctccctattgccacggcggaactcatcgccgcctgccttgcccgctgctggacaggggct
cggctgttgggcactgacaattccgtggtgttgtcggggaagctgacgtcctttccatggctgctcgcctgtgttgccacctggattctgcgcgggacgtccttctgctac
gtcccttcggccctcaatccagcggaccttccttcccgcggcctgctgccggctctgcggcctcttccgcgtcttcgccttcgccctcagacgagtcggatctccctttgg
gccgcctccccgcatcgggaattcctagagctcgctgatcagcctcgactgtgccttctagttgccagccatctgttgtttgcccctcccccgtgccttccttgaccctgg
aaggtgccactcccactgtcctttcctaataaaatgaggaaattgcatcgcattgtctgagtaggtgtcattctattctggggggtggggtggggcaggacagcaaggggg
aggattgggaagagaatagcaggcatgctggggaggccgcaggaacccctagtgatggagttggccactccctctctgcgcgctcgctcgctcactgaggccgggcgacc
aaaggtcgcccgacgcccgggctttgcccgggcggcctcagtgagcgagcgagcgcgcagctgcctgcaggggcgcctgatgcggtattttctccttacgcatctgtgcgg
tatttcacaccgcatacgtcaaagcaaccatagtacgcgccctgtagcggcgcattaagcgcggcgggtgtggtggttacgcgcagcgtgaccgctacacttgccagcgcc
ttagcgcccgctcctttcgctttcttcccttcctttctcgccacgttcgccggctttccccgtcaagctctaaatcgggggctccctttagggttccgatttagtgcttta
cggcacctcgaccccaaaaaacttgatttgggtgatggttcacgtagtgggccatcgccctgatagacggtttttcgccctttgacgttggagtccacgttctttaatagt
ggactcttgttccaaactggaacaacactcaactctatctcgggctattcttttgatttataagggattttgccgatttcggtctattggttaaaaaatgagctgatttaa
caaaaatttaacgcgaattttaacaaaatattaacgtttacaattttatggtgcactctcagtacaatctgctctgatgccgcatagttaagccagccccgacacccgcca
acacccgctgacgcgccctgacgggcttgtctgctcccggcatccgcttacagacaagctgtgaccgtctccgggagctgcatgtgtcagaggttttcaccgtcatcaccg
aaacgcgcgagacgaaagggcctcgtgatacgcctatttttataggttaatgtcatgataataatggtttcttagacgtcaggtggcacttttcggggaaatgtgcgcgga
acccctatttgtttatttttctaaatacattcaaatatgtatccgctcatgagacaataaccctgataaatgcttcaataatattgaaaaaggaagagtatgagtattcaa
catttccgtgtcgcccttattcccttttttgcggcattttgccttcctgtttttgctcacccagaaacgctggtgaaagtaaaagatgctgaagatcagttgggtgcacga
gtgggttacatcgaactggatctcaacagcggtaagatccttgagagttttcgccccgaagaacgttttccaatgatgagcacttttaaagttctgctatgtggcgcggta
ttatcccgtattgacgccgggcaagagcaactcggtcgccgcatacactattctcagaatgacttggttgagtactcaccagtcacagaaaagcatcttacggatggcatg
acagtaagagaattatgcagtgctgccataaccatgagtgataacactgcggccaacttacttctgacaacgatcggaggaccgaaggagctaaccgcttttttgcacaac
atgggggatcatgtaactcgccttgatcgttgggaaccggagctgaatgaagccataccaaacgacgagcgtgacaccacgatgcctgtagcaatggcaacaacgttgcgc
aaactattaactggcgaactacttactctagcttcccggcaacaattaatagactggatggaggcggataaagttgcaggaccacttctgcgctcggcccttccggctggc
tggtttattgctgataaatctggagccggtgagcgtggaagccgcggtatcattgcagcactggggccagatggtaagccctcccgtatcgtagttatctacacgacgggg
agtcaggcaactatggatgaacgaaatagacagatcgctgagataggtgcctcactgattaagcattggtaactgtcagaccaagtttactcatatatactttagattgat
ttaaaacttcatttttaatttaaaaggatctaggtgaagatcctttttgataatctcatgaccaaaatcccttaacgtgagttttcgttccactgagcgtcagaccccgta
gaaaagatcaaaggatcttcttgagatcctttttttctgcgcgtaatctgctgcttgcaaacaaaaaaaccaccgctaccagcggtggtttgtttgccggatcaagagcta
ccaactctttttccgaaggtaactggcttcagcagagcgcagataccaaatactgttcttctagtgtagccgtagttaggccaccacttcaagaactctgtagcaccgcct
acatacctcgctctgctaatcctgttaccagtggctgctgccagtggcgataagtcgtgtcttaccgggttggactcaagacgatagttaccggataaggcgcagcggtcg
ggctgaacggggggttcgtgcacacagcccagcttggagcgaacgacctacaccgaactgagatacctacagcgtgagctatgagaaagcgccacgcttcccgaagggaga
aaggcggacaggtatccggtaagcggcagggtcggaacaggagagcgcacgagggagcttccagggggaaacgcctggtatctttatagtcctgtcgggtttcgccacctc
tgacttgagcgtcgatttttgtgatgctcgtcagggggggcggagcctatggaaaaacgccagcaacgcggcctttttacggttcctggccttttgctggccttttgctcac
atgt
