# Supplementary material for: CBLN2 promoter enables genetic access to wide-field neurons of the tree shrew superior colliculus
Source: Cell Rep Methods. 2026 Mar 6;6(3):101309. doi: 10.1016/j.crmeth.2026.101309 (PMC13030960; doi:10.1016/j.crmeth.2026.101309)
Supplement: Document S2. Article plus supplemental information [file mmc3.pdf]

Cell Reports Methods

Report

CBLN2 promoter enables genetic access to wide-field neurons of the tree shrew superior colliculus

Graphical abstract

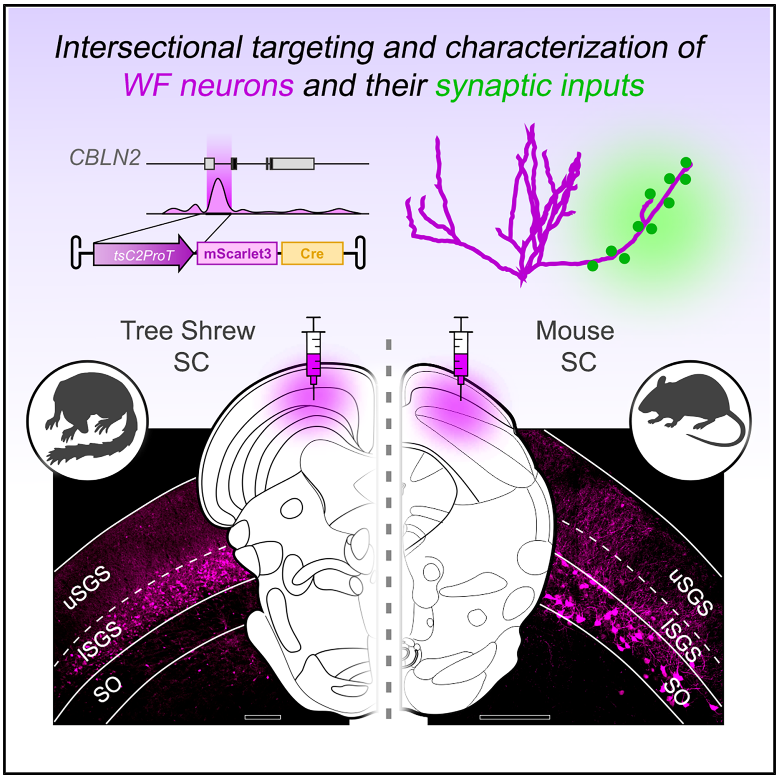

Authors

Arda Kipcak, Alev Erisir

Correspondence

erisir@virginia.edu

In brief

Kipcak and Erisir use CBLN2-promoter-based AAVs to access wide-field (WF) neurons of the SC in a pre-primate species, the tree shrew, as well as in the mouse. Using intersectional genetics, they demonstrate that tree shrew WF neurons receive retinal and cortical excitatory inputs preferentially at distal or proximal dendrites in the SC.

Highlights

- CBLN2 marks wide-field (WF) neurons in the tree shrew superior colliculus
- We develop cell-type-specific AAVs based on the tree shrew CBLN2 promoter
- These tools enable labeling and manipulation of WF neurons across species
- Tree shrew WF neurons are differentially innervated by distinct excitatory inputs

Kipcak & Erisir, 2026, Cell Reports Methods *6*, 101309
March 23, 2026 © 2026 The Authors. Published by Elsevier Inc.
https://doi.org/10.1016/j.crmeth.2026.101309

CellPress

Cell Reports Methods

CellPress OPEN ACCESS

Report

CBLN2 promoter enables genetic access to wide-field neurons of the tree shrew superior colliculus

Arda Kipcak[1] and Alev Erisir[1,2,*]
[1]Department of Psychology, University of Virginia, Charlottesville, VA 22903, USA
[2]Lead contact
*Correspondence: erisir@virginia.edu
https://doi.org/10.1016/j.crmeth.2026.101309

MOTIVATION The superior colliculus (SC) is an evolutionarily conserved midbrain structure, the function of which has adapted across species to meet specific sensory demands. Despite the conservation of cardinal SC cell types, our understanding of their contributions to behavior remains limited, in part due to a lack of cell-type-specific tools in higher-order mammals in which transgenic approaches are not feasible. To start addressing this gap, we devised a viral genetic approach to target one such cell type, termed the wide-field neurons, in the tree shrew and mouse.

SUMMARY

Wide-field (WF) neurons of the tectopulvinar pathway integrate retinal and cortical inputs via large dendritic arbors crucial for rapid visual motion detection. Previous studies identified potential marker genes for mouse WF neurons. Here, we validate CBLN2 as a molecular marker of the tree shrew WF neurons and construct AAVs that exploit CBLN2 promoter to selectively target WF neurons across species. Using intersectional genetics in the tree shrew, we show that WF neuron dendrites receive a distinct pattern of VGluT1+ and VGluT2+ inputs based on their distance from the cell body in the dorsoventral axis of the superior colliculus (SC). This represents the first example of a viral tool derived from the tree shrew genome for cell-type-specific targeting across species. Our results provide a foundation for studying SC circuitry in higher-order mammals and for extending this approach to additional conserved cell types in the SC and other brain regions.

INTRODUCTION

The superior colliculus (SC) is a topographically organized midbrain center critical for visually guided behaviors.[1] SC cell types have been classified using morphological, physiological, and, more recently, transcriptomic approaches,[2] with most insights derived from mice due to available genetic tools. In particular, Cre-recombinase-expressing transgenic lines have enabled the targeting of four principal superficial SC (sSC) cell types—stellate (RORB),[3,4] horizontal (GAD2),[5–7] narrow-field (GRP),[6–9] and wide-field (NTSR1) neurons[5,7–10]—but were not systematically generated to target these cell types specifically, often resulting in off-target labeling of additional neuronal populations.[7,9,11,12] Moreover, such genetic accessibility in mice is not readily transferable to higher-order mammals. Recent advances in viral vector technologies, including novel capsid variants[13,14] and gene regulatory elements such as promoters and enhancers,[15–20] offer new opportunities for cell-type-specific targeting, yet current tools remain largely limited to forebrain cell types, leaving SC unexplored.

Among the main sSC cell types, wide-field (WF) neuron somata are located at the lower stratum griseum superficiale (lSGS) and stratum opticum (SO), with expansive dendritic arbors and projections to the pulvinar nucleus (lateral posterior [LP] in rodents) of the thalamus.[21] Unlike the geniculostriate pathway[22–24] that is more specialized in object recognition, tectopulvinar pathway[25–28] is associated with object detection and motion localization in the visual field,[21] functions critical for survival. WF neurons, found across a wide range of species, including amphibians,[29,30] avians,[31,32] rodents,[3,5,6,8,10] prosimians,[26,33–36] and non-human primates,[37–42] are thought to extract motion saliency by integrating retinal and cortical inputs, thereby guiding behaviors such as predator avoidance and prey capture.[5,6,8,12,43–48] Accessing WF neurons outside mice typically requires retrograde labeling from the pulvinar, a complex hub interconnected with multiple brain regions,[49–52] often necessitating dual adeno-associated virus (AAV) strategies such as combining a Cre-expressing retrograde AAV in the pulvinar with a Cre-dependent AAV in the SC to selectively label WF neurons. This approach further demands high stereotaxic precision

© 2026 The Authors. Published by Elsevier Inc.
This is an open access article under the CC BY license (http://creativecommons.org/licenses/by/4.0/).

to align the topographic maps in both structures to ensure co-expression of the transgene in the same cells via both retrograde and anterograde routes. These challenges underscore the need for simpler, locally restricted, and cross-species compatible tools to access WF neurons.

The tree shrew (*Tupaia belangeri*) is increasingly recognized as a model organism due to its close evolutionary relationship to primates and its highly developed visual system, which exhibits behavioral and synaptic features more akin to those of primates.[53,54] Moreover, its relative accessibility compared to primates makes it an ideal bridge species for the development of cross-species neurobiological tools, aligning with the principles of refinement and reduction in animal research.

Building off of recent transcriptomic studies identifying Cerebellin 2 (*CBLN2*) as a WF neuron marker in mice,[7,9,10,55] in the present study, we validated CBLN2 as a conserved molecular signature for WF neurons in the tree shrew. We then identified the tree shrew CBLN2 promoter and used it to engineer cell-type-specific AAVs to target WF neurons across species. For proof of principle, we used these AAVs to map the spatial distribution of excitatory inputs on WF neurons in the tree shrew SC, revealing unique connectivity patterns of putative retinal and cortical inputs on WF dendrites. Together, our findings establish CBLN2 as a conserved marker of WF neurons and demonstrate the utility of CBLN2-promoter-driven AAVs as effective tools for genetic access to this cell type beyond rodents. By enabling precise targeting of WF neurons in the tree shrew, this study lays the groundwork for dissecting the structural and functional architecture of the tectopulvinar circuit.

RESULTS

Identification of tree shrew CBLN2 promoter

Recent molecular and transcriptomic studies of the mouse superior colliculus identified a number of genes including Nephronectin (NPNT) and CBLN2 as potential molecular markers of WF neurons.[9,10,44,55–59] We asked whether these markers are conserved in tree shrew and could enable genetic access to WF neurons. Using immunohistochemistry and single-molecule RNA fluorescent *in situ* hybridization (smRNA-FISH), we examined NPNT and CBLN2 expression in tree shrew SC. NPNT+ cells were broadly distributed across SGS, without any clear sublayer selectivity (Figures 1A and 1C), whereas CBLN2+ cells were primarily localized to the lSGS (Figures 1B and 1D), where the tree shrew WF neurons reside[35] (Figure S1G). This led us to focus on CBLN2 as a candidate marker.

The tree shrew CBLN2 coding sequence is highly conserved between human (mRNA: 93%, protein: 96%) and mouse (mRNA: 89%, protein: 95%) (see method details for Transcript IDs; see also Data S1). To identify transcriptional *cis*-regulatory elements, we compared the 5′ UTR, upstream to the coding sequence across tree shrew and human loci, and noted a 3.7 kb conserved region in the vicinity of exon 1/transcription start site. To further delineate this region, we used publicly available human brain chromatin accessibility (ATAC-seq and DNAse-seq) and histone ChIP-seq data (see key resources table for Study IDs). Alignment of the epigenomic data to CBLN2 locus revealed that this region was enriched for histone markers H3K4Me3 and H3K27Ac as well as ATAC and DNase hypersensitivity peaks, which are known to be positioned at active promoter/enhancer regions[60–62] (Figures 1F and 1G). Similarly, ENCODE cCRE database indicated a number of candidate enhancer and promoter-like elements concentrated in this region (Figure 1G). Integrating this information with cross-species alignment, we defined the boundaries, designated a 1.8 kb sequence as the putative CBLN2 promoter, and mapped this sequence to the tree shrew genome; this sequence is referred to as tsC2Pro from here on (Figure 1E; see also Data S2). Further analysis of tsC2Pro using JASPAR database (see method details) revealed a high density of transcription-factor-binding motifs shared between the tree shrew and the human (Figures 1I and 1K; see also Data S4), suggesting that transcriptional regulation of CBLN2 is conserved in higher mammals. Accordingly, cross-species comparison of the putative promoters showed 76% homology between human and tree shrew and 53% homology between human and mouse (Figure 1J). In light of these, we posited that tsC2Pro may house the core promoter and potentially other *cis*-enhancers of the locus and thus can be used to gain genetic access to WF neurons.

CBLN2 promoter directs selective reporter expression in tree shrew WF neurons

To test the hypotheses that (1) tsC2Pro drives CBLN2-specific transgene expression *in vivo* and (2) the tsC2Pro-driven virus-labeled cells are WF neurons, an AAV vector encoding tsC2Pro upstream of a GFP cassette (AAV1-tsC2Pro-GFP) was stereotaxically injected into adult tree shrew SC (Figure 2A). Three to four weeks following the surgery, coronal brain sections were prepared for confocal imaging. The AAV injection site showed a distinct band of GFP+ cells localized to lSGS at approximately 750 μm depth from the SC surface (Figures 2B, 2C, and S1). Co-localization analysis using smRNA-FISH with probes against tree shrew CBLN2 transcript revealed a high degree of specificity of AAV1-tsC2Pro-GFP: 80% ± 2.16 of the GFP+ cells were CBLN2+. Similarly, 78% ± 2.96 of CBLN2+ cells in analyzed areas expressed GFP, suggesting a high degree of sensitivity of AAV1-tsC2Pro-GFP for CBLN2+ cells (Figure 2E), indicating that tsC2Pro drives CBLN2-specific transgene expression *in vivo*. Furthermore, the pulvinar nucleus, which receives WF neuron projections, contained GFP-labeled fibers appearing as distinct clustered boutons in both central pulvinar (Pc) and dorsal pulvinar (Pd), as well as diffuse unitary boutons only in Pd (Figures 2F and S4). GFP-labeled terminals in the pulvinar nucleus contained vesicular glutamate transporter type 2 (VGluT2; Figures 2J and 2K), which is characteristic for the WF boutons.[28] Thus, the CBLN2-promoter-driven AAV-labeled cells are WF neurons. Together, these results demonstrate that AAV1-tsC2Pro-GFP effectively and selectively targets CBLN2+ WF neurons in tree shrew SC.

CBLN2-promoter-driven Cre expression allows intersectional targeting of WF neurons

After confirming that AAV1-tsC2Pro-GFP drives reporter expression in CBLN2+ WF cells, we optimized our design to enable intersectional genetics. Conventional AAV serotypes have a 4–5 kb packaging limit,[63] restricting payload to a single function, in this case, fluorescent labeling. To expand its utility, we truncated the promoter from 1.8—1.2 kb (tsC2ProT, see method details)

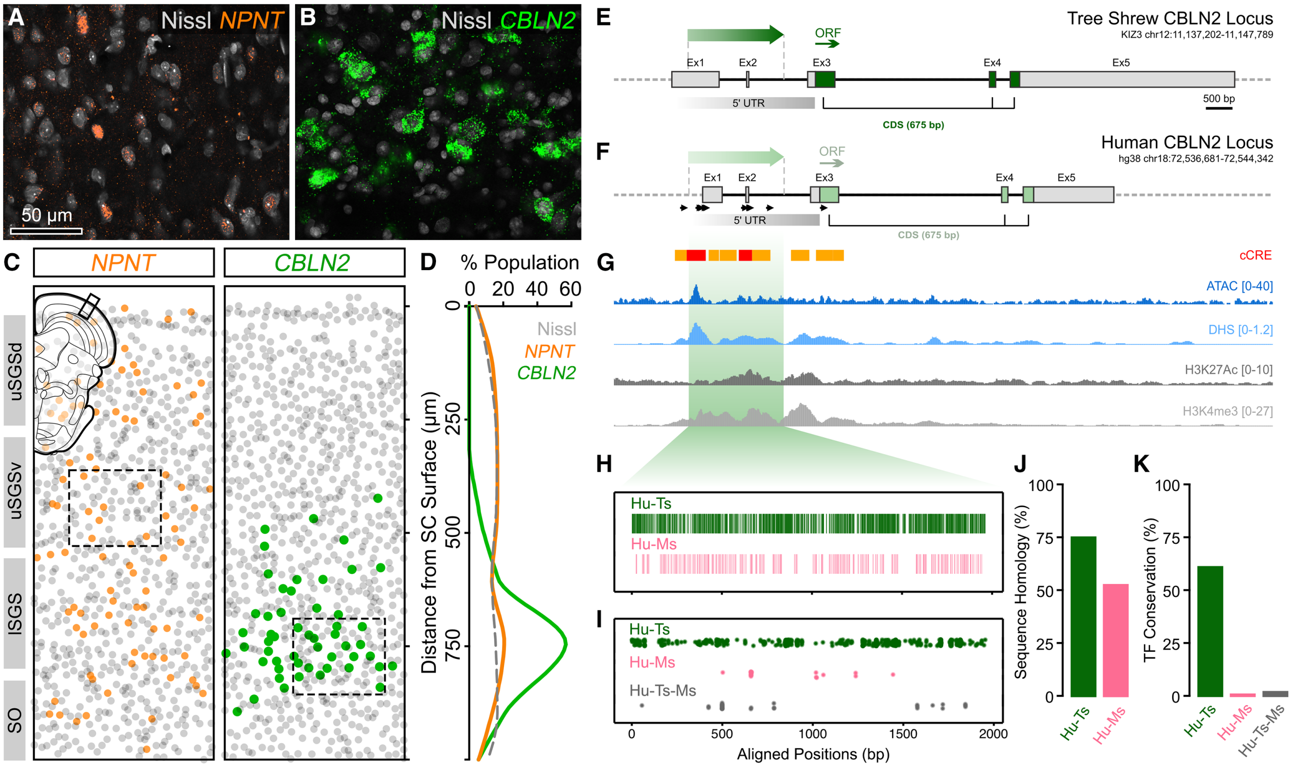

Figure 1. CBLN2 locus shares conserved features across species

(A and B) Confocal images of Nephronectin (NPNT) IHC (A) and Cerebellin 2 (CBLN2) FISH (B) in tree shrew sSC.

(C and D) Spatial and relative distributions of NPNT+ (orange) and CBLN2+ (green) cells across sSC depth, revealing clustering of CBLN2+ cells at the lSGS/SO border. Gray, Nissl-stained cells.

(E) Tree shrew CBLN2 locus showing the human-aligned promoter (dark green arrow, tsC2Pro), annotated exons (gray), and protein-coding sequence (green). Genomic coordinates are indicated in the upper-right subtext. ORF, open reading frame; 5′ UTR, 5′ untranslated region.

(F) Human CBLN2 locus with the light green region identified as the promoter (huC2Pro) and the mapped transcription start sites (black arrows) from 5′CAGE data (FANTOM5).

(G) Human cCRE, ATAC, DNAse hypersensitivity (DHS), H3K27Ac, and H3K4Me3 peaks aligned to the CBLN2 locus. Scale range is shown in brackets at the right.

(H) Multiple sequence alignment of tree shrew (Ts) and mouse (Ms) homologs to the human promoter showing pairwise sequence identity between human and tree shrew (Hu-Ts, green) and human and mouse (Hu-Ms, pink). Each bar represents a conserved base.

(I) Transcription factor (TF) enrichment plot showing the aligned position of human TF binding sites that are present in tree shrew (green), mouse (pink), or both (gray).

(J and K) Quantification of overall DNA sequence homology between the human promoter and aligned tree shrew (green) or mouse (pink) sequences (J) and shared TF-binding sites (K). 100% represents the complete human sequence.

See also Data S4.

and replaced GFP with the brighter, red-shifted reporter mScarlet3[64] and inserted Cre recombinase. We incorporated these elements into a bicistronic construct expressing mScarlet3 and Cre separated by a P2A element, packaged as AAV9 (AAV9-tsC2ProT-mScarlet3-Cre). To validate Cre function, we injected AAV9-tsC2ProT-mScarlet3-Cre along with a Cre-dependent EYFP expression vector under the control of the ubiquitous promoter Ef1a (AAV1-EF1a-DIO-EYFP, 1:1) into tree shrew SC (Figure 3A). Both reporters were expressed, and EYFP colocalized with mScarlet3 in lSGS, confirming Cre compatibility for intersectional approaches (Figures 3B and 3C).

To rule out non-specific recombination events resulting in spontaneous expression of Cre-dependent transgene in the absence of Cre,[65–67] we quantified the percentage of Cre+ cells among all EYFP+ cells using Cre IHC; virtually all EYFP+ cells were also Cre+, confirming that EYFP expression was driven by Cre-specific recombination and not an artifact (Figures 3E and 3F). Similarly, although bi-cistronic systems can show expression bias toward the first gene over the second in the open reading frame,[68,69] mScarlet3 and Cre were consistently co-expressed in our dataset (Figures 3D and 3F). Finally, specificity and sensitivity remained high despite promoter truncation (80% ± 1.67; 57% ± 3.07) (Figures 3G–3J). Thus, AAV9-tsC2ProT-mScarlet3-Cre enables intersectional strategies for functional and structural analyses of WF neurons.

Tree shrew WF neurons receive retinal and cortical excitatory inputs preferentially at distal or proximal dendrites

Tree shrew SC cells receive glutamatergic input from contralateral retinal ganglion cell axons[25,70,71] and from a variety of cortical areas.[72] While the WF neurons receive monosynaptic

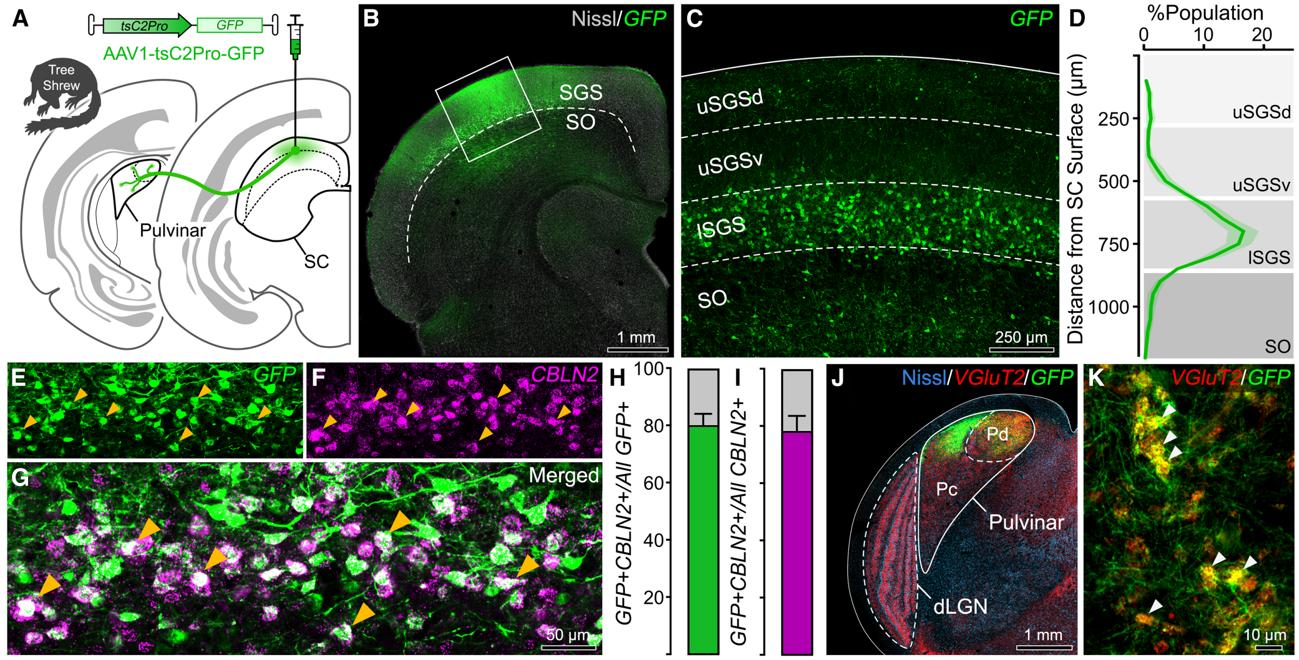

Figure 2. CBLN2-promoter-driven AAV selectively labels WF neurons of the tree shrew SC

(A) Experimental scheme for injecting AAV-tsC2Pro-GFP into tree shrew SC. Labeled WF neurons project to the pulvinar.

(B) Confocal image of the injection site with AAV-expressed GFP (green) and Nissl (gray) labels. Dashed line = lSGS/SO border.

(C) Higher magnification view showing GFP-expressing cells localized to lSGS.

(D) Distribution of the GFP+ cell labeling across superficial SC (% of total GFP+ cells).

(E–G) Higher magnification of GFP (E) and CBLN2 RNA-FISH (F) labeling in lSGS, showing a high degree of colocalization (G).

(H) AAV specificity: GFP+CBLN2+ cells as % of all GFP+ cells (mean ± SEM; 2,466 cells from three tree shrews).

(I) AAV sensitivity: GFP+CBLN2+ cells as % of CBLN2+ cells (same dataset as H).

(J) Confocal image of a thalamic section showing selective tectopulvinar axonal labeling (green) in pulvinar. Nissl (blue) and VGluT2 (red) IHC mark intra- and internuclear borders of dorsal pulvinar (Pd), central pulvinar (Pc), and dorsolateral geniculate nucleus (dLGN).

(K) Higher magnification view of pulvinar showing GFP-labeled tectopulvinar terminals that are colocalized with VGluT2. Note the "grape-wine" appearance of clustered terminals, a characteristic of "specific" tree shrew WF projections (see also Figure S4).

input from both these sources,[9,43,59,73–75] the spatial organization of these inputs as well as any pattern of selectivity to WF dendrites remain unexplored. As a proof-of-concept experiment, we used our intersectional AAV strategy (co-injection of AAV9-tsC2ProT-mScarlet3-Cre and AAV9-CAG-FLEX-tdTomato), which led to ubiquitous expression of red fluorescent proteins selectively in WF neurons. In addition, we used VGluT2 and VGluT1 IHC to visualize putative retinal and cortical axonal terminals in 100–200-µm-thick coronal sections (Figures 4A and 4B). WF neurons, located in lSGS, have large somata (200–300 µm^2) with lateral and apical dendrites that span over 1 mm lateromedially along the entire depth of the sSC (Figures 4C and 4D). As WF dendrites ramify along the dorsoventral axis, the dendrite caliber is thickest in lSGS and thinnest in the upper SGS dorsal (uSGSd) (Figure 4P). While dendritic arbor reconstructions were constrained by tissue thickness and sectioning plane, numerous dendrite branches originating from somata extended into uSGSd, forming characteristic tufts of this cell type; additional dendritic terminations were observed as tufted endings within the upper SGS ventral (uSGSv).

Consistent with the previous work,[76] the pattern of VGluT2 and VGluT1 staining displayed reciprocal dorsoventral gradients, with VGluT2 enriched in uSGSd and VGluT1 in lSGS (Figures 4B and S1A–S1C). Quantitatively, VGluT1 bouton density increased with depth and differed significantly across sublamina (Figure 4K; uSGSd: 1 ± 0.1, uSGSv: 3 ± 0.2, lSGS: 7 ± 0.25 boutons/100 µm^3; one-way ANOVA with Tukey's, $p < 0.0001$ for all comparisons). Similarly, VGluT2 bouton density was highest in uSGSd and decreased ventrally (Figure 4L; uSGSd: 8 ± 0.21, uSGSv: 6 ± 0.22, lSGS: 6 ± 0.21 boutons/100 µm^3; one-way ANOVA with Tukey's, $p < 0.0001$, for all comparisons).

To map putative synaptic appositions on WF dendrites, high-resolution confocal z stacks were acquired from uSGSd, uSGSv, and lSGS (Figure 4G), capturing distal tufts, middle dendritic arbor, and proximal dendrites, respectively. Individual AAV-labeled dendrite segments in each sublamina were reconstructed and appositions by VGluT1 and VGluT2 (i.e., putative cortical and retinal synapses) on WF dendrites were identified (Figures 4H–4J and S2; see method details for the pipeline). VGluT1 appositions were rare in the uSGSd, mirroring the low overall density of boutons there (Figure 4M). However, disproportionate to the overall bouton density, VGluT1 boutons formed appositions on dendrites with high frequency in uSGSv and lSGS, suggesting VGluT1 inputs are situated to exert a selective

CellPress
OPEN ACCESS

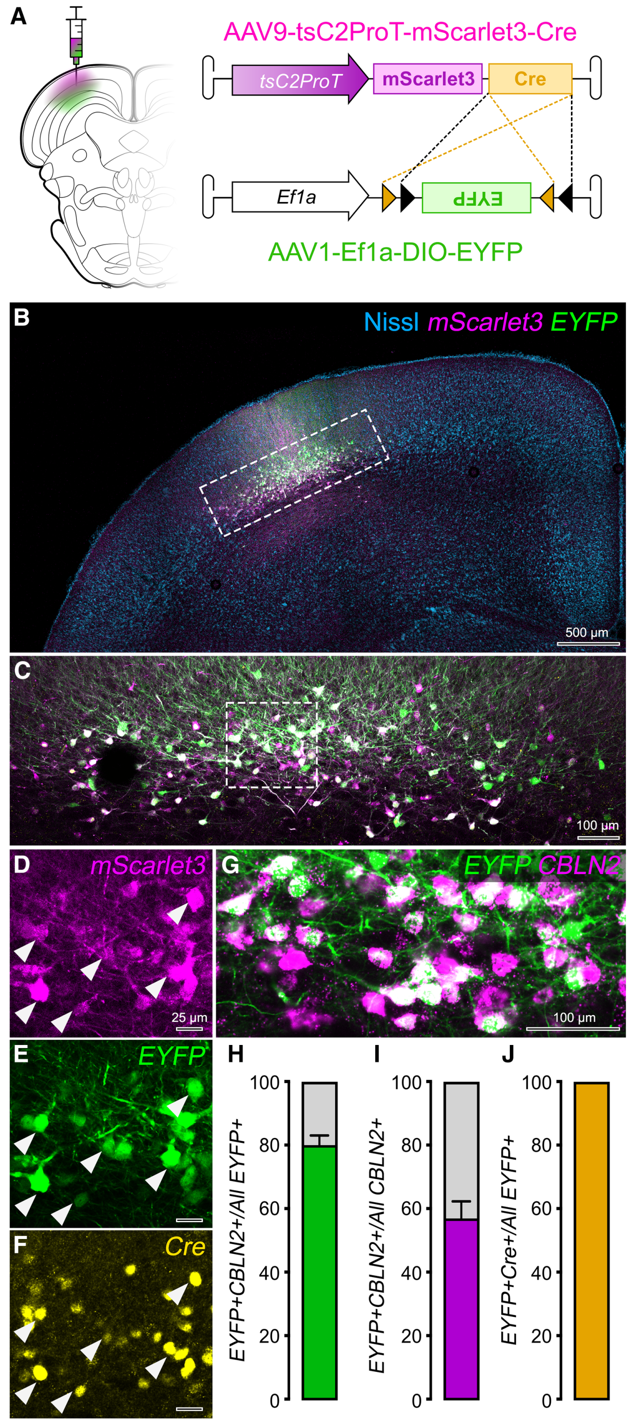

Figure 3. tsC2ProT-driven Cre expression allows efficient intersectional labeling of the tree shrew WF neurons

(A) Experimental scheme for co-injection of mScarlet3+Cre-expressing truncated CBLN2-promoter-driven AAV and Cre-dependent EYFP-expressing Ef1a-driven AAV into tree shrew SC.

(B and C) Low- (B) and high-magnification (C; rectangle in B) confocal images of the injection site showing mScarlet3 and EYFP expression. Note the oblique soma morphology typical of WF neurons.

(D–F) Higher magnification view of area marked in (C), showing endogenous expression of mScarlet3 (D), colocalized EYFP (E), and Cre IHC (F).

(G) Confocal image showing colocalization of EYFP in CBLN2+ cells.

(H) AAV specificity: EYFP+CBLN2+ cells as a percentage of all EYFP+ cells. Total of 1,817 cells from three images of one tree shrew.

(I) AAV sensitivity: EYFP+CBLN2+ cells as percentage of all CBLN2+ cells. Same dataset as in (H).

(J) Cre-driven recombination specificity: EYFP+Cre+ cells as percentage of all EYFP+ cells. The dataset contained 762 cells from three images.

and strong influence on WF excitability via synapses on proximal dendrites (uSGSd: 1.57 ± 0.31, uSGSv: 8.76 ± 0.76, lSGS: 11.05 ± 1.09 VGluT1/100 μm dendrite; one-way ANOVA with Tukey's, $p < 0.0001$ for uSGSd vs. uSGSv and uSGSd vs. lSGS; $p = 0.1013$ for uSGSv vs. lSGS). A similar mismatch was also evident for VGluT2 inputs: VGluT2 apposition frequency was uniform across all three sublamina, despite an overall bouton density gradient of VGluT2 (Figure 4N; uSGSd: 18 ± 1.17, uSGSv: 18 ± 0.87, lSGS: 19 ± 1.74 VGluT2/100 μm^3; one-way ANOVA with Tukey's, uSGSd vs. uSGSv $p = 0.9333$; uSGSd vs. lSGS $p = 0.9266$; uSGSv vs. lSGS $p = 0.7585$). That is, despite the dorsoventral decrease in VGluT2 boutons across SGS sublamina, VGluT2 appositions are as frequent on proximal dendrites (i.e., in lSGS) as they are on distal tufts (i.e., in uSGSd), suggesting that retinal terminals are situated to influence WF cell excitability via inputs throughout its dendritic arbor.

To assess relative cortical vs. retinal innervation of WF dendrite segments, we used a ratiometric index, plotting VGluT2 as a percentage of all glutamatergic inputs (VGluT1+VGluT2) for both the WF appositions and the overall terminals (Figure 4O). While retinal appositions dominated in the uSGSd, cortical inputs nearly matched the prevalence of retinal inputs in uSGSv and lSGS, suggesting strong influences by both cortex and retina via synapses close to soma, and a selective role for retinal inputs in determining WF neuron's large receptive fields through synapses on the fanned-out distal dendrites. In addition, only in lSGS, relative innervation of WF neurons by cortical vs. retinal boutons could not be predicted by overall relative presence of these boutons (WF-apposed uSGSd: 91.6% ± 1.65, uSGSv: 68.1% ± 1.77, lSGS: 62.3% ± 1.83; overall uSGSd: 90% ± 1.1, uSGSv: 66.94% ± 1.84, lSGS: 36.1% ± 0.75; two-way ANOVA with Sidak's, uSGSd $p = 0.9070$; uSGSv $p = 0.9680$; lSGS $p < 0.0001$), providing further evidence for selectivity of retinal axons for WF neurons over other possible targets in lSGS.

Tree shrew CBLN2 promoter also labels WF neurons of the mouse

Given the high sequence similarity of the CBLN2 loci, and prior evidence that CBLN2 marks the WF neurons of the mouse,[10,55,58,77] we tested whether tree shrew CBLN2 promoter can also label mouse WF neurons by injecting AAV1-tsC2Pro-GFP into mouse SC (Figure 5A). Three to four weeks following surgery, SC showed GFP+ cells concentrated in the SO/lSGS (Figures 5B and 3C), known location of mouse WF neurons.[7,10,58] Colocalization of GFP+ and CBLN2+ cells using smRNA-FISH probes against mouse CBLN2 transcript (Figure 5D) verified similarly high specificity (83% ± 0.80) and even greater sensitivity (97% ± 0.56), likely due to the lower density of WF cells in mouse

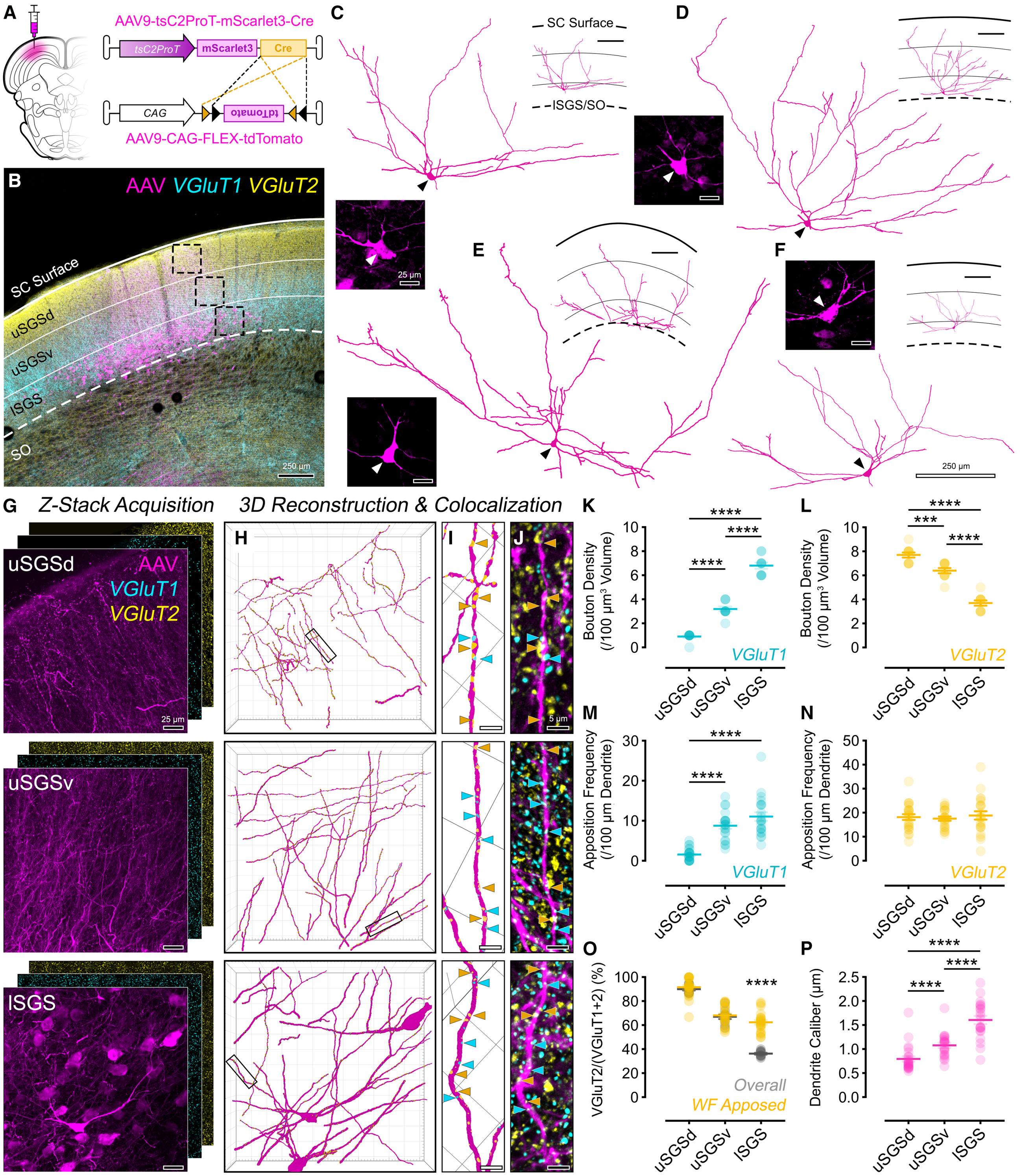

Figure 4. Tree shrew WF neuron dendrites are differentially innervated by putative retinal and cortical inputs based on distance from somata

(A) Experimental scheme for co-injection of mScarlet3+Cre-expressing truncated CBLN2-promoter-driven AAV and Cre-dependent tdTomato-expressing CAG-driven AAV into tree shrew SC.

(B) Confocal image of the injection site showing mScarlet3/tdTomato expression (magenta), VGluT1 (cyan), and VGluT2 IHC (yellow). Note the reciprocal staining patterns: VGluT1 enriched in lSGS and VGluT2 in uSGSd.

(legend continued on next page)

SC, affording higher coverage in a smaller volume. We verified the selective axonal labeling of WF projections in the mouse LP nucleus (pulvinar homolog) (Figure 5F). Similarly, as in the tree shrew, these boutons were VGluT2+. Lastly, we also tested the intersectional strategy by injecting the mouse SC with AAV9-tsC2ProT-mScarlet3-Cre and AAV1-Efa1-DIO-EYFP (1:1) and performed the same analysis (Figure 5K). Both AAVs were expressed as visualized by mScarlet3 and EYFP fluorescence (Figure 5L). We also verified Cre co-expression in mScarlet3+EYFP+ cells (Figures 5M–5P and 5T). Colocalization analysis with CBLN2 RNA showed 67% ± 0.55 specificity and 89% ± 0.66 sensitivity. Collectively, these data show that tree shrew CBLN2 promoter can be used to target mouse WF neurons.

DISCUSSION

In this study, we validate CBLN2 as a WF neuron marker, identify its promoter, and characterize viral tools using this promoter to access WF neurons of the tree shrew and mouse. AAVs described here enable local genetic access to WF neurons, offering new means to assay the structure and function of these cells. Additionally, these AAVs represent the first viral tools derived from the tree shrew genome that affords cell-type-specific genetic access to a conserved SC neuron population across species. With these advancements, several outstanding questions remain to be answered.

Role of CBLN2 in WF neurons

CBLN2 is a secretory signaling protein expressed widely across the brain.[78–82] Acting as a transsynaptic adapter, CBLN2 connects the presynaptic neurexins (*NRXN1/3*) with postsynaptic delta-type glutamate receptors (*GRID1/2*).[79,82] Shibata et al. (2021) reported a developmental role for CBLN2, where elevated CBLN2 expression promoted spine formation in mouse PFC.[83] Mechanistically, Nrxn1^{SS4+}-CBLN2 complexes enhance NMDAR-mediated transmission in mouse PFC, and conditional CBLN2 deletion markedly reduces NMDAR-EPSCs,[79] suggesting that CBLN2 is critical for efficient synaptic signaling. Given the role of tectopulvinar circuitry in rapid detection of predators and preys, CBLN2 may not only serve as a selective marker but also contribute functionally to WF neuron's excitability as a presynaptic and/or postsynaptic agent.

Influence of retinal and cortical inputs on WF dendritic integration

Our input-mapping results indicate that although VGluT2 terminals are less abundant than VGluT1 in the lSGS, WF dendrites there receive numerous VGluT2 contacts, often surpassing VGluT1. The strong VGluT1 presence in lSGS and uSGSv aligns with earlier reports that WF neurons are more likely than other SC cell types (narrow-field, stellate, and horizontal) to receive V1 input.[74] Our results further reveal that retinal contacts are positioned to provide excitation along the entire dorsoventral span of WF dendrites. Consistent with this, Kühn et al. (2025) confirmed the strong excitatory role of retinal inputs, while suggesting that the V1 input may play a minimal role in the dendritic integration.[43] However, it is important to note that Kühn et al. study was conducted in V1-excised animals. Notably, no study has exhaustively mapped the cortical inputs beyond V1 that innervate WF neurons in the tree shrew. Thus, the tools described should enable future transsynaptic mapping of WF afferents. Also to note, VGluT2 served as a proxy for all retinal inputs, without distinguishing RGC subtypes. Previous work in mice shows SC-projecting RGC subtypes vary in their laminar targeting—some innervate the entire superficial SC (ZO+SGS+SO), others project selectively to SGS, lower SGS/SO, or to a combination of these layers.[9,43,59,84] In our datasets (not quantified here), we observed diverse VGluT2 bouton morphologies across sSC, likely reflecting distinct RGC subtypes.[24] Further studies are needed to define the molecular, structural, and functional diversity of retinotectal RGCs and their targeting preferences toward WF vs. non-WF neurons.

Distinct tectopulvinar circuitries and WF neuron subtypes

Tree shrew WF circuitry exhibits features distinct from rodents: first, as shown in our study (Figure S3), tree shrew tectopulvinar axons are exclusively ipsilateral. Second, unlike rodents where bilateral and ipsilateral projections map to Pcm (tree shrew Pd) and Pl (tree shrew Pc), respectively, tree shrew tectopulvinar axons differ by innervation mode rather than subdivision. Both Pd and Pc receive topographically organized or "specific" WF inputs, but Pd subdivision additionally receives non-topographic or "diffuse" inputs.[28,33,35] Luppino et al.[33] first described this pattern, noting that Pd was always diffusely labeled regardless of SC injection site, whereas clustered bouton patches reflected

(C–F) Tracings of AAV-labeled WF neuron dendritic arbors in 80–100-μm-thick coronal sections. Insets show the somata and dendrites of traced neurons in SC sublayers. Scale bar in (F) applies to (C)–(F) and reveals that WF dendritic arbors may span over a millimeter along the lateromedial plane.
(G) Confocal *z* stacks (0.06 × 0.06 × 0.3 μm voxels) of AAV (magenta), VGluT1, and VGluT2 labeling in uSGSd, uSGSv, and lSGS. The *z* stacks from each area cover approximately 250 × 250 × 50 μm volume.
(H) 3D reconstruction of AAV-labeled WF dendrites across SC sublayers.
(I and J) 3D reconstructions (I) based on confocal volumes (J) of representative dendrite segments (rectangles in H) depicting their apposition sites (arrows) with VGluT1+ (cyan) and VGluT2+ (yellow) boutons (not depicted).
(K and L) Overall (global) VGluT1 (K) and VGluT2 (L) bouton density in each sublayer confirm the differential innervation of SGS by cortical and retinal axon boutons. $N = 10$ ROIs/lamina.
(M) Frequency of the VGluT1 appositions (putative synapses) along 100 μm length of the WF dendrite reveals that cortical input at distal dendrites is sparse yet robust at proximal segments. $N = 20\approx$ dendrites/lamina.
(N) Frequency of the VGluT2 appositions (putative synapses) along 100 μm length of the WF dendrite reveals that retinal axons densely and indiscriminately innervate dendrites in all sublayers. Same dataset as in (M).
(O) Relative density index (% ratio of VGluT2 input among all excitatory inputs) on WF dendrites (yellow) and overall (gray), showing that although VGluT1 is the predominant overall input type in lSGS, WF-VGluT2 appositions are significantly more prevalent.
(P) Calibers of WF dendrites in SGS sublayers.

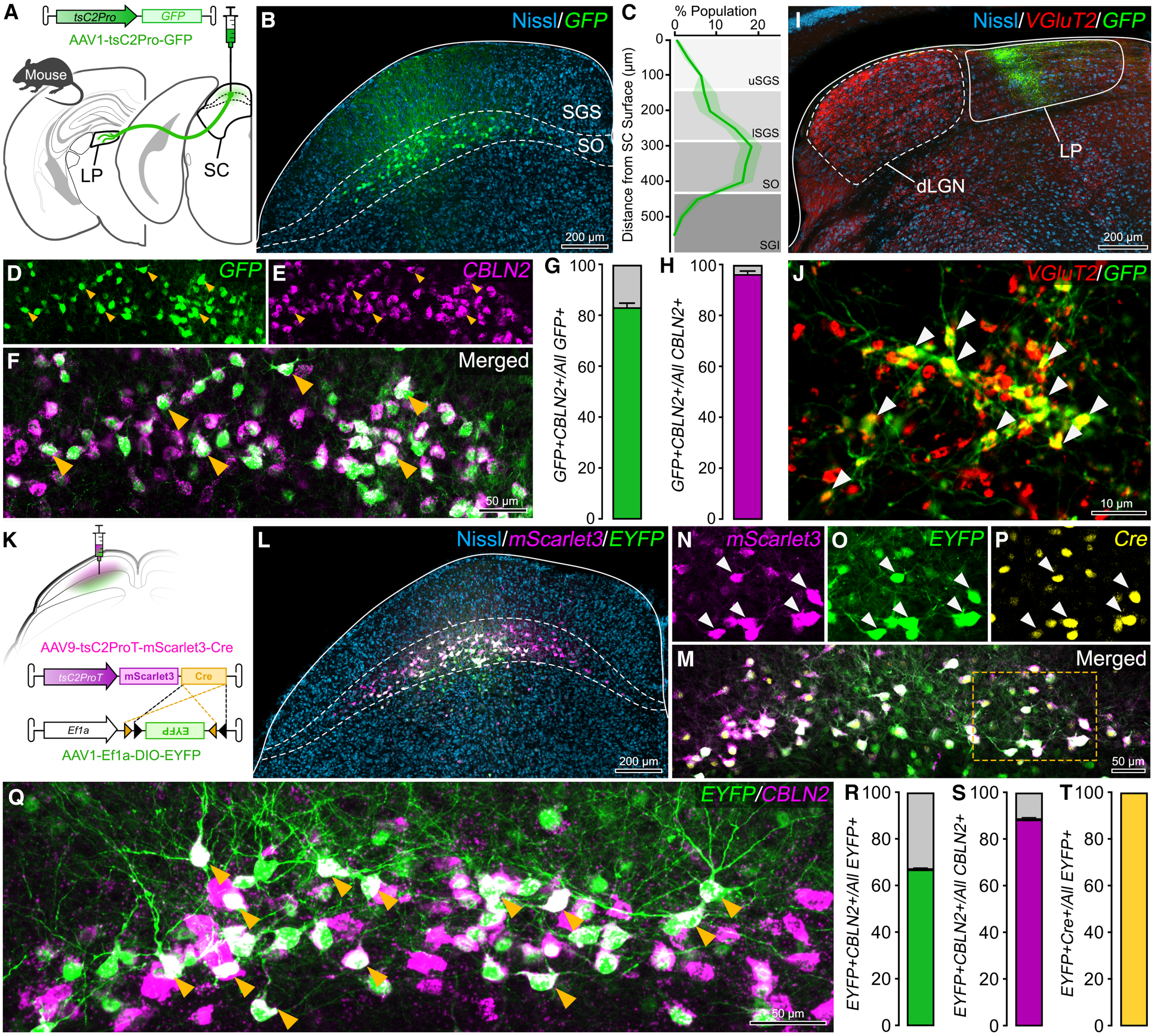

Figure 5. Tree shrew CBLN2-promoter-driven AAVs robustly target mouse WF neurons

(A) Experimental scheme for injection of AAV-tsC2Pro-GFP into mouse SC. Labeled WF neurons project to the lateral posterior nucleus (LP, pulvinar homolog) of the thalamus.

(B) Confocal image of the injection site with AAV-expressed GFP labeling (green) and Nissl staining (blue). Dashed lines indicate sSC borders.

(C) Distribution of the GFP+ cells across sSC (% of all GFP+ cells).

(D–F) Higher magnification views of GFP, CBLN2 RNA-FISH, and merged colocalization.

(G and H) AAV specificity and sensitivity: GFP+CBLN2+ cells as a % of all GFP+ (G) or CBLN2+ (H) cells. *N* = 6 images from three mice, yielding 4,252 cells.

(I) Confocal image of a thalamus section, showing selective WF axon labeling (green) in LP. Nissl and VGluT2 IHC are used to highlight intra- and internuclear borders.

(J) Higher magnification of the LP showing colocalization (arrows) of GFP-labeled WF terminals with VGluT2.

(K) Experimental scheme for the co-injection of mScarlet3+Cre-expressing truncated CBLN2-promoter-driven AAV and Cre-dependent EYFP-expressing Ef1a-driven AAV into mouse SC.

(L) Confocal image of the injection site showing mScarlet3 and EYFP expression.

(M) High-magnification image of the injection site showing mScarlet3+EYFP+ cells.

(N–P) Higher magnification view of the area marked in (M), showing endogenous expression of mScarlet3 (N), colocalized EYFP (O), and Cre IHC (P).

(Q) Confocal image showing colocalized EYFP expression with CBLN2+ cells.

(R) AAV specificity. *N* = 9 images yielding 4,196 cells from three mice.

(S) AAV sensitivity. *N* = 6 images yielding 4,252 cells from three mice.

(T) Cre-driven recombination specificity. *N* = 4 images yielding 490 cells from two mice.

retinotopy, leading to the current terminology.[33] This pattern suggests the presence of two WF subtypes, each with distinct termination types.[85] Using CBLN2-promoter-driven AAVs (Figure S4), we replicated these findings: central SC injection produced both "diffuse" Pd labeling (Figure S4B) and "specific" patches in Pc and Pd (Figures S4C–S4H). Furthermore, labeled terminals in each subdivision exhibited synapses and distinct circuitry properties, confirming that diffuse appearance of labeling in Pd reflects genuine connectivity rather than axons of passage (Figures S4I and S4J). In contrast, mouse SC injections yielded only topographic boutons in LP without clear diffuse/specific patterns, consistent with prior tracer studies.[10,57]

Because our AAV strategy relies on CBLN2 enrichment in WF neurons, an important question is whether CBLN2 marks all WF neurons or only a subset. If the latter, our vectors would preferentially label CBLN2+ subtypes. While this cannot be fully excluded, a recent study reported that a vast majority of retrogradely identified WF neurons in both mouse and tree shrew were CBLN2+,[55] supporting its suitability as a molecular target for accessing WF neurons. Taken together, CBLN2-targeted AAVs access WF neurons contributing to both projection types.

Two WF subtypes have been described in the ground squirrel: WF-I projecting bilaterally to caudal pulvinar (tree shrew Pd) and WF-II projecting ipsilaterally to rostral pulvinar (tree shrew Pc).[86] WF-I neurons occupy the lSGS with dendrites reaching the collicular surface, whereas WF-II neurons lie near the SGS/SO border with tufts in uSGSv,[86] a morphology that may constrain dendritic spread and receptive field size. The presence of bilateral and ipsilateral WF inputs to Pcm (tree shrew Pd) and Pl (tree shrew Pc) is also evident in the mouse[57] but not in primates or tree shrews. In primates, early lesion studies suggested potential contralateral tectal projections to inferior pulvinar,[87,88] but subsequent work reported strictly ipsilateral projections.[38–40,42,72,89] Consistent with primates, tree shrew SC exhibited only ipsilateral tectopulvinar projections in both anterograde and retrograde tracing experiments (Figure S3). Thus, tree shrew may represent an early stage in proto-primate evolution where cross-hemispheric tectopulvinar projections have largely disappeared.

Limitations of the study

We acknowledge a number of technical limitations of this study. First, the epigenomic datasets used were derived from human. In the absence of tree shrew data—and given its closer phylogenetic relationship to primates than rodents[90–93]—we used human epigenomic datasets assuming shared chromatin accessibility profiles. We also compared the mouse CBLN2 locus to assess cross-species conservation—a hallmark of promoters/enhancers and transcription-factor-binding sites.[94–96] The human brain epigenetic data used to help identify *cis*-regulatory elements are derived from cortex, rather than SC; ideally, tree shrew SC multi-omics datasets would be preferable but were unavailable. We therefore relied on human cortical data, assuming promoter use would be global and vary less by brain region compared to enhancers.[83] Notably, a previous study identified a developmentally restricted PFC-specific CBLN2 enhancer (E2), which was active only during mid-fetal cortical development,[83] whereas the putative promoter identified in the same study—which partially overlaps with the promoter identified in our study—exhibits constitutive DNA accessibility in the mouse, and H3K27Ac marks in the human, indicating consistent CBLN2 promoter activity across developmental time points. Our study focused on adult SC and did not assess viral expression in other regions or at earlier time points.

Second, the endogenous GFP signal from AAV1-tsC2Pro-GFP was weak and required IHC amplification, likely reflecting the strength of the promoter or a serotype-dependent effect in the tree shrew. In contrast, AAV9-tsC2ProT-mScarlet3-Cre exhibited robust endogenous mScarlet3 expression without amplification.

Third, WF neuron tracings were limited by tissue thickness; we optimized tissue thickness to include a large axial volume without compromising high-magnification confocal imaging using a 40× objective with a travel range of ∼180 μm (z stack volume). Initial light-sheet imaging of cleared intact SC at 20× lacked sufficient resolution to capture thin dendrites in upper SGS, prompting a switch to confocal imaging. While fluorescent labeling enabled multiplexed AAV and input marker visualization for a proof-of-concept approach, input mapping was limited to structural imaging; future work should include larger volume datasets and physiological validation.

Finally, we acknowledge the possibility of off-target AAV expression in non-WF populations. Previous work with enhancer-driven AAVs indicates a large range of achieved maximal specificity and sensitivity, particularly with Cre-expressing vectors.[20] Thus, we recognize that the identity of labeled neurons is putative, underscoring the need for future physiological characterization of visual responses and associated behaviors. Additionally, low-level CBLN2 expression in non-WF cells cannot be excluded; indeed, we detected, although less robust, CBLN2 transcripts outside lSGS (data not shown), suggesting that some labeled cells represent on-target, non-WF CBLN2+ populations. Nevertheless, using lower viral titers (10^{9-10} vg/mL) minimized off-target labeling. The AAV delivery routes tested were limited to stereotaxic injections directly to brain parenchyma; future work should explore systemic routes (e.g., retroorbital or tail vein) and broader range of titers.

RESOURCE AVAILABILITY

Lead contact

Requests for further information reported in this paper should be directed to the lead contact, Alev Erisir (erisir@virginia.edu).

Materials availability

Newly generated AAV vectors in this paper are made publicly available as of the publication date as DNA intermediates through the Addgene IDs listed in the key resources table. Further inquiries regarding AAVs generated in this paper will be fulfilled upon reasonable request by the lead contact.

Data and code availability

- All genetic data are available in this paper's supplemental information. Existing, publicly available genomic and epigenomic data used in this paper are accessible at respective repositories reported in the key resources table.
- All original code is deposited to UVA DataVerse and is publicly available at https://doi.org/10.18130/V3/NEGJWP as of the publication date.

- Any additional information regarding the data and analysis reported in this paper is available from the lead contact upon request.

ACKNOWLEDGMENTS

We thank Francesca Sciaccotta, Christopher Turner, and Rebecca Roberts (UVA, Psychology) for assistance with tree shrew surgeries and handling and Yuanming Liu (UVA, Biology) and Chen Chen (UVA, Psychology) for help with smRNA-FISH and mouse surgeries. We also thank Jessica Connely, PhD (UVA, Psychology) for smRNA-FISH equipment access and Jianhua Cang, PhD (UVA, Biology and Psychology) and John Campbell, PhD (UVA, Biology) for valuable feedback. This work was supported by the NIH-NINDS U01NS122040 grant.

AUTHOR CONTRIBUTIONS

A.K. and A.E. designed the study. A.K. designed the viral vectors and conducted the experiments. A.K. and A.E. analyzed the data, interpreted the results, and designed the figures. The manuscript was drafted by A.K. and edited by A.E. A.E. secured the funding. Both authors read and approved the final version of the manuscript.

DECLARATION OF INTERESTS

The authors declare no competing interests.

DECLARATION OF GENERATIVE AI AND AI-ASSISTED TECHNOLOGIES IN THE WRITING PROCESS

During the preparation of this work, the authors used GPT4 in order to compress and improve the organization of the R code used for transcription factor analysis. After using this tool, the authors reviewed and edited the content as needed and take full responsibility for the content of the publication.

STAR★METHODS

Detailed methods are provided in the online version of this paper and include the following:

- KEY RESOURCES TABLE
- EXPERIMENTAL MODEL AND STUDY PARTICIPANT DETAILS
- METHOD DETAILS
  - Stereotaxic surgeries
  - AAV design and preparation
  - Epigenomic data visualization
  - Tissue preparation
  - Immunohistochemistry (IHC)
  - RNA fluorescent in situ hybridization (smRNA-FISH)
  - Confocal microscopy
  - WF neuron tracing
  - Electron microscopy
- QUANTIFICATION AND STATISTICAL ANALYSIS
  - Sequence alignment and transcription factor binding site analysis
  - Calculation of AAV specificity and sensitivity
  - Dendrite reconstruction and synaptic apposition analysis

SUPPLEMENTAL INFORMATION

Supplemental information can be found online at https://doi.org/10.1016/j.crmeth.2026.101309.

Received: August 7, 2025
Revised: October 29, 2025
Accepted: January 9, 2026
Published: March 6, 2026

REFERENCES

1. Basso, M.A., Bickford, M.E., and Cang, J. (2021). Unraveling circuits of visual perception and cognition through the superior colliculus. Neuron *109*, 918–937. https://doi.org/10.1016/j.neuron.2021.01.013.
2. Cang, J., Chen, C., Li, C., and Liu, Y. (2024). Genetically defined neuron types underlying visuomotor transformation in the superior colliculus. Nat. Rev. Neurosci. *25*, 726–739. https://doi.org/10.1038/s41583-024-00856-4.
3. Gale, S.D., and Murphy, G.J. (2018). Distinct cell types in the superficial superior colliculus project to the dorsal lateral geniculate and lateral posterior thalamic nuclei. J. Neurophysiol. *120*, 1286–1292. https://doi.org/10.1152/jn.00248.2018.
4. Byun, H., Lee, H.-L., Liu, H., Forrest, D., Rudenko, A., and Kim, I.-J. (2019). Rorβ regulates selective axon-target innervation in the mammalian midbrain. Development *146*, dev171926. https://doi.org/10.1242/dev.171926.
5. Gale, S.D., and Murphy, G.J. (2014). Distinct Representation and Distribution of Visual Information by Specific Cell Types in Mouse Superficial Superior Colliculus. J. Neurosci. *34*, 13458–13471. https://doi.org/10.1523/JNEUROSCI.2768-14.2014.
6. Gale, S.D., and Murphy, G.J. (2016). Active Dendritic Properties and Local Inhibitory Input Enable Selectivity for Object Motion in Mouse Superior Colliculus Neurons. J. Neurosci. *36*, 9111–9123. https://doi.org/10.1523/JNEUROSCI.0645-16.2016.
7. Chen, C., Liu, Y., and Cang, J. (2025). Accessing genetically defined cell types in the superior colliculus with transgenic mouse lines. iScience *28*, 112194. https://doi.org/10.1016/j.isci.2025.112194.
8. Hoy, J.L., Bishop, H.I., and Niell, C.M. (2019). Defined Cell Types in Superior Colliculus Make Distinct Contributions to Prey Capture Behavior in the Mouse. Curr. Biol. *29*, 4130–4138.e5. https://doi.org/10.1016/j.cub.2019.10.017.
9. Tsai, N.Y., Wang, F., Toma, K., Yin, C., Takatoh, J., Pai, E.L., Wu, K., Matcham, A.C., Yin, L., Dang, E.J., et al. (2022). Trans-Seq maps a selective mammalian retinotectal synapse instructed by Nephronectin. Nat. Neurosci. *25*, 659–674. https://doi.org/10.1038/s41593-022-01068-8.
10. Relota, X.J., Ford, A., and Savier, E.L. (2025). Behavioral Modulation and Molecular Definition of Wide-Field Vertical Cells in the Mouse Superior Colliculus. J. Neurosci. *45*, e1816242025. https://doi.org/10.1523/JNEUROSCI.1816-24.2025.
11. Oliveira, A.F., and Yonehara, K. (2018). The Mouse Superior Colliculus as a Model System for Investigating Cell Type-Based Mechanisms of Visual Motor Transformation. Front. Neural Circuits *12*, 59. https://doi.org/10.3389/fncir.2018.00059.
12. Li, C., Kühn, N.K., Alkislar, I., Sans-Dublanc, A., Zemmouri, F., Paesmans, S., Calzoni, A., Ooms, F., Reinhard, K., and Farrow, K. (2023). Pathway-specific inputs to the superior colliculus support flexible responses to visual threat. Sci. Adv. *9*. https://doi.org/10.1126/sciadv.ade3874.
13. Cao, W., Tan, Z., Berackey, B.T., Nguyen, J.K., Brown, S.R., Du, S., Lin, B., Ye, Q., Seiler, M., Holmes, T.C., and Xu, X. (2025). An AAV capsid proposed as microglia-targeting directs genetic expression in forebrain excitatory neurons. Cell Rep. Methods *5*, 101054. https://doi.org/10.1016/j.crmeth.2025.101054.
14. Goertsen, D., Flytzanis, N.C., Goeden, N., Chuapoco, M.R., Cummins, A., Chen, Y., Fan, Y., Zhang, Q., Sharma, J., Duan, Y., et al. (2022). AAV capsid variants with brain-wide transgene expression and decreased liver targeting after intravenous delivery in mouse and marmoset. Nat. Neurosci. *25*, 106–115. https://doi.org/10.1038/s41593-021-00969-4.
15. Graybuck, L.T., Daigle, T.L., Sedeño-Cortés, A.E., Walker, M., Kalmbach, B., Lenz, G.H., Morin, E., Nguyen, T.N., Garren, E., Bendrick, J.L., et al. (2021). Enhancer viruses for combinatorial cell-subclass-specific labeling. Neuron *109*, 1449–1464.e13. https://doi.org/10.1016/j.neuron.2021.03.011.

16. Mich, J.K., Graybuck, L.T., Hess, E.E., Mahoney, J.T., Kojima, Y., Ding, Y., Somasundaram, S., Miller, J.A., Kalmbach, B.E., Radaelli, C., et al. (2021). Functional enhancer elements drive subclass-selective expression from mouse to primate neocortex. Cell Rep. *34*, 108754. https://doi.org/10.1016/j.celrep.2021.108754.

17. Vormstein-Schneider, D., Lin, J.D., Pelkey, K.A., Chittajallu, R., Guo, B., Arias-Garcia, M.A., Allaway, K., Sakopoulos, S., Schneider, G., Stevenson, O., et al. (2020). Viral manipulation of functionally distinct interneurons in mice, non-human primates and humans. Nat. Neurosci. *23*, 1629–1636. https://doi.org/10.1038/s41593-020-0692-9.

18. Hunker, A.C., Wirthlin, M.E., Gill, G., Johansen, N.J., Hooper, M., Omstead, V., Vargas, S., Lerma, M.N., Taskin, N., Weed, N., et al. (2025). Enhancer AAV toolbox for accessing and perturbing striatal cell types and circuits. Neuron *113*, 1507–1524.e17. https://doi.org/10.1016/j.neuron.2025.04.035.

19. Furlanis, E., Dai, M., Leyva Garcia, B., Tran, T., Vergara, J., Pereira, A., Gorissen, B.L., Wills, S., Vlachos, A., Hairston, A., et al. (2025). An enhancer-AAV toolbox to target and manipulate distinct interneuron subtypes. Neuron *113*, 1525–1547.e15. https://doi.org/10.1016/j.neuron.2025.05.002.

20. Ben-Simon, Y., Hooper, M., Narayan, S., Daigle, T.L., Dwivedi, D., Way, S.W., Oster, A., Stafford, D.A., Mich, J.K., Taormina, M.J., et al. (2025). A suite of enhancer AAVs and transgenic mouse lines for genetic access to cortical cell types. Cell *188*, 3045–3064.e23. https://doi.org/10.1016/j.cell.2025.05.002.

21. Petry, H.M., and Bickford, M.E. (2019). The Second Visual System of The Tree Shrew. J. Comp. Neurol. *527*, 679–693. https://doi.org/10.1002/cne.24413.

22. Van Hooser, S.D., Roy, A., Rhodes, H.J., Culp, J.H., and Fitzpatrick, D. (2013). Transformation of Receptive Field Properties from Lateral Geniculate Nucleus to Superficial V1 in the Tree Shrew. J. Neurosci. *33*, 11494–11505. https://doi.org/10.1523/jneurosci.1464-13.2013.

23. Familtsev, D., Quiggins, R., Masterson, S.P., Dang, W., Slusarczyk, A.S., Petry, H.M., and Bickford, M.E. (2016). Ultrastructure of geniculocortical synaptic connections in the tree shrew striate cortex. J. Comp. Neurol. *524*, 1292–1306. https://doi.org/10.1002/cne.23907.

24. Sciaccotta, F., Kipcak, A., and Erisir, A. (2025). Morphological and Molecular Distinctions of Parallel Processing Streams Reveal Two Koniocellular Pathways in the Tree Shrew dLGN. eNeuro *12*. https://doi.org/10.1523/eneuro.0522-24.2025.

25. Campbell, C.B., Jane, J.A., and Yashon, D. (1967). The retinal projections of the tree shrew and hedgehog. Brain Res. *5*, 406–418. https://doi.org/10.1016/0006-8993(67)90047-9.

26. Albano, J.E., Norton, T.T., and Hall, W.C. (1979). Laminar origin of projections from the superficial layers of the superior colliculus in the tree shrew, Tupaia glis. Brain Res. *173*, 1–11. https://doi.org/10.1016/0006-8993(79)91090-4.

27. Chomsung, R.D., Wei, H., Day-Brown, J.D., Petry, H.M., and Bickford, M.E. (2010). Synaptic Organization of Connections between the Temporal Cortex and Pulvinar Nucleus of the Tree Shrew. Cereb. Cortex *20*, 997–1011. https://doi.org/10.1093/cercor/bhp162.

28. Wei, H., Masterson, S.P., Petry, H.M., and Bickford, M.E. (2011). Diffuse and Specific Tectopulvinar Terminals in the Tree Shrew: Synapses, Synapsins, and Synaptic Potentials. PLoS One *6*, e23781. https://doi.org/10.1371/journal.pone.0023781.

29. Gaillard, F., and Galand, G. (1979). Diencephalic binocular wide field neurons in the frog. Exp. Brain Res. *34*, 511–520. https://doi.org/10.1007/bf00239146.

30. Kang, H.-J., and Li, X.-H. (2010). Response properties and receptive field organization of collision-sensitive neurons in the optic tectum of bullfrog, Rana catesbeiana. Neurosci. Bull. *26*, 304–316. https://doi.org/10.1007/s12264-010-0306-8.

31. Karten, H.J., Cox, K., and Mpodozis, J. (1997). Two distinct populations of tectal neurons have unique connections within the retinotectorotundal pathway of the pigeon (Columba livia). J. Comp. Neurol. *387*, 449–465.

32. Luksch, H., Cox, K., and Karten, H.J. (1998). Bottlebrush dendritic endings and large dendritic fields: motion-detecting neurons in the tectofugal pathway. J. Comp. Neurol. *396*, 399–414.

33. Luppino, G., Matelli, M., Carey, R.G., Fitzpatrick, D., and Diamond, I.T. (1988). New view of the organization of the pulvinar nucleus in Tupaia as revealed by tectopulvinar and pulvinar-cortical projections. J. Comp. Neurol. *273*, 67–86. https://doi.org/10.1002/cne.902730107.

34. Hilbig, H., Merbach, M., Krause, J., Gärtner, U., and Stubbe, A. (2000). Dendritic organization of neurons of the superior colliculus in animals with different visual capability. Brain Res. Bull. *51*, 255–265. https://doi.org/10.1016/S0361-9230(99)00230-0.

35. Chomsung, R.D., Petry, H.M., and Bickford, M.E. (2008). Ultrastructural examination of diffuse and specific tectopulvinar projections in the tree shrew. J. Comp. Neurol. *510*, 24–46. https://doi.org/10.1002/cne.21763.

36. Baldwin, M.K.L., Balaram, P., and Kaas, J.H. (2013). Projections of the superior colliculus to the pulvinar in prosimian galagos (*Otolemur garnettii*) and VGLUT2 staining of the visual pulvinar. J. Comp. Neurol. *521*, 1664–1682. https://doi.org/10.1002/cne.23252.

37. Harting, J.K., Huerta, M.F., Frankfurter, A.J., Strominger, N.L., and Royce, G.J. (1980). Ascending pathways from the monkey superior colliculus: An autoradiographic analysis. J. Comp. Neurol. *192*, 853–882. https://doi.org/10.1002/cne.901920414.

38. Benevento, L.A., and Standage, G.P. (1983). The organization of projections of the retinorecipient and nonretinorecipient nuclei of the pretectal complex and layers of the superior colliculus to the lateral pulvinar and medial pulvinar in the macaque monkey. J. Comp. Neurol. *217*, 307–336. https://doi.org/10.1002/cne.902170307.

39. Huerta, M.F., and Harting, J.K. (1983). Sublamination within the superficial gray layer of the squirrel monkey: an analysis of the tectopulvinar projection using anterograde and retrograde transport methods. Brain Res. *261*, 119–126. https://doi.org/10.1016/0006-8993(83)91290-8.

40. Stepniewska, I., QI, H.X., and Kaas, J.H. (2000). Projections of the superior colliculus to subdivisions of the inferior pulvinar in New World and Old World monkeys. Vis. Neurosci. *17*, 529–549. https://doi.org/10.1017/s0952523800174048.

41. Lyon, D.C., Nassi, J.J., and Callaway, E.M. (2010). A Disynaptic Relay from Superior Colliculus to Dorsal Stream Visual Cortex in Macaque Monkey. Neuron *65*, 270–279. https://doi.org/10.1016/j.neuron.2010.01.003.

42. Elorette, C., Forcelli, P.A., Saunders, R.C., and Malkova, L. (2018). Colocalization of Tectal Inputs With Amygdala-Projecting Neurons in the Macaque Pulvinar. Front. Neural Circuits *12*, 91. https://doi.org/10.3389/fncir.2018.00091.

43. Kühn, N.K., Li, C., Baimacheva, N., Zimmer, J., Reinhard, K., Bonin, V., and Farrow, K. (2025). Dendritic architecture enables *de novo* computation of salient motion in the superior colliculus. Curr. Biol. *S0960982225008206*. https://doi.org/10.1016/j.cub.2025.06.060.

44. Shang, C., Chen, Z., Liu, A., Li, Y., Zhang, J., Qu, B., Yan, F., Zhang, Y., Liu, W., Liu, Z., et al. (2018). Divergent midbrain circuits orchestrate escape and freezing responses to looming stimuli in mice. Nat. Commun. *9*, 1232. https://doi.org/10.1038/s41467-018-03580-7.

45. Shang, C., Liu, Z., Chen, Z., Shi, Y., Wang, Q., Liu, S., Li, D., and Cao, P. (2015). A parvalbumin-positive excitatory visual pathway to trigger fear responses in mice. Science *348*, 1472–1477. https://doi.org/10.1126/science.aaa8694.

46. Reinhard, K., Li, C., Do, Q., Burke, E.G., Heyndericks, S., and Farrow, K. (2019). A projection specific logic to sampling visual inputs in mouse superior colliculus. eLife *8*, e50697. https://doi.org/10.7554/elife.50697.

47. Zingg, B., Chou, X.L., Zhang, Z.G., Mesik, L., Liang, F., Tao, H.W., and Zhang, L.I. (2017). AAV-Mediated Anterograde Transsynaptic Tagging: Mapping Corticocollicular Input-Defined Neural Pathways for Defense

Behaviors. Neuron *93*, 33–47. https://doi.org/10.1016/j.neuron.2016.11.045.

48. Wei, P., Liu, N., Zhang, Z., Liu, X., Tang, Y., He, X., Wu, B., Zhou, Z., Liu, Y., Li, J., et al. (2015). Processing of visually evoked innate fear by a non-canonical thalamic pathway. Nat. Commun. *6*, 6756. https://doi.org/10.1038/ncomms7756.
49. Ayzenberg, V., Song, C., and Arcaro, M.J. (2025). An intrinsic hierarchical, retinotopic organization of visual pulvinar connectivity in the human neonate. Curr. Biol. *35*, 300–314.e5. https://doi.org/10.1016/j.cub.2024.11.042.
50. Arcaro, M.J., Pinsk, M.A., and Kastner, S. (2015). The Anatomical and Functional Organization of the Human Visual Pulvinar. J. Neurosci. *35*, 9848–9871. https://doi.org/10.1523/jneurosci.1575-14.2015.
51. Baldwin, M.K.L., Balaram, P., and Kaas, J.H. (2017). The evolution and functions of nuclei of the visual pulvinar in primates. J. Comp. Neurol. *525*, 3207–3226. https://doi.org/10.1002/cne.24272.
52. Leow, Y.N., Zhou, B., Sullivan, H.A., Barlowe, A.R., Wickersham, I.R., and Sur, M. (2022). Brain-wide mapping of inputs to the mouse lateral posterior (LP/Pulvinar) thalamus–anterior cingulate cortex network. J. Comp. Neurol. *530*, 1992–2013. https://doi.org/10.1002/cne.25317.
53. Norton, T.T., Savier, E.L., and Sedigh-Sarvestani, M. (2022). DeBruyn and Casagrande manuscripts on tree shrew retinal ganglion cells as a basis for cross-species retina research. Vis. Neurosci. *39*, E001. https://doi.org/10.1017/S0952523821000171.
54. Savier, E., Sedigh-Sarvestani, M., Wimmer, R., and Fitzpatrick, D. (2021). A bright future for the tree shrew in neuroscience research: Summary from the inaugural Tree Shrew Users Meeting. Zool. Res. *42*, 478–481. https://doi.org/10.24272/j.issn.2095-8137.2021.178.
55. Liu, Y., McDaniel, J.A., Chen, C., Yang, L., Kipcak, A., Savier, E.L., Erisir, A., Cang, J., and Campbell, J.N. (2025). Co-Conservation of synaptic gene expression and circuitry in collicular neurons. Nat. Commun. *16*, 9146. https://doi.org/10.1038/s41467-025-64204-5.
56. Masterson, S.P., Li, J., and Bickford, M.E. (2010). Frequency-Dependent Release of Substance P Mediates Heterosynaptic Potentiation of Glutamatergic Synaptic Responses in the Rat Visual Thalamus. J. Neurophysiol. *104*, 1758–1767. https://doi.org/10.1152/jn.00010.2010.
57. Zhou, N.A., Maire, P.S., Masterson, S.P., and Bickford, M.E. (2017). The mouse pulvinar nucleus: Organization of the tectorecipient zones. Vis. Neurosci. *34*, E011. https://doi.org/10.1017/S0952523817000050.
58. Xie, Z., Wang, M., Liu, Z., Shang, C., Zhang, C., Sun, L., Gu, H., Ran, G., Pei, Q., Ma, Q., et al. (2021). Transcriptomic encoding of sensorimotor transformation in the midbrain. eLife *10*, e69825. https://doi.org/10.7554/eLife.69825.
59. Matcham, A.C., Toma, K., Tsai, N.Y., Sze, C.J., Lin, P.-Y., Stewart, I.F., and Duan, X. (2024). Cadherin-13 Maintains Retinotectal Synapses via Transneuronal Interactions. J. Neurosci. *44*, e1310232023. https://doi.org/10.1523/JNEUROSCI.1310-23.2023.
60. Zhu, Y., Sun, L., Chen, Z., Whitaker, J.W., Wang, T., and Wang, W. (2013). Predicting enhancer transcription and activity from chromatin modifications. Nucleic Acids Res. *41*, 10032–10043. https://doi.org/10.1093/nar/gkt826.
61. Crawford, G.E., Holt, I.E., Whittle, J., Webb, B.D., Tai, D., Davis, S., Margulies, E.H., Chen, Y., Bernat, J.A., Ginsburg, D., et al. (2006). Genome-wide mapping of DNase hypersensitive sites using massively parallel signature sequencing (MPSS). Genome Res. *16*, 123–131. https://doi.org/10.1101/gr.4074106.
62. Rajagopal, N., Xie, W., Li, Y., Wagner, U., Wang, W., Stamatoyannopoulos, J., Ernst, J., Kellis, M., and Ren, B. (2013). RFECS: A Random-Forest Based Algorithm for Enhancer Identification from Chromatin State. PLoS Comput. Biol. *9*, e1002968. https://doi.org/10.1371/journal.pcbi.1002968.
63. Wu, Z., Yang, H., and Colosi, P. (2010). Effect of Genome Size on AAV Vector Packaging. Mol. Ther. *18*, 80–86. https://doi.org/10.1038/mt.2009.255.
64. Gadella, T.W.J., Van Weeren, L., Stouthamer, J., Hink, M.A., Wolters, A.H.G., Giepmans, B.N.G., Aumonier, S., Dupuy, J., and Royant, A. (2023). mScarlet3: a brilliant and fast-maturing red fluorescent protein. Nat. Methods *20*, 541–545. https://doi.org/10.1038/s41592-023-01809-y.
65. Song, A.J., and Palmiter, R.D. (2018). Detecting and Avoiding Problems When Using the Cre–lox System. Trends Genet. *34*, 333–340. https://doi.org/10.1016/j.tig.2017.12.008.
66. Fischer, K.B., Collins, H.K., and Callaway, E.M. (2019). Sources of off-target expression from recombinase-dependent AAV vectors and mitigation with cross-over insensitive ATG-out vectors. Proc. Natl. Acad. Sci. *116*, 27001–27010. https://doi.org/10.1073/pnas.1915974116.
67. Matsushita, N., Kato, S., Nishizawa, K., Sugawara, M., Takeuchi, K., Miyasaka, Y., Mashimo, T., and Kobayashi, K. (2023). Highly selective transgene expression through the flip-excision switch system by using a unilateral spacer sequence. Cell Rep. Methods *3*, 100393. https://doi.org/10.1016/j.crmeth.2022.100393.
68. Moradian, H., Gossen, M., and Lendlein, A. (2022). Co-delivery of genes can be confounded by bicistronic vector design. MRS Commun. *12*, 145–153. https://doi.org/10.1557/s43579-021-00128-7.
69. Liu, Z., Chen, O., Wall, J.B.J., Zheng, M., Zhou, Y., Wang, L., Vaseghi, H.R., Qian, L., and Liu, J. (2017). Systematic comparison of 2A peptides for cloning multi-genes in a polycistronic vector. Sci. Rep. *7*, 2193. https://doi.org/10.1038/s41598-017-02460-2.
70. Tigges, J. (1966). Ein Experimenteller Beitrag Zum Subkortikalen Optischen System Von Tupaia Glis. Folia Primatol. *4*, 103–123. https://doi.org/10.1159/000155047.
71. Lee, P., and Hall, W.C. (1995). Interlaminar connections of the superior colliculus in the tree shrew. II: Projections from the superficial gray to the optic layer. Vis. Neurosci. *12*, 573–588. https://doi.org/10.1017/S0952523800008464.
72. Baldwin, M.K.L., Wei, H., Reed, J.L., Bickford, M.E., Petry, H.M., and Kaas, J.H. (2013). Cortical projections to the superior colliculus in tree shrews (Tupaia belangeri). J. Comp. Neurol. *521*, 1614–1632. https://doi.org/10.1002/cne.23249.
73. Masterson, S.P., Zhou, N., Akers, B.K., Dang, W., and Bickford, M.E. (2019). Ultrastructural and optogenetic dissection of V1 corticotectal terminal synaptic properties. J. Comp. Neurol. *527*, 833–842. https://doi.org/10.1002/cne.24538.
74. Jiang, S., Honnuraiah, S., and Stuart, G.J. (2023). Characterization of primary visual cortex input to specific cell types in the superior colliculus. Front. Neuroanat. *17*, 1282941. https://doi.org/10.3389/fnana.2023.1282941.
75. Su, J., Sabbagh, U., Liang, Y., Olejníková, L., Dixon, K.G., Russell, A.L., Chen, J., Pan, Y.A., Triplett, J.W., and Fox, M.A. (2021). A cell–ECM mechanism for connecting the ipsilateral eye to the brain. Proc. Natl. Acad. Sci. *118*, e2104343118. https://doi.org/10.1073/pnas.2104343118.
76. Balaram, P., Isaamullah, M., Petry, H.M., Bickford, M.E., and Kaas, J.H. (2015). Distributions of vesicular glutamate transporters 1 and 2 in the visual system of tree shrews (*Tupaia belangeri*). J. Comp. Neurol. *523*, 1792–1808. https://doi.org/10.1002/cne.23727.
77. Cheung, V., Chung, P., Bjorni, M., Shvareva, V.A., Lopez, Y.C., and Feinberg, E.H. (2021). Virally encoded connectivity transgenic overlay RNA sequencing (VECTORseq) defines projection neurons involved in sensorimotor integration. Cell Rep. *37*, 110131. https://doi.org/10.1016/j.celrep.2021.110131.
78. Südhof, T.C. (2023). Cerebellin–neurexin complexes instructing synapse properties. Curr. Opin. Neurobiol. *81*, 102727. https://doi.org/10.1016/j.conb.2023.102727.
79. Dai, J., Liakath-Ali, K., Golf, S.R., and Südhof, T.C. (2022). Distinct neurexin-cerebellin complexes control AMPA- and NMDA-receptor responses in a circuit-dependent manner. eLife *11*, e78649. https://doi.org/10.7554/elife.78649.

80. Seigneur, E., Wang, J., Dai, J., Polepalli, J., and Südhof, T.C. (2021). Cerebellin-2 regulates a serotonergic dorsal raphe circuit that controls compulsive behaviors. Mol. Psychiatr. *26*, 7509–7521. https://doi.org/10.1038/s41380-021-01187-x.

81. Seigneur, E., and Südhof, T.C. (2017). Cerebellins are differentially expressed in selective subsets of neurons throughout the brain. J. Comp. Neurol. *525*, 3286–3311. https://doi.org/10.1002/cne.24278.

82. Seigneur, E., and Südhof, T.C. (2018). Genetic Ablation of All Cerebellins Reveals Synapse Organizer Functions in Multiple Regions Throughout the Brain. J. Neurosci. *38*, 4774–4790. https://doi.org/10.1523/jneurosci.0360-18.2018.

83. Shibata, M., Pattabiraman, K., Muchnik, S.K., Kaur, N., Morozov, Y.M., Cheng, X., Waxman, S.G., and Sestan, N. (2021). Hominini-specific regulation of CBLN2 increases prefrontal spinogenesis. Nature *598*, 489–494. https://doi.org/10.1038/s41586-021-03952-y.

84. Martersteck, E.M., Hirokawa, K.E., Evarts, M., Bernard, A., Duan, X., Li, Y., Ng, L., Oh, S.W., Ouellette, B., Royall, J.J., et al. (2017). Diverse Central Projection Patterns of Retinal Ganglion Cells. Cell Rep. *18*, 2058–2072. https://doi.org/10.1016/j.celrep.2017.01.075.

85. Day-Brown, J.D., Wei, H., Chomsung, R.D., Petry, H.M., and Bickford, M.E. (2010). Pulvinar Projections to the Striatum and Amygdala in the Tree Shrew. Front. Neuroanat. *4*, 143. https://doi.org/10.3389/fnana.2010.00143.

86. Fredes, F., Vega-Zuniga, T., Karten, H., and Mpodozis, J. (2012). Bilateral and ipsilateral ascending tectopulvinar pathways in mammals: A study in the squirrel (spermophilus beecheyi). J. Comp. Neurol. *520*, 1800–1818. https://doi.org/10.1002/cne.23014.

87. Benevento, L.A., and Fallon, J.H. (1975). The ascending projections of the superior colliculus in the rhesus monkey (Macaca mulatta). J. Comp. Neurol. *160*, 339–361. https://doi.org/10.1002/cne.901600306.

88. Benevento, L.A., and Rezak, M. (1976). The cortical projections of the inferior pulvinar and adjacent lateral pulvinar in the rhesus monkey (Macaca mulatta): an autoradiographic study. Brain Res. *108*, 1–24. https://doi.org/10.1016/0006-8993(76)90160-8.

89. Davidson, R.M., Joly, T.J., and Bender, D.B. (1992). Effect of corticotectal tract lesions on relative motion selectivity in the monkey superior colliculus. Exp. Brain Res. *92*, 246–258. https://doi.org/10.1007/BF00227968.

90. Fan, Y., Huang, Z.-Y., Cao, C.-C., Chen, C.-S., Chen, Y.-X., Fan, D.-D., He, J., Hou, H.-L., Hu, L., Hu, X.-T., et al. (2013). Genome of the Chinese tree shrew. Nat. Commun. *4*, 1426. https://doi.org/10.1038/ncomms2416.

91. Ye, M.-S., Zhang, J.-Y., Yu, D.-D., Xu, M., Xu, L., Lv, L.-B., Zhu, Q.-Y., Fan, Y., and Yao, Y.-G. (2021). Comprehensive annotation of the Chinese tree shrew genome by large-scale RNA sequencing and long-read isoform sequencing. Zool. Res. *42*, 692–709. https://doi.org/10.24272/j.issn.2095-8137.2021.272.

92. Xu, L., Chen, S.-Y., Nie, W.-H., Jiang, X.-L., and Yao, Y.-G. (2012). Evaluating the Phylogenetic Position of Chinese Tree Shrew (Tupaia belangeri chinensis) Based on Complete Mitochondrial Genome: Implication for Using Tree Shrew as an Alternative Experimental Animal to Primates in Biomedical Research. J. Genet. Genomics *39*, 131–137. https://doi.org/10.1016/j.jgg.2012.02.003.

93. Yao, Y.-G., Lu, L., Ni, R.-J., Bi, R., Chen, C., Chen, J.-Q., Fuchs, E., Gorbatyuk, M., Lei, H., Li, H., et al. (2024). Study of tree shrew biology and models: A booming and prosperous field for biomedical research. Zool. Res. *45*, 877–909. https://doi.org/10.24272/j.issn.2095-8137.2024.199.

94. Chen, L., Fish, A.E., and Capra, J.A. (2018). Prediction of gene regulatory enhancers across species reveals evolutionarily conserved sequence properties. PLoS Comput. Biol. *14*, e1006484. https://doi.org/10.1371/journal.pcbi.1006484.

95. Laverré, A., Tannier, E., and Necsulea, A. (2022). Long-range promoter-enhancer contacts are conserved during evolution and contribute to gene expression robustness. Genome Res. *32*, 280–296. https://doi.org/10.1101/gr.275901.121.

96. Villar, D., Berthelot, C., Aldridge, S., Rayner, T.F., Lukk, M., Pignatelli, M., Park, T.J., Deaville, R., Erichsen, J.T., Jasinska, A.J., et al. (2015). Enhancer evolution across 20 mammalian species. Cell *160*, 554–566. https://doi.org/10.1016/j.cell.2015.01.006.

97. Lizio, M., Harshbarger, J., Shimoji, H., Severin, J., Kasukawa, T., Sahin, S., Abugessaisa, I., Fukuda, S., Hori, F., Ishikawa-Kato, S., et al. (2015). Gateways to the FANTOM5 promoter level mammalian expression atlas. Genome Biol. *16*, 22. https://doi.org/10.1186/s13059-014-0560-6.

98. Fullard, J.F., Hauberg, M.E., Bendl, J., Egervari, G., Cirnaru, M.-D., Reach, S.M., Motl, J., Ehrlich, M.E., Hurd, Y.L., and Roussos, P. (2018). An atlas of chromatin accessibility in the adult human brain. Genome Res. *28*, 1243–1252. https://doi.org/10.1101/gr.232488.117.

99. Schindelin, J., Arganda-Carreras, I., Frise, E., Kaynig, V., Longair, M., Pietzsch, T., Preibisch, S., Rueden, C., Saalfeld, S., Schmid, B., et al. (2012). Fiji: an open-source platform for biological-image analysis. Nat. Methods *9*, 676–682. https://doi.org/10.1038/nmeth.2019.

100. Arshadi, C., Günther, U., Eddison, M., Harrington, K.I.S., and Ferreira, T.A. (2021). SNT: a unifying toolbox for quantification of neuronal anatomy. Nat. Methods *18*, 374–377. https://doi.org/10.1038/s41592-021-01105-7.

101. Gearing, L.J., Cumming, H.E., Chapman, R., Finkel, A.M., Woodhouse, I.B., Luu, K., Gould, J.A., Forster, S.C., and Hertzog, P.J. (2019). CiiiDER: A tool for predicting and analysing transcription factor binding sites. PLoS One *14*, e0215495. https://doi.org/10.1371/journal.pone.0215495.

102. Perez, G., Barber, G.P., Benet-Pages, A., Casper, J., Clawson, H., Diekhans, M., Fischer, C., Gonzalez, J.N., Hinrichs, A.S., Lee, C.M., et al. (2025). The UCSC Genome Browser database: 2025 update. Nucleic Acids Res. *53*, D1243–D1249. https://doi.org/10.1093/nar/gkae974.

103. Luo, Y., Hitz, B.C., Gabdank, I., Hilton, J.A., Kagda, M.S., Lam, B., Myers, Z., Sud, P., Jou, J., Lin, K., et al. (2020). New developments on the Encyclopedia of DNA Elements (ENCODE) data portal. Nucleic Acids Res. *48*, D882–D889. https://doi.org/10.1093/nar/gkz1062.

104. R Core Team (2021). R: A language and environment for statistical computing (Vienna, Austria: R Foundation for Statistical computing). https://www.R-project.org/.

105. Posit (2025). RStudio: Integrated Development for R (Boston, MA: Posit Software, PBC). http://www.posit.co/.

106. Bodenhofer, U., Bonatesta, E., Horejš-Kainrath, C., and Hochreiter, S. (2015). msa: an R package for multiple sequence alignment. Bioinformatics *31*, 3997–3999. https://doi.org/10.1093/bioinformatics/btv494.

107. Pagès H., Aboyoun P., Gentleman R., DebRoy S. (2024) Biostrings: Efficient manipulation of biological strings. R package version 2.72.1. doi: 10.18129/B9.bioc.Biostrings

108. Wickham, H., François, R., Henry, L., Müller, K., and Vaughan, D. (2025). dplyr: A Grammar of Data Manipulation. R package version 1.1.4.

109. Müller, K., and Wickham, H. (2025). tibble: Simple Data Frames. R package version 3.3.0.

110. Wickham, H., Hester, J., and Bryan, J. (2024). readr: Read Rectangular Text Data. R package version 2.1.5.

111. Wickham, H. (2023). stringr: Simple, Consistent Wrappers for Common String Operations. R package version 1.5.1.

112. Wickham, H. (2016). ggplot2: Elegant Graphics for Data Analysis (New York: Springer-Verlag).

113. Wickham, H., Vaughan, D., and Girlich, M. (2025). tidyr: Tidy Messy Data. R package version 1.3.1.

114. Murrell, P. (2005). The Grid Graphics Package (Chapman & Hall/CRC Press).

115. Albano, J.E., Humphrey, A.L., and Norton, T.T. (1978). Laminar organization of receptive-field properties in tree shrew superior colliculus. J. Neurophysiol. *41*, 1140–1164. https://doi.org/10.1152/jn.1978.41.5.1140.

116. Corson, J.A., and Erisir, A. (2013). Monosynaptic convergence of chorda tympani and glossopharyngeal afferents onto ascending relay neurons in the nucleus of the solitary tract: a high-resolution confocal and correlative electron microscopy approach. J. Comp. Neurol. *521*, 2907–2926. https://doi.org/10.1002/cne.23357.

117. Balcioglu, A., Gillani, R., Doron, M., Burnell, K., Ku, T., Erisir, A., Chung, K., Segev, I., and Nedivi, E. (2023). Mapping thalamic innervation to individual L2/3 pyramidal neurons and modeling their 'readout' of visual input. Nat. Neurosci. *26*, 470–480. https://doi.org/10.1038/s41593-022-01253-9.

118. Akter, Y., Jones, G., Daskivich, G.J., Shifflett, V., Vargas, K.J., and Hruska, M. (2025). Combining nanobody labeling with STED microscopy reveals input-specific and layer-specific organization of neocortical synapses. PLoS Biol. *23*, e3002649. https://doi.org/10.1371/journal.pbio.3002649.

STAR★METHODS

KEY RESOURCES TABLE

REAGENT or RESOURCE	SOURCE	IDENTIFIER
Antibodies		
Rabbit Anti-NPNT Antibody (1:500)	Novus	Cat# NBP1-83990
Rabbit Anti-CBLN2 Antibody (1:500)	Invitrogen	Cat# PA5-101514; RRID: AB_2850949
Rabbit Anti-Cre (1:1000)	Sigma-Aldrich	Cat# 69050
Rabbit Anti-GFP (1:1000)	Sigma-Aldrich	Cat# AB3080
Rabbit Anti-GABA (1:250)	Sigma-Aldrich	Cat# A2052
Mouse Anti-PV (1:200)	Sigma-Aldrich	Cat# P3088
Guinea Pig Anti-VGluT2 (1:1000)	Sigma-Aldrich	Cat# AB2251-I
Mouse Anti-VGluT2 (1:1000)	Chemicon	Cat# MAB5504
Mouse Anti-VGluT1 (1:1000)	Chemicon	Cat# MAB5502
Rabbit Anti-VGluT1 (1:1000)	Synaptic Systems	Cat# 135 303
Rabbit Anti-PSD95 (1:100)	Sigma-Aldrich	Cat# S1-6900
Rabbit Anti-SP (1:1000)	Chemicon	Cat# AB1566
Goat Anti-ChAT (1:100)	Chemicon	Cat# AB144P
Rabbit Anti-CALB2 (1:250)	Swant	Cat# CR7697
Donkey Anti-Goat Cy3	Jackson ImmunoResearch	Cat# 705-165-003 RRID: AB_2340411
Goat Anti-Guinea Pig AF488 (1:250)	Abcam	Cat# ab150185
Donkey Anti-Guinea Pig Cy5 (1:250)	Jackson ImmunoResearch	Cat# 706-175- 14; RRID: AB_2340462
Goat Anti-Guinea Pig AF594 (1:250)	Invitrogen	Cat# A-11076
Donkey Anti-Rabbit AF555 (1:250)	Invitrogen	Cat# A-31572
Donkey Anti-Rabbit AF488 (1:250)	Invitrogen	Cat# A-21206; RRID: AB_2535792
Donkey Anti-Rabbit Cy5 (1:250)	Jackson ImmunoResearch	Cat# 711-175-152 RRID: AB_2340607
Donkey Anti-Mouse TRITC (1:250)	Jackson ImmunoResearch	Cat# 715-025-151 RRID: AB_2340767
Donkey Anti-Mouse AF647 (1:250)	Invitrogen	Cat# A-31571
Donkey Anti-Mouse AF488 (1:250)	Invitrogen	Cat# A-21202 RRID: AB_141607
Goat Anti-Rabbit Biotinylated (1:200)	Vector Laboratories	Cat# BA-1000
Bacterial and virus strains		
AAV1-tsC2Pro-GFP	This paper	Addgene, Cat# 248521
AAV9-tsC2ProT-mScarlet3-Cre	This paper	Addgene, Cat# 248522
AAV1-Ef1a-DIO-EYFP	Karl Deisseroth Lab	Addgene, Cat# 27056
AAV9-CAG-FLEX-tdTomato	Edward Boyden Lab	Addgene, Cat# 28306
AAVrg-CAG-GFP	Edward Boyden Lab	Addgene, Cat# 37825
Chemicals, peptides, and recombinant proteins		
BSA	Thermo Scientific	Cat# AAJ6410036
Triton X-100	MP Biomedicals	Cat# 194854
Sodium Azide	MP Biomedicals	Cat# 102891
Glycerol	Mallinckrodt	N/A
Ethylene Glycol	Supelco	Cat# EX0565

(Continued on next page)

Continued		
REAGENT or RESOURCE	SOURCE	IDENTIFIER
NeuroTrace Nissl Stain	Invitrogen	Cat# N21479
TSA Vivid Fluorophore 650 (1:1000)	ACD	Cat# 323273
Vectashield Plus DAPI	Vector Laboratories	Cat# H-2000
Critical commercial assays		
RNAscope^{TM} Multiplex v2	ACD	Cat# 323270
VECTASTAIN® ABC-HRP Kit, Peroxidase	Vector Laboratories	Cat# PK-4000
Deposited data		
TF binding motif data	This paper	Data S4
AAV vector maps and sequences	This paper	Data S3
Cross-species CBLN2 promoter sequence annotations	This paper	Data S2
Cross-species CBLN2 CDS annotations	This paper	Data S1
FANTOM5 TSS data	Lizio et al.[97]	https://genome.ucsc.edu/cgi-bin/hgTrackUi?db=hg38&g=fantom5
BOCA ATAC-Seq data	Fullard et al.[98]	https://labs.icahn.mssm.edu/roussos-lab/boca/; GEO: GSE96949
ENCODE DNase-Seq data	John Stamatoyannopoulos	ENCSR880CUB
ENCODE H3K4Me3 ChIP-Seq data	Bradley Bernstein	ENCSR257VEO
ENCODE H3K27Ac ChIP-Seq data	Bradley Bernstein	ENCSR004HIE
Experimental models: Organisms/strains		
T. belangeri Tree Shrew	This paper	UVA Tree shrew Colony
M. musculus Mouse	This paper	C57BL/6J; RRID: IMSR_JAX:000664
Oligonucleotides		
Tree Shrew *CBLN2* FISH Probe	ACD	Cat# 428551
Mouse *CBLN2* FISH Probe	ACD	Cat# 1265181-C1
Recombinant DNA		
AAV-tsC2Pro-GFP	This paper	Addgene, Cat# 248521
AAV-tsC2ProT-mScarlet3-Cre	This paper	Addgene, Cat# 248522
Software and algorithms		
Imaris (10.2.0)	Oxford Instruments	https://imaris.oxinst.com/
ImageJ/FIJI (1.54p)	Schindelin et al.[99]	https://imagej.net/software/fiji/
SNT (4.3.0)	Arshadi et al.[100]	https://imagej.net/plugins/snt/
GraphPad Prism (10.5.0)	Dotmatics	http://www.graphpad.com
SnapGene (8.1.1)	Dotmatics	http://www.snapgene.com
CIIDER	Gearing et al.[101]	https://ciiider.erc.moash.edu
NCBI BLAST	NIH/NLM	https://blast.ncbi.nlm.nih.gov/Blast.cgi
Tree shrew Database	Ye et al.[91]	http://www.treeshrewdb.org/
UCSC Genome Browser	Perez et al.[102]	https://genome.ucsc.edu/
ENCODE Portal	Luo et al.[103]	https://www.encodeproject.org/
R (4.4.1)	R Core Team[104]	https://cran.rstudio.com/
RStudio (2025.05.0 + 496)	Posit team[105]	https://posit.co/download/rstudio-desktop/
msa (R library)	Bodenhofer et al.[106]	https://doi.org/10.18129/B9.bioc.msa
Biostrings (R library)	Pagès et al.[107]	https://doi.org/10.18129/B9.bioc.Biostrings
dplyr (R library)	Wickham et al.[108]	https://dplyr.tidyverse.org/
tibble (R library)	Müller and Wickham[109]	https://tibble.tidyverse.org/
readr (R library)	Wickham et al.[110]	https://readr.tidyverse.org/
stringr (R library)	Wickham[111]	https://stringr.tidyverse.org
ggplot2 (R library)	Wickham[112]	https://ggplot2.tidyverse.org/
tidyr (R library)	Wickham[113]	https://tidyr.tidyverse.org/

(*Continued on next page*)

Continued		
REAGENT or RESOURCE	SOURCE	IDENTIFIER
Grid (R library)	Murrell[114]	https://stat.ethz.ch/R-manual/R-devel/library/grid/html/grid-package.html

EXPERIMENTAL MODEL AND STUDY PARTICIPANT DETAILS

Data for this study was collected from the brains of 10 adult tree shrews (*Tupaia belangeri*) and 6 adult mice. Further Information related to the animals used in this study are provided in the table below. All procedures were approved by the University of Virginia Institutional Animal Care and Use Committee (IACUC) and were conducted in accordance with the National Institutes of Health guidelines.

Species	Animal ID	Sex	Age (months)
T. belangeri	1	M	82
T. belangeri	2	M	35
T. belangeri	3	M	9
T. belangeri	4	F	15
T. belangeri	5	M	19
T. belangeri	6	M	32
T. belangeri	7	F	33
T. belangeri	8	M	10
T. belangeri	9	F	12
T. belangeri	10	F	17
M. musculus	1	M	7
M. musculus	2	M	7
M. musculus	3	M	9
M. musculus	4	M	3
M. musculus	5	M	3
M. musculus	6	M	3

METHOD DETAILS

Stereotaxic surgeries

The animals were anesthetized with 4% isoflurane (SomnoFlo; Kent Scientific, Torrington, CT) and placed on a stereotaxic apparatus. An incision was made along the scalp, and a craniotomy was performed above the SC. The stereotaxic coordinates used for tree shrew SC injections were AP: −6.0-7.5, ML: 2.0–2.5, DV: 3.5–4.0 mm; and for mice SC injections were AP: −3.5–4, ML: 0.6, DV: 1.2 mm. A NanoFil 10 μL syringe equipped with 34g beveled needle (WPI, Sarasota, FL) was used to deliver ≈500nL/site of AAV in saline (see key resources table) at an infusion rate of 1 nL/s using Legato 130 Nanosystem (KD Scientific). The final titers used to dose SC with AAVs were as follows: AAV1-tsC2Pro-GFP (5×10^{11} GC/mL), AAV9-tsC2ProT-mScarlet3-Cre (5×10^{11} GC/mL), AAV1-Ef1A-DIO-EYFP (2.5×10^{12} GC/mL), AAV9-CAG-FLEX-tdTomato (1.2×10^{12} GC/mL), AAVrg-CAG-GFP (2×10^{13} GC/mL). In the experiments where two AAVs were co-injected, the aforementioned final titers were reached through a 1:1 mix of the two AAVs before injection. The needle was left at target for an additional 5 min at the end of the injection cycle to minimize backflow before it was retracted. The craniotomy was sealed with bone wax, the scalp was sutured, and the animals were placed on a heating pad until mobile. After the surgery, animals were monitored for three days to ensure proper wound healing and were observed for any behaviors indicative of pain or discomfort.

AAV design and preparation

Custom AAVs described in the paper were designed using SnapGene software (GSL Biotech, Boston, USA). Borders of the full-length tree shrew promoter (tsC2Pro) were determined based on the sequence conservation to and predicted regulatory elements of the aligned human promoter. Briefly, human CBLN2 locus ±50 kb flanking the first and the last exon (UCSC Genome[102]: hg38,

chr18:72,536,681-72,544,342) was aligned to tree shrew genome (Tree shrew Database, KIZ3[91]) using NCBI Basic Local Alignment Search Tool (BLASTn), which yielded A 3.7kb conserved block in the human locus spanning from 0.3 kb upstream to the first exon to 0.9 kb downstream to the third exon. 5′ end of this sequence was marked as the start of the promoter. Within this block, there was an Open Reading Frame (chr18:72,542,352-72,542,639) in the (+)strand, 0.2 kb upstream to the coding sequence (CDS) of the canonical transcript (NM182511.4), which may interfere with the expression of the reporter to be cloned. For this reason, that region of the block was omitted and the 3′ end of the rest of the blasted region was marked as the end of the promoter. In addition, the incorporated region also neatly aligned with the predicted *cis*-regulatory elements with promoter- and enhancer-like signatures (Figures 1F and 1G). Thus, the coordinates of the human CBLN2 promoter (huC2Pro) were designated as chr18:72,542,815-72,544,612. Finally, this region was aligned to the tree shrew (tsC2Pro: KIZ3 chr12:11145679-1114749) and mouse (mm39 chr18:86728752-86729905) loci reported in the Figure 1. Truncation of the promoter (tsC2ProT) was performed at the 3′ end of the tree shrew-aligned predicted promoter-like element (Encode cCRE ID: EH38E1925959, chr18:72543433-72543672). Both the promoter (Data S2) and the complete AAV vector sequences (Data S3) are provided in the supplemental information. Custom Gateway cloning and AAV packaging was outsourced commercially. For AAV-tsC2Pro-GFP, tsC2Pro was inserted upstream of an eGFP-WPRE cassette sandwiched between two AAV2 ITR sequences and packaged as AAV1. For AAV-tsC2ProT-mScarlet3-Cre, tsC2ProT was inserted upstream of a mScarlet3-P2A-Cre-WPRE cassette sandwiched between two AAV2 ITR sequences and packaged as AAV9. Both AAVs were produced with HEK293T triple-transfection, ultrapurified with cesium chloride (CsCl) gradient ultracentrifugation and titrated with qPCR targeting AAV2 ITRs to stock concentration of >10^{13} GC/mL. The necessary quality control documentation is available from the lead contact. All sequences and plasmid maps are made available at Addgene IDs listed in the key resources table.

Epigenomic data visualization

All human brain epigenomic data was imported from the respective web servers listed in the key resources table directly to UCSC Genome Browser (hg38) as custom tracks. Tracks height and Y axis upper limit were set to maxima within the browser for the reported region in Figure 1 to enable better visualization. The final browser panel with all tracks including CBLN2 RefSeq were downloaded as vector graphics (pdf).

Tissue preparation

Three to six weeks following injections, the animals were deeply anesthetized with an overdose of Euthasol (excess of 0.25 mL/kg i.p.) and transcardially perfused with Tyrode's solution (137 mM NaCl, 2 mm KCl, 0.9 mM CaCl2, 1.2 mM MgCl2, 11.9 mM NaHCO3, 0.4 mM NaH2PO4, 5.5 mM glucose, 281 mOsm, pH 7.4) for 1–2 min, followed by 300mL of a fixative solution containing 4% paraformaldehyde (PFA) in 0.1 M phosphate buffer (pH 7.4). Subsequently, brains were blocked and sectioned coronally or sagittaly at 50–200 μm on a Leica VT 1000 S vibratome (Leica Biosystems). Sections used for electron microscopy were incubated in 1% sodium borohydride and rinsed in 0.01M PBS. Sections were then stored in 0.05% sodium azide (NaN3) in 0.01 M PBS at 4°C prior to immunohistochemistry and/or in cryoprotectant buffer (20% Glycerol, 30% Ethylene Glycol, 50% 0.1M PB) at −20°C for smRNA-FISH.

Immunohistochemistry (IHC)

Antibodies and their dilutions used in the current study are detailed in the key resources table. For confocal microscopy, sections were first blocked in 0.01M in PBS containing 1% bovine serum albumin (BSA), 0.5% Triton X- and 0.05% NaN3 for 1 h. Following rinsing with PBS, the sections were placed in the primary antibodies diluted in the same buffer for 18-36h at room temperature on an orbital shaker with slight agitation. To terminate the incubation, sections were rinsed in 0.01 M PBS and incubated in fluorophore-conjugated secondary antibodies for 4-12h. sSC Lamina borders were determined by the expression of different markers including VGluT1, VGluT2, Nissl, DAPI, Myelin and anatomical landmarks (Figures S1A–S1F) as previously noted.[1,26,71,76,115] For electron microscopy, sections were blocked with 0.01M in PBS containing 1% BSA, 0.1% Triton X- and 0.05% NaN3 for 30 min and then rinsed in PBS. Sections were then incubated in primary antibody diluted in 1% BSA and 0.05% NaN3 for 12-18h at room temperature on an orbital shaker with slight agitation. To terminate the incubation, sections were rinsed in 0.01 M PBS and incubated in biotin-conjugated secondary antibody for 4-6h.

RNA fluorescent in situ hybridization (smRNA-FISH)

RNAscope Multiplex v2 (ACD, Cat #323270) was used to detect CBLN2 transcripts. Protocol consisted of pre-treatment, FISH and IHC (where required) steps. For pre-treatment, 50 μm-thick fresh/cryopreserved tree shrew or mouse SC sections were treated with 0.3% Hydrogen Peroxide for 10 min at room temperature in a 24 well-plate. Sections were then rinsed in 0.01 M PBS and mounted on a SuperFrost+ slide (Fisher Scientific; Cat #12-550-15) and air-dried. The slide was then baked in an oven at 40°C for 30 min, followed by incubation in 4% PFA at 4°C for 15 min and dehydration in ethanol gradient (50%, 70%, 100%) at room temperature (5 min each). The slide was then air-dried and boiled first in distilled water for 10 s and then in target retrieval buffer for 5 min. After target retrieval, the slide was rinsed in distilled water and dehydrated in 100% ethanol for 3 min at room temperature, air-dried and kept in a closed container until the next steps. For FISH, sections were outlined in a hydrophobic barrier, briefly rehydrated with distilled water at room temperature and then digested with Protease IV (tree shrew) or Protease III (Mouse) at 40°C for 40 min in HybEZ II Hybridization System (ACD, Cat# 321711). Following protease treatment, sections were hybridized with tree shrew (ACD, Cat#1265181-C1) or mouse (ACD, Cat#428551) CBLN2 probes at 40°C for 2h. After probe hybridization, the sections were then treated with amplification

reagents (AMP 1–3) at 40°C for 30-30-15 min respectively. This was followed by treatment with HRP (15 min) and TSA Vivid Dye (30 min, 1:1000) at 40°C for fluorescence visualization. Finally, the slide was washed in 0.01 M PBS and either air-dried for coverslipping with Aqua-Poly/Mount Mounting medium (Polysciences, Cat #18606) or treated with blocking buffer for regular on-slide IHC as described in the respective methods subsection.

Confocal microscopy

Tissue sections were mounted on Superfrost Plus Slides (Fisher Scientific, Cat#22-037-246), air-dried, and cover slipped with Aqua-Poly/Mount Mounting medium. Images were subsequently collected using Leica Stellaris 5 laser scanning confocal microscope. Low magnification representative images were collected using HC PL FLUOTAR 5×/0.15 NA and HC PL FLUOTAR 10×/0.40 NA. High magnification images used for RNA-FISH were collected using HC PL APO 20×/0.75 NA, HC PL APO 40×/1.3 NA or HC PL APO 63×/1.4 NA. 3D datasets used for WF dendritic arbor tracing in Figures 4C–4F were acquired using HC PL APO 40×/1.3 at 4K or 6K resolution yielding a final voxel size of 0.1 × 0.1 × 0.3–1 μm. 3D datasets used for synaptic apposition analysis in Figures 4G–4J were acquired using HC PL APO 63×/1.4 NA at 4K resolution yielding a 246 × 246 μm a field of view of with final voxel size of 0.06 × 0.06 × 0.3 μm.

WF neuron tracing

Dendritic arbor tracings in Figures 4C–4F was performed in SNT Plugin for ImageJ.[100] Briefly, tiled Z-Stacks encompassing the SGS depth at the injection site (FOV Height: ≈0. 8 mm from the SC surface, FOV width: ≈2–4 mm on the lateromedial axis) were imported to SNT. First, each visualized primary dendrite was traced (as individual ‘‘paths’’) from soma until the branching point (if present), and merged in a single root, representing the soma. Then, from every branching point (often bifurcation) all secondary, tertiary and higher-order paths were traced as daughters to the path above (the parent dendrite), preserving the path order info. For visualization purposes, paths were filled using the ‘‘fill’’ command individually for each path and the fill was exported as a binarized mask. This masked stack was Z-projected (Maximum) and the original soma shape outlined using free-hand selection was added to the image. The final binary trace was pseudo colored to pink and the background was subtracted in Adobe Photoshop 2025. Insets showing location of the cells were generated by superimposing the trace to the original image with the SC borders.

Electron microscopy

Vibratome-cut 60 μm pulvinar sections from a tree shrew injected with AAV9-tsC2ProT-mScarlet3-Cre and AAV1-Ef1a-DIO-EYFP in the SC (Figure 3), were incubated in anti-GFP as described above, followed by biotinylated secondary antibody treatment to visualize AAV-expressed EYFP labeling in the pulvinar. Electron microscopy compatible staining was achieved via an incubation in Avidin-Biotin-Complex (VECTASTAIN ABC-HRP Kit, Peroxidase) solution for 2h, followed by incubation in a solution of 0.02% hydrogen peroxide (H2O2) and 0.05% diaminobenzidine (DAB) for 3–8 min. For EM resin embedding, the sections were postfixed in 1% osmium tetroxide (OsO_4) prepared in 0.1M phosphate buffer for 1 h. They were subsequently stained with 4% uranyl acetate in 70% ethanol for 1 h, followed by dehydration in 90% and 100% ethanol series and acetones, and infiltration in a 1:1 acetone-resin mixture overnight. The next day, sections were transferred to 100% resin (EMBED 812; EMS, Hatfield, PA) and incubated overnight. Sections were then flat-embedded between two Aclar sheets (EMS, Hatfield, PA) and polymerized in a 60°C oven overnight. Bright-field images from flat-embedded sections were acquired to identify the pulvinar subdivision designated for electron microscopy. Relevant areas incorporating both Pd and Pc were excised and placed into BEEM capsules (EMS, Hatfield, PA), filled with resin, and cured at 60°C for 48 h until fully polymerized. The region of interest including anatomical landmarks such as medio-lateral boundaries and capillaries was reconstructed using a camera lucida. Embedded tissue was then trimmed into a 1 × 2 mm trapezoid encompassing the sample regions of the Pd+Pc. Ultrathin sections (60 nm) were cut using an ultramicrotome (Ultracut UCT7; Leica, Buffalo Grove, IL) and collected on 400-mesh copper grids (Ted Pella, Redding, CA). Pulvinar subdivisions in the ultrathin sections were identified based on trapezoid orientation within the capsule-embedded tissue and the presence of defining landmarks. Ultrathin sections on copper grids were examined on a JEOL1010 electron microscope equipped with a 16-megapixel CCD camera (SIA). Immuno-labeled terminal images were acquired using 8,000–15,000× magnification, yielding a pixel size of 0.8–1.7 nm at the lipid bilayer resolution.

QUANTIFICATION AND STATISTICAL ANALYSIS

All data are presented as mean ± SEM unless otherwise stated. Group comparisons were performed using one-way or two-way ANOVA with Tukey's post-hoc tests unless otherwise indicated. Sample sizes are reported in figure legends. All analyses were performed using GraphPad Prism 10 and R.

Sequence alignment and transcription factor binding site analysis

Promoter sequences described above (in the section titled ‘‘AAV design and preparation’’) from human, tree shrew, and mouse were imported in FASTA format and aligned using the MUSCLE algorithm via the msa package in R. The alignment was processed using Biostrings to calculate per-species coverage and pairwise percent identity, based on ungapped aligned positions. Sequence names were standardized, and alignment statistics were computed using dplyr, tibble, and base R functions. A curated list of human TFBSs

was obtained using CIIDER tool.[101] Briefly, HuC2Pro (FASTA) was scanned using default settings for JASPAR CORE vertebrates (2020) matrix and the resulting promoter panel (CSV) was imported to R using readr. Each TFBS was mapped to the aligned human sequence by identifying corresponding ungapped positions, preserving strand orientation and using Biostrings for reverse complementation as needed. Aligned sequences from tree shrew and mouse were extracted at the mapped TFBS positions and compared to the human-aligned TFBS using a character-wise similarity score. A 70% sequence similarity threshold was used to classify TFBSs as human-specific or conserved with one or both non-human species. Classification and comparisons were performed using dplyr and stringr. TFBS conservation was visualized using ggplot2. A scatterplot was used to display the distribution of TFBSs along the aligned promoter, colored by conservation class. A complementary rug plot was used to show basewise identity between human and the tree shrew or mouse across the full alignment. Visual layouts were refined using patchwork, tidyr, and grid. Access to all data and code used here is publicly available and provided in the data and code availability section.

Calculation of AAV specificity and sensitivity

AAV specificity and sensitivity were calculated with the formulas below. Plotting and hypothesis testing were performed in GraphPad Prism (Boston, Massachusetts USA).

$$\%Specificity_{AAV1-tsC2Pro-GFP} = 100\ x \left(\frac{No\ of\ GFP^{+}CBLN2^{+}\ cells}{No\ of\ GFP^{+}\ cells}\right)$$

$$\%Sensitivity_{AAV1-tsC2Pro-GFP} = 100\ x \left(\frac{No\ of\ GFP^{+}CBLN2^{+}\ cells}{No\ of\ CBLN2^{+}\ cells}\right)$$

$$\%Specificity_{AAV9-tsC2ProT-mScarlet3-Cre} = 100\ x \left(\frac{No\ of\ EYFP^{+}CBLN2^{+}\ cells}{No\ of\ EYFP^{+}\ cells}\right)$$

$$\%Sensitivity_{AAV9-tsC2ProT-mScarlet3-Cre} = 100\ x \left(\frac{No\ of\ EYFP^{+}CBLN2^{+}\ cells}{No\ of\ EYFP^{+}\ cells}\right)$$

Dendrite reconstruction and synaptic apposition analysis

Pipeline for the dendrite reconstructions and synaptic apposition analysis was carried out in ImageJ and Imaris with four main steps. First step (Pre-processing): the multi-channel confocal z-stacks with tdTomato, VGluT1 and VGluT2 were imported into the Imaris for pre-processing (of the VGluT1 and VGluT2 signal) with the following filters: Layer Normalization, Background Subtraction (1 μm), Gaussian (0.061 μm). Second step (Segmentation): Pre-processed VGluT1 and VGluT2 channels were segmented using supervised machine learning (ML) function of the Imaris. Briefly, for each channel, "surface" objects were generated with the default settings. Using the interactive ML interface, foreground (the punctated VGluT signal) and background (the regions where VGluT signal is not expected, soma core) was marked iteratively to train the algorithm until prediction matched the ground truth (raw image). Then, the generated surface objects were used as 3D masks to binarize the signal by "Mask All" function, setting the intensity (8-bit, range: 0–255) inside of the objects to 255, and the outside to 0. The binarized VGluT channels were saved as multi-channel TIFs. Images were then imported into ImageJ, and the following operations were performed: Binarize (default), Fill Holes, Watershed, Erode, Median (Size = 1). Third Step (Tracing): SNT plugin was used to trace dendrites on the tdTomato channel as described above. Each dendrite and their daughter paths (if present) were manually traced and individually filled. The fill was then exported as a binarized mask ("Binary Fill"). Fourth Step (Colocalization): The channels for the binary VGluT1/VGluT2 signal and the binary dendrites were colocalized using the "AND" operator of the Image Calculator, yielding two separate image stacks where All VGluT1 or VGluT2 pixels contained within the dendrite i.e., synaptic appositions as described earlier.[116,117]

Additionally, we benchmarked the validity of this approach by dual-labeling VGlut2 (pre-synaptic) with PSD95 (post-synaptic) and carrying out the same pipeline. Among 438 VGluT2 appositions detected, 83.6% colocalized with PSD95 (Figure S3F). Analysis of the apposition volumes revealed that those that are PSD95- (16.4%) were the smallest volumes within the distribution, approximately 10× smaller than the PSD95- (PSD95+: 0.8647μm^3 ± 0.057, PSD95-: 0.0881μm^3 ± 0.016). Thus, we devised a volume cutoff, below which the detected putative appositions would be considered false positive. We calculated %Prediction Accuracy and %True Positive Retention for a range of cutoff values between 0 and 0.5 μm^3 with the following formulas:

$$\%Prediction\ Accuracy_{Cutoff\ =\ N} = 100\ x \left(\frac{PSD95^{+}\ appositions_{Cutoff\ =\ N}}{PSD95^{+}\ appositions_{Cutoff\ =\ N}+PSD95^{-}\ appositions_{Cutoff\ =\ N}}\right)$$

$$\%True\ Positive\ Retention_{Cutoff\ =\ N} = 100\ x \left(\frac{PSD95^{+}\ appositions_{Cutoff\ =\ N}}{PSD95^{+}\ appositions_{Cutoff\ =\ 0}}\right)$$

By calculating the point at which regression of these two functions cross, we determined the volume cutoff at which our method obtains both the highest %Prediction Accuracy and the highest %True Positive Retention (0.0218 μm^3, 90.86% $N = 21$ %Prediction

Accuracy: $R^2 = 0.6755$, $p < 0.0001$; %True Positive Retention: $R^2 = 0.9509$, $p < 0.0001$). Thus, the appositions with volumes smaller than 0.0218 μm^3 in the subsequent analysis reported in Figure 4 were considered false positive and excluded from the analysis. It is also worthy to note the previous work by others reporting PSD95- VGluT2 synapses or at least the failure of its detection thereof in the mouse cortex.[118]

Cell Reports Methods, Volume *6*

Supplemental information

CBLN2 promoter enables genetic access to wide-field neurons of the tree shrew superior colliculus

Arda Kipcak and Alev Erisir

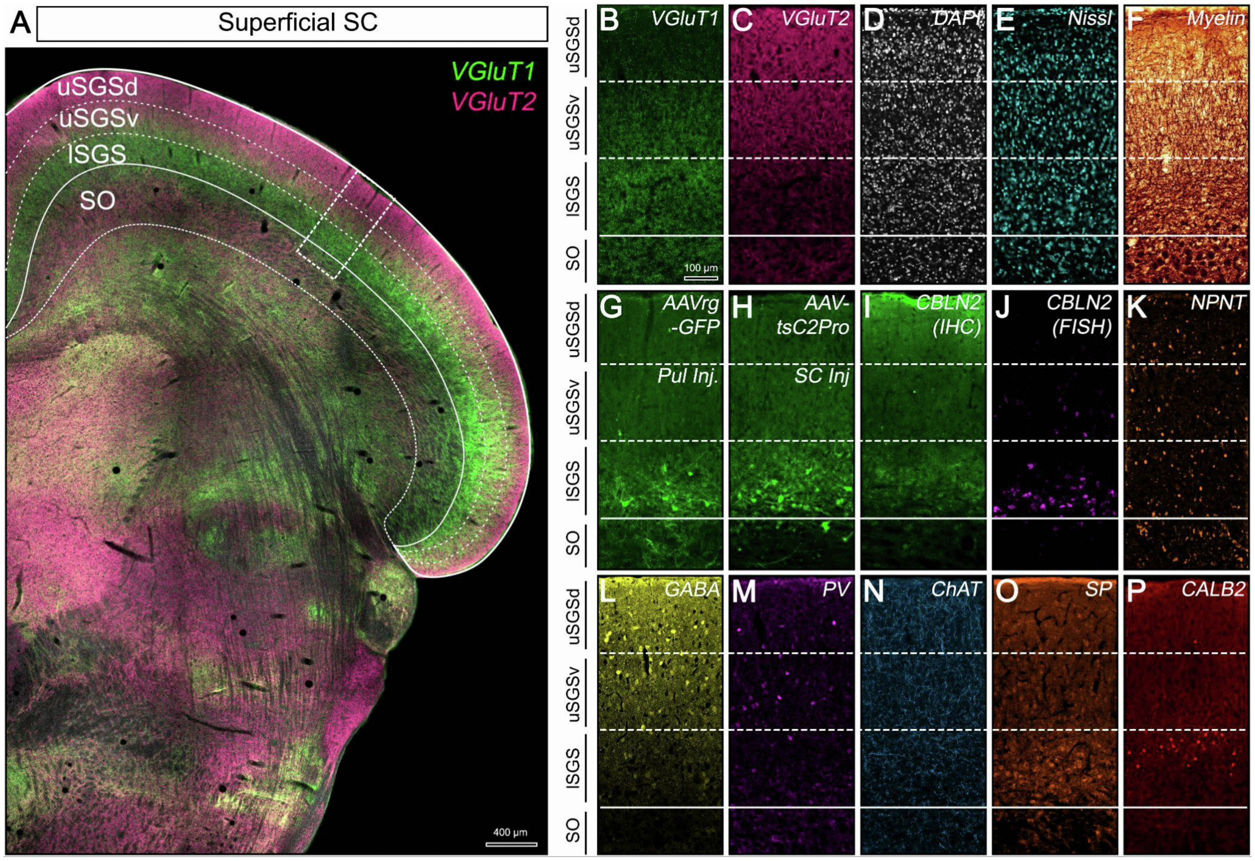

Figure S1. Expression of different markers across tree shrew superficial SC layers, related to Figure 1 and 2.

(A) Tree shrew SC coronal section highlighting superficial layers (outlined area) of SC stained with VGluT1 (Green) and VGluT2 (Purple).

(B-P) Distribution of different markers expression across the sSC. All panels are IHC staining except G: viral GFP expression; H: viral GFP expression and J: smRNA-FISH. Abbreviations (L-P): GABA: Gamma-aminobutyric acid; PV: Parvalbumin; ChAT: Choline Acetyltransferase; SP: Substance P; CALB2: Calbindin 2 (Calretinin).

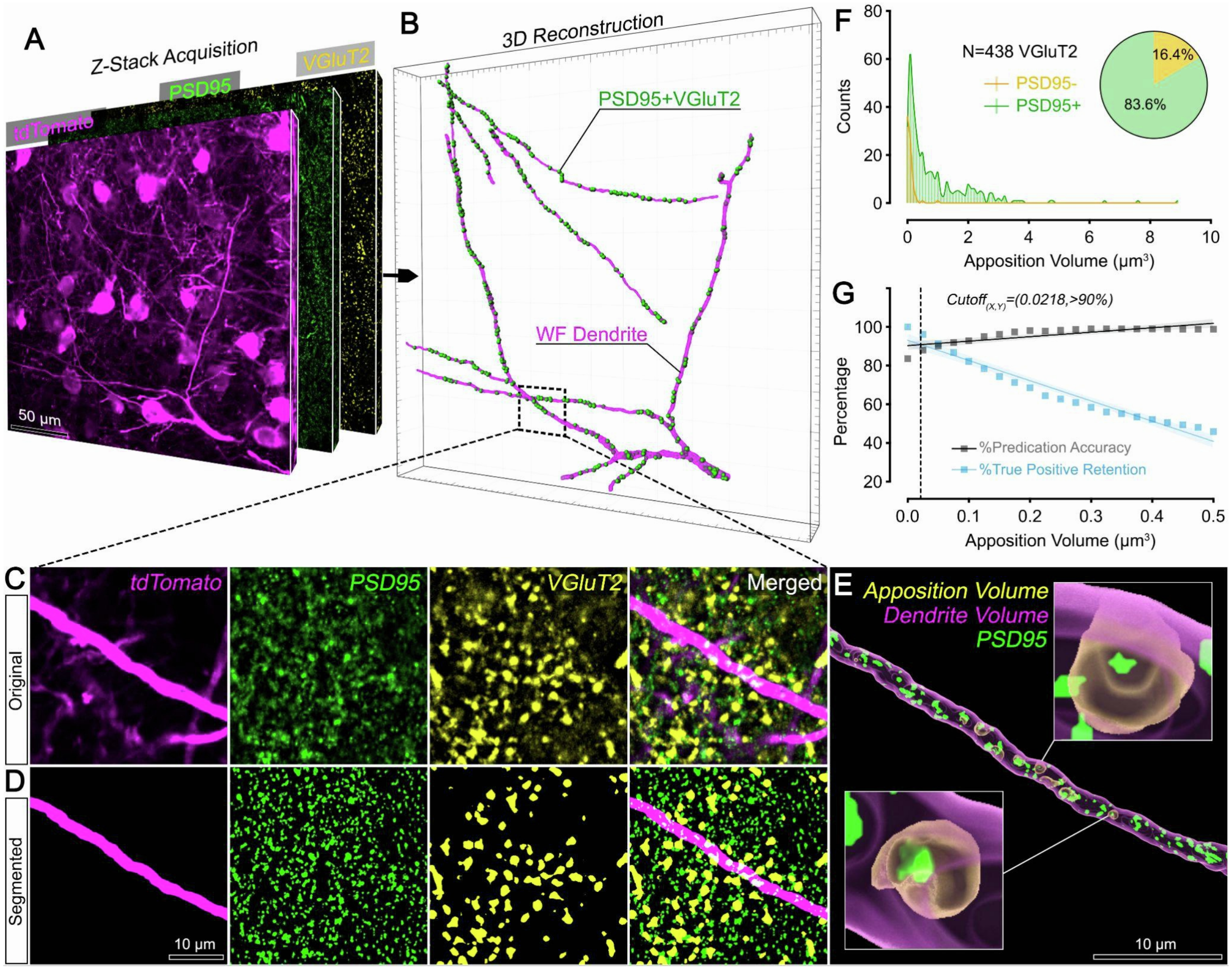

Figure S2. Experimental pipeline verification for synaptic apposition analysis, related to Figure 4.
(A) Confocal Z-Stack of viral RFP expression (AAV9-CAG-FLEX-tdTomato + AAV9-tsC2ProT-mScarlet3-Cre, magenta), IHC against PSD95 (green) and VGluT2 (yellow) to visualize WF dendrites, postsynaptic and presynaptic sites respectively.
(B) 3D reconstruction of WF dendrites (magenta), and PSD95+VGluT2 appositions (green spheres).
(C-D) Representative 2D field of view for the original image (C) and segmentation (D) of a WF dendritic branch, PSD95 and VGluT2. The merged panel at the end is used to illustrate the fidelity of the segmentation.
(E) 3D view of the same branch (magenta), showing VGluT2 appositions (yellow, i.e. not the entire VGluT2+ bouton volume), colocalizing with PSD95 (green).
(F) Distribution of PSD95+ and PSD95- VGluT2 apposition volumes, highlighting that the PSD95- appositions constitute the smallest volumes. Pie chart showing the composition of all VGluT2 appositions.
(G) X-Y plot showing the relationship between %Prediction Accuracy and %True Positive Sample Retention, as a function of (Cutoff) volumes. Solid lines with lighter colored-bands represent regressions and 95% confidence intervals respectively. Vertical dashed line shows the crossing point of the two functions (X=0.0218), that is used to filter out false-positive appositions.

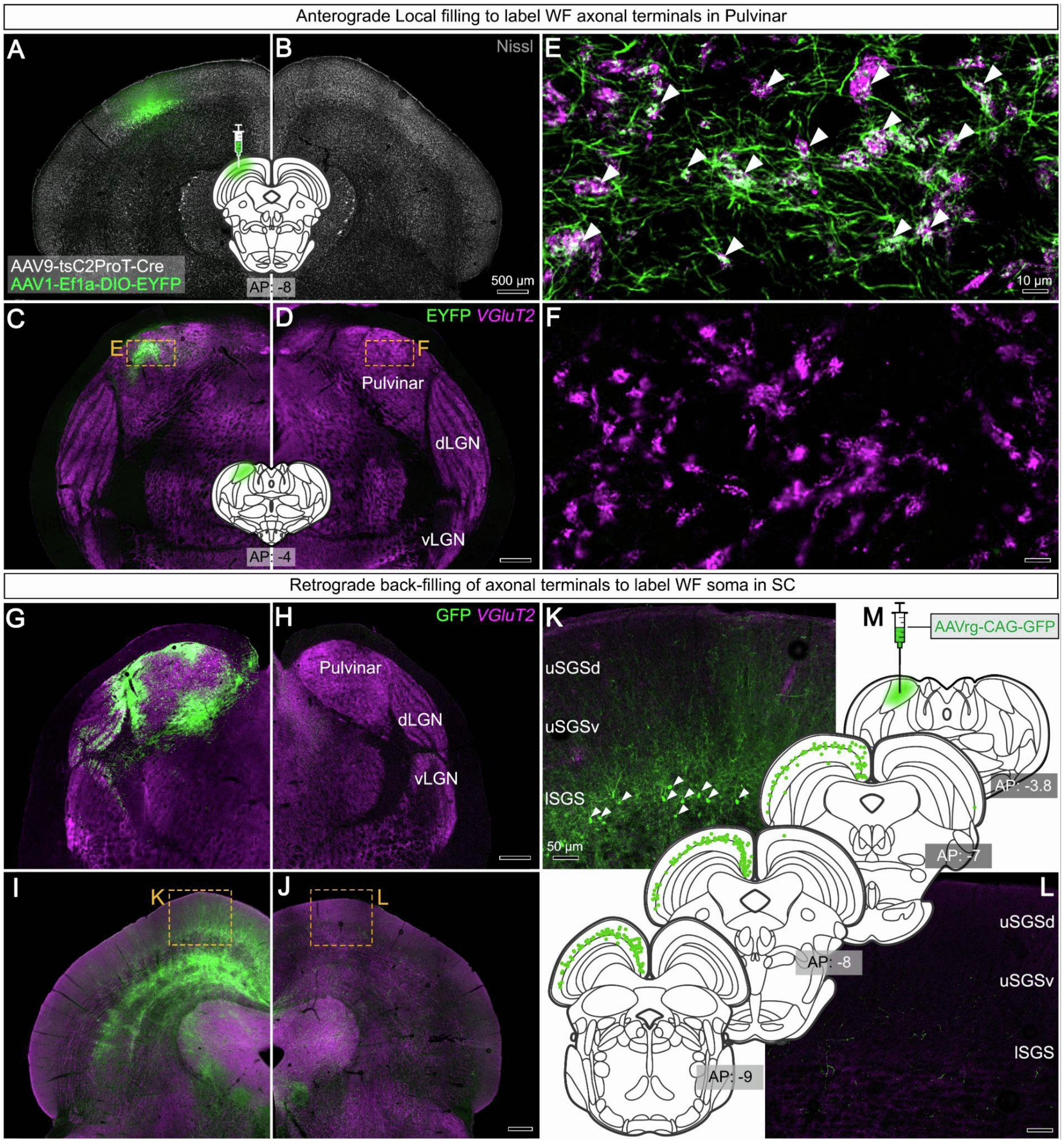

Figure S3. Tree shrew tectopulvinar projections are ipsilateral, related to Figure 3.

(A-B) Superior Colliculi showing unilateral injection of AAV9-tsC2ProT-mScarlet3-Cre + AAV1-Ef1a-DIO-EYFP in the left hemisphere. Nissl is used as counterstain.
(C-D) Thalamic sections showing EYFP-labeled axons only in the ipsilateral pulvinar. VGluT2 is used to delineate nuclei borders. dLGN: Dorsal Lateral Geniculate Nucleus; vLGN: Ventral Lateral Geniculate Nucleus.
(E-F) Higher magnification images from the ipsilateral (E) and contralateral (F) pulvinar.
(G-H) Thalamic sections showing unilateral pulvinar injection of AAVrg-CAG-GFP in the left hemisphere.
(I-J) SC sections showing retrograde GFP labeling in the ipsilateral hemisphere.
(K-L) Higher magnification images from the (K) left SC showing WF somata (white arrows) in the lower SGS and no soma labeling in the right SC (L).
(M) Schematic illustrating the pulvinar Injection and the location of the resulting retrogradely labeled WF cell bodies across the antero-posterior axis.

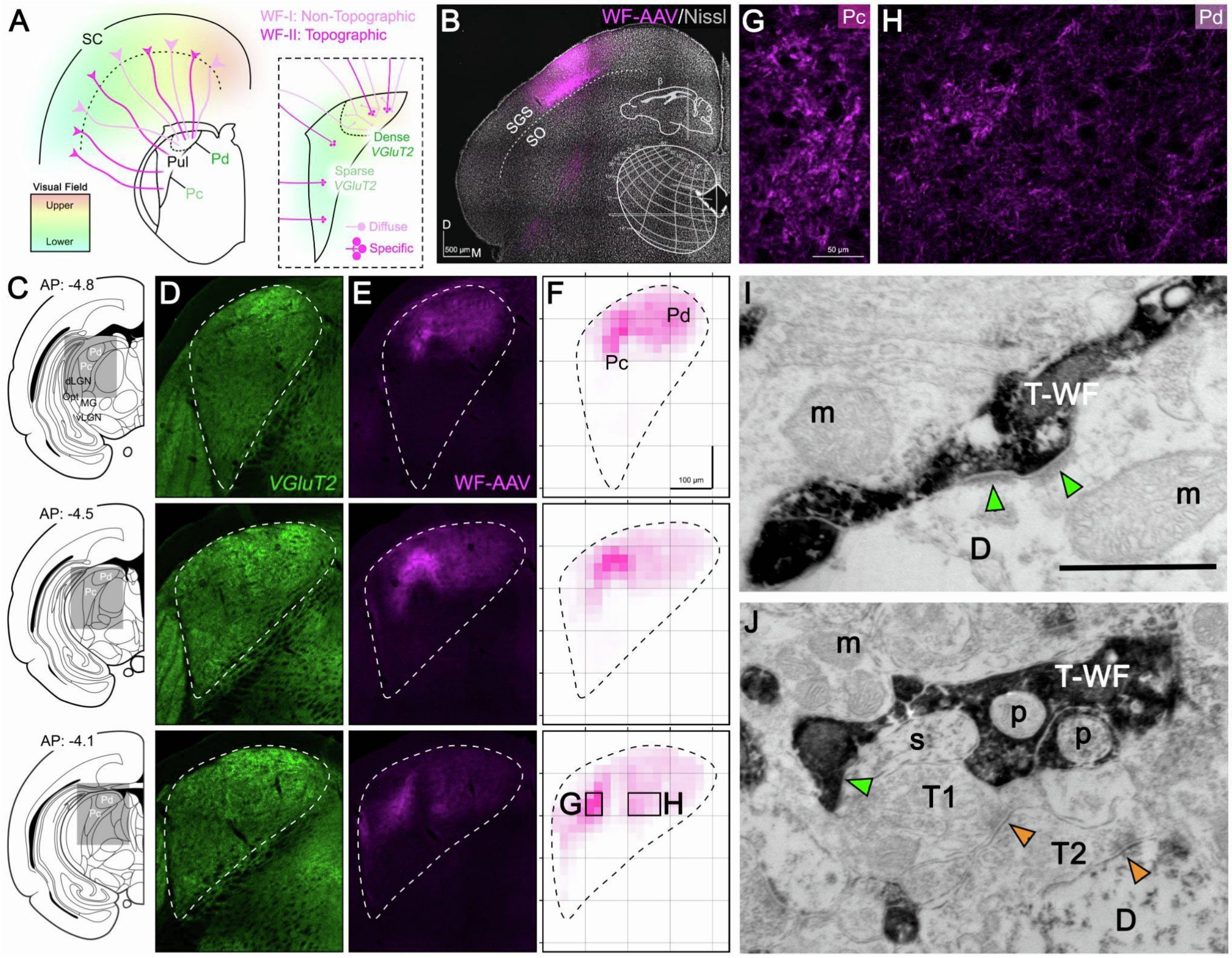

Figure S4. CBLN2 promoter-driven AAV labels two distinct tectopulvinar projection patterns, related to Figure 3.

(A) Tree shrew tectopulvinar circuit described by Luppino et al (1988)[34] and later proposed by Chomsung et al (2008)[36]. According to this model, there are two WF subtypes in the tree shrew: WF-I that non-topographically project to dorsal pulvinar (Pd) and WF-II that topographically project to the entire tectorecipient pulvinar (Pd+Pc). Visual Field topography is reflected in both structures wherein lateral to medial axis corresponds to lower to upper visual field. WF-I projections appear diffuse as unitary boutons whereas WF-II projections are specific clustered boutons. While Pc displays sparse VGluT2 originating from WF-II, Pd displays dense VGluT2 due to combined inputs from WF-I and -II.

(B) Confocal image of the SC injected with AAV9-tsC2ProT-mScarlet3-Cre+AAV1-Ef1a-DIO-EYFP (pseudocolored magenta). Inset shows the position of the section relative to parasaggittal and horizontal planes.

(C) Coronal atlas schematics showing the anatomical coordinates of imaged sections in the following panels.

(D) Pulvinar sections with VGluT2 IHC are used to help identify the borders of the pulvinar and other thalamic nuclei.

(E) Same pulvinar sections displaying the projection pattern of AAV-labeled WF axons in Pd and Pc. In all sections, fiber labeling was diffuse or diffuse+patchy in Pd and patchy in Pc.

(F) Fluorescence signal heatmaps, illustrating small intense labeling in the center and diffuse labeling in the entire Pd.

(G-H) Higher magnification image of the (G) Pc, showing clustered (specific) boutons and (H) Pd, showing both clustered (specific) boutons as well as diffusely distributed varicose fibers.

(I) Immuno-electromicrograph of AAV-labeled axon (T-WF) in Pd subdivision of pulvinar. Most axons in Pd were en passant, synapsing (green arrowheads) on large caliber dendrites (D) containing mitochondria (m). Scale bar in I=500 nm and also applies to panel J.

(J) AAV labeled axons in Pc displayed unlabeled protrusions (p), as described before by Chomsung et al (2008)[36], and formed synapses (green arrowheads) on dendrites and spines. These are often engaged in complex synaptic arrangements involving other unlabeled terminals (T1 and T2) that synapse on larger caliber dendrites (T2 ⇒D) and terminal boutons (T2⇒T1).

Data S1. CBLN2 CDS of the tree shrew, human and mouse, related to Figure 1.

Tree shrew CBLN2 CDS

```
>TSDBTID.7682.2_cds_TSDBPID.7682.2     [gene=CBLN2]     [protein=cerebellin     2     precursor]
[protein_id=TSDBPID.7682.2] [location=chr12 11137202 ~ 11147062]
ATGCCGGCGCCCGGCCGGGGCCCCTGCCGGCCGCGGCTGACCATGCCCGGGCGCCGGGGGGCGCTGCACGAGGTGGCCGGTTGCGGCTCCTGCCTGG
GGGCGGCACTGGCCCTGCTGCTGCTGCTGCTGCCCGCCTGCTGCCCGGTGCGGGCGCAGAACGACACCGAGCCCATCGTGCTGGAGGGCAAGTGCCT
GGTGGTGTGCGACTCCAGCCCGTCGGCGGACGGCGCCGTCACCTCCTCCCTGGGCATCTCCGTGCGCTCTGGCAGCGCCAAGGTGGCCTTCTCCGCC
ACGCGGAGCACCAACCACGAACCCTCCGAGATGAGCAACCGCACCATGACCATCTACTTCGACCAGGTGTTAGTAAATATTGGCAACCATTTTGATC
TTGCTTCCAGTATATTTGTAGCACCAAGAAAAGGGATTTATAGCTTCAGCTTCCACGTGGTCAAAGTGTATAACAGACAAACCATCCAGGTCAGTTT
AATGCAGAACGGCTACCCAGTGATCTCGGCGTTTGCAGGAGACCAGGACGTTACCAGGGAAGCTGCCAGCAATGGCGTCTTGCTGCTCATGGAAAGG
GAAGACAAAGTGCATCTGAAGCTGGAAAGGGGCAACCTGATGGGGGGCTGGAAGTACTCCACGTTCTCGGGCTTCTTGGTTTTTCCTCTATAA
```

Human CBLN2 CDS

```
>lcl|NM_182511.4_cds_NP_872317.1_1  [gene=CBLN2]  [db_xref=CCDS:CCDS11999.1]  [protein=cerebellin-2
precursor] [protein_id=NP_872317.1] [location=578..1252] [gbkey=CDS]
ATGCAGGCGCCCGGCCGGGGGGCCACTCGGGCTGCGGCTGATGATGCCCGGGCGCCGGGGGGCGCTGCGCGAGCCGGGCGGCTGCGGATCCTGCCTGG
GGGTGGCGCTGGCCCTGCTGTTGCTGCTACTGCCCGCCTGCTGCCCCGTGCGGGCGCAGAACGACACGGAGCCCATCGTGCTGGAGGGCAAGTGCCT
GGTGGTGTGCGACTCCAGCCCGTCGGCGGACGGCGCCGTCACCTCCTCCCTAGGCATCTCCGTGCGCTCCGGCAGCGCCAAGGTGGCCTTCTCCGCC
ACGCGGAGCACCAACCACGAGCCGTCCGAGATGAGCAACCGCACCATGACCATCTATTTCGACCAGGTATTAGTAAATATTGGCAACCACTTTGATC
TTGCTTCCAGTATATTTGTAGCACCGAGAAAAGGGATTTATAGCTTCAGCTTCCACGTGGTCAAAGTGTATAACAGACAAACCATCCAGGTCAGTTT
AATGCAGAATGGCTACCCAGTGATCTCGGCCTTTGCAGGAGACCAGGATGTCACCAGAGAAGCTGCTAGCAATGGCGTGCTGCTGCTCATGGAAAGG
GAAGACAAAGTGCATCTCAAACTTGAGAGAGGCAACCTCATGGGGGGCTGGAAATACTCCACATTCTCGGGCTTCTTGGTGTTTCCTCTATAA
```

Mouse CBLN2 CDS

```
>lcl|NM_172633.4_cds_NP_766221.1_1  [gene=Cbln2]  [db_xref=CCDS:CCDS29388.1]  [protein=cerebellin-2
precursor] [protein_id=NP_766221.1] [location=372..1046] [gbkey=CDS]
ATGCCCGCGCCTGGCCAGGGCCCCAGAGGGCCGCTGCTGAGCATGCCCGGGCGCCGGGGGGCGCTGCGTGAGCCAGCCGACTTTGGCTCCAGCCTGG
GGGCGGTGCTGGCCCTGCTGTTGCTGCTGCTGCCCGCCTGCTGCCCCGTAAGGGCTCAGAACGACACGGAGCCCATCGTGCTAGAGGGCAAGTGCCT
GGTAGTGTGCGATTCCAGCCCATCGGGGGATGGCGCCGTCACTTCTTCCCTGGGCATTTCTGTGCGCTCAGGCAGTGCCAAGGTGGCCTTCTCCGCT
ACTCGGAGCACCAACCACGAGCCGTCAGAGATGAGCAACCGTACCATGACCATCTACTTCGACCAGGTCTTAGTAAACATTGGCAACCACTTTGACC
TTGCCTCCAGTATATTTGTAGCACCAAGAAAGGGAATTTATAGCTTCAGCTTCCACGTGGTCAAAGTGTACAACAGACAAACTATCCAGGTCAGCTT
AATGCAGAATGGCTACCCGGTGATCTCTGCATTTGCCGGAGACCAGGATGTTACCAGGGAAGCAGCCAGCAATGGTGTTCTGCTGCTCATGGAAAGA
GAAGACAAAGTTCATCTCAAACTAGAGAGAGGCAACCTCATGGGAGGCTGGAAATACTCCACATTCTCGGGCTTCTTGGTTTTTCCTCTATAG
```

Data S2. CBLN2 promoter sequence of the tree shrew, human and mouse, related to Figure 1.

Tree shrew CBLN2 Promoter

```
>tsC2Pro KIZversion3 chr12:11145679-11147494:+ len=1816
GTCCCTTTAGTAAAGATTCTATCCATTTTCCACCCAAAAAACAAATCCCAGGAAGTTACTTAGGGTGAAAAAGACTAAAAAGGGAGAGAGAAAAAAA
AAAAAAAGCAAAGACAGAGGGACTGAGGAAGGAGCGAGAGACAGAGGGACCAAAGCCAAATACCCACTTTCAGGTCTCCGCAGCCGCTGTCTGATCC
CTGCGAGAAACCCCGCGTCCCCCTCTCTGCCAAGGGCAGGCTCCAGCGACGGGAATCCACGCTCGGCGTATTCCCCAGCTATTGTTCTGAGCCGGTC
CGGCCTCCAGGGCTGGGAGGCAGCACTTGTGGGGTTCGGCTAACTTACGCGTCCGTTTCGACTTTCCCCGTCCCTCTTCTCCCACTAAGCCAGACAG
GGCGTTTCAATTCCCAGCCCAGAAACAAAACGCACAGAAGCCTCCCCCCTCCCCGCCCACGGCCTCCGAACAACTCTTGCAATATTCTTTCCGAGAA
GACAGAAGTTGCTTCTCGGCTAACACCCTGTCGTCAGCCCAGGGCAGACCGACCGAGAGGCGCGCCCGCTGCCTCTGGCTCTTGCACCCCAGAACGC
AGGCTCGCCACGCTCCTCGTTAGCTCGTACGCAAGAAAGCCATGAAGCCACGATCCCGAAGCGCACGGCCGGGCTCCGTTACCTGGAACTGCCATGA
TGGGCGAGCACTGCTGTCCGCGAAGTTCTGCTCAGAGAAAATGAGGCGGACGGGAGCTGGCGGGAGGAGGACTCGGAGCGCGACCCTTCTGCCCGCA
CGGCCAATAACCGCGCCGCCCCGCCCTGCCGCTTTCCCGCGCCGGCCGAGCGGGCTCCGCACGCACCGACGCCCCGCACTCGTGCGCCGCTGCGCCC
GGTCCGGCCCGGGCTCTGGCCCCGGCTCCGGCCCCAGCTCCGGCCCCAGCTCCGGTCCCGTGCGCTGCGACCCGGCACCGCAGAAGCCCAGCGCCAC
TACCGAAGGCTTCGCGCCCGGCACGGAGCCCTCACCCAGCTTCGGCCGGCTGCTCCGCGCGGCCCGAGTGCTCCCTAACCCGCGTGTCCACAGCGCG
GCTCTGGCGGGGACCCTCAGTCCCAGCCTCCCCGCCAGACTCCCGAGGGCTCCGCCGAGCGCTGCCCTCACCCTGAGGACTCCGCGCACCGCGCTCC
TCCCGCCCTGATGCCCCAGCCCGCACCCGCCGCACCCTGCAACGCCCTGGCCAGGCTCTCACCCAAACACCCCGAGCGAAATCCTCCCAGACGCCGC
TAAGATTAGGCGCCCTCGGAAGCACCGGTCGCGTCTCTTCTTTTAGATTGTTTCTTTTCTTTTCCAAATTCCCTCCAAGTCTTAGTCTTGGATAGTG
TGACCACTTTCATAATGAAGGGGGGAAAAAACAGTTCTCTTAAGGAAAAGACTGAGATGGAGAGAAAACGCCTGGGGACGTAATGATGGGAGGGCTG
TGGTTCCGGAATCTCCAGTTTGGGAAATGCCAGGCTTTGGGCAGGGATCGAAGAGGTGCCTCTCTGGGTTTATGTGTCTGCTCCTCTGAACCTCCGG
GCTCGCGGAGCGCGTTGGCACTGGTGCTTATTTATTAAAATGCGGGTTCTGAGCCCTGATGACCGGGGAGACGCGCCGGGTTTCCGAGCTGCGCCTG
GAGCGCGGGCGAGACGCACAAGCACGGACGCGGGGCGCCACCTACGGGCGCTGGGGCGTCCCTGCCGCAGGCTGCGGGCAAGGACTTGGCAGAAGAG
GGGGAGTCCTAGAGACAAGGACGAGGTTTGCTGATGGGCTTTCACCCCAAAAAACAGTGCTGGCCTCTGC
```

Human CBLN2 Promoter

```
>HuC2Pro hg38 chr18:72542815-72544612 strand=+ len=1798
GTCCCTTTGGTACAGATTTTATCTGTTTTCCACCCACAGAACAAATCCCAGGAATCTCCTTGGGGCGAATAAGATTACTAGGGGGGAAATGACACCA
CACACACACACACACACACACACACACACACACACACACACACACACGGGCAGTAAAGCCAAACACCTACTTCCAAGGTCCCTGCAGCCACTAGCGA
GCCCCCTTGGGTGAAACCCCTGGGATTCCTCTCTTAGGCGAAGGACCGATTCCAACGACGGAATCCCAGGCTTGGGTTATTCTCCAGCTCTGGTTTC
CAGACCACGGGGATTCTCTCTTTCTCAGCGGGGCGGCAGCCCCTGCAGGTTTCTGCGAACTTACGCGCCCGTCTGCACTTTTGCCCCGTCCCCGCTC
CTCCCAGGAAAGCAGACAGGCCATTTCAATACCAGCCCAGAGACACGCAGAGAAGCCGCCCCCTCGCCGCCCACAGCCTCCCATTAACTCTTCCAGT
ATTCTTTCTAAGAACAGAGAAGTTGGCTCTTGATAAATATCCGCTGTCCGCAGCCCCGATCCTACATGATTTCCCTTCTCTCTCTCCACCTCCTCCA
CCCCTCGATCTGGACCAGGGAGAGTCTTCGTTAAAATCCACGCAGAAGGCGCTTGCATGCGGAGGGGAGGGCAGGTCGGGGGTGGTTACCTGGAACA
TCCATGCTGGGCGAGCTCCGCTGTCCGCGAAGTTGCTCTGCTTAGAGAAAATGAGGCGAGTGGGAGCTGTCGGGAGGAGGACACGGAGCGCGACCCT
GCTCCCAGCGCGTGGCCAATAACCGCGCCGCCCCGCCCTGCCGCTTTCCCGCGCCAGCCTGCGCCGCTTCAGGGGTGCACCACGCCCCGCGCGCCCG
CTTAGGCGCCGCGCCCGGGACCGGGAACCCCGCGTCTCGCCCGGCTCAGCGCCCCGCGCTGTGCGCCCAGGTGCCTCCAACCCCTGGGCTTCGCGCC
CGCACCGCTGCCTGGGGCCCCTCGAGCTCCCGCGCTCAGCGCGTCCGCAGCGCGGCTCCCTCGCGGGTCCCCTCGGCCCCGCAGCCCCGCCAGTCTC
CAGAGGGCATAGCCGAGCGCTGCCGCCTCCCTAGGACTCGGAACCTTCCCTGCTCCCAAGCCCGCCGCACCCTCCAACGCCCCGGTCCTGCTCACCC
AAACGCCCAGAGCAAAATACTCCCAGACGTCTCTTTTGAAAATTAGACACCCTTAAAAGCACCGGTCGCTTCTCTTCTTTTAGATTCTTTCTTTTCT
TTCCCCAATTCCCTCCAAGTCTTAATATTGAATGGCGTGACCACTTTCATAATGACAGGAGCGAAAAAAACAATAAGTTAAAAAAAAAAAAAAGAAG
AAGAAGACTGGGATGGCTGGGACGAAGAGAGGGAAAAAAAAAGCTTGAGAATTTGATGATTTAGGAGCTGTGGTTCCAGAGTCCCAGTTGGGGAACT
GTCCGGTTCCCGCAGGGCTAGAAGAGGGGCCTCCGGCCCGGGTCTGTGTGTCTGCTCCTCTGGACCTCGGACTGGTGGAGCGGCTGGCGCTTGCGCT
TATTTATTAAATTGCGGTGTCCGCGCTCGCTCAAAGGACCCGGGGAGACGCGCTGGGTTTCCCAGCTGCGCCTGGAGCTCGGCTGAGGGCGCAAACA
CGGACGTGGGGCGCCACCTGCGGGTGCCCGGGCCTCCCTGCCGCAGGCTGCGGACCGGCGGACTCCGACAGAAGACGGGGGAGTCCTGGCCGCAAGG
CTGAGGTTTGCTGATGGTCTTCTTTCACCCCAAAAGCAGCGCTGACCTCTGC
```

Mouse CBLN2 Promoter

```
>MsC2Pro mm39 chr18:86728752-86729905 strand=+ len=1154
CTGTTTTAGTTGGGGTGAAGGAAAAGAAAATCCTCAAACCTCACCTTTGTCCCTGGGTACTCCCCCATACTGAGTAGGCTTGTTCGCAGCCCGAGGA
CGGCAGGCTCCAGCGCCCATAGGTGGCGCCCCTCGTCCGTGCTTGCGCATCTCCCGGCTCTCCAGGCGCAGCTCGGAACCCCGCGTGTCTCCCCGGG
TCTTTAGAGCCCAGAGACTGCGTTTTAATAAATACGAGTGGTGCCAACGCGCTCCAGCCCCGGGGTTCAGAGCAGCAGACACATAAACCCAGGCAGG
AAGAGCCTCTTTAATGTCTGCCAAAAGCTGACATTTCACTAACCGGAGATTTTGGAATCGCAGTTTAAAACCATTAAATTACCGAGGCTTTTCCTCT
CTTAAAAAATACTTTTCCTTTTTTTTTTTTTTTTTTTTTTTTTTTTGGTACCCTCTCCTCTTTAAAATTATGAATGTGGCCACTCCGTTCAAGATCA
AGACTTGGAGGGAATTTGGAAAAGAAAAAGAATCTGGAAGAAGAAACTCGGCTGGTGCTTCGGAGGGCGCCTAGTTTTAAGAAGGTACCTAAGAGAA
CTGTCCTAGATGTCTAGGTGAGAGACGGATATCGGGGCTTGGCAAGGTGCTACGGGGAAGTCACAGGGGACTCCACAGGGCTGTGGATTCTAGCACG
GAGGGAGCGAGGAGTCCTTAGTTGCCAGCTCCGAGCTGTGCTTTCTGGAGACTGGAGCTGAGGAGGGGACCCAGGGATCCTGTTATTGAGGTTCAGG
ATAGGTGTACTGACTGGGGAACTATTCCGGACCCGAAGGAGGCAAACTGCGCGCCTTGGTGCGGTGCGGCTCCTGGCTTCGGCGCGGCTCAACCCGA
GCGGGTAGCAAGTAGGTTCCAGGCACGGTCGTGCGTGCGCTGAGCAGCGCTCGGTGTGGGGCTCTCTGGGCTCTCGGGGCCGGGGCGTGGTGTGCTT
GCGGAGCGGGATCGGACCGAGAGGAGAGGAGGCAGGGCGGGGCGGAGCAGTTATTGGCCAGGCGCTGGGAGAAGGGTCGTGCTCCGAGGTGTCCTCC
TCCCGCTAGCTCCCGCCTGCCTCATTTTCTCTAAGCAGAACAACTTCTCCGACATCGGTACTCGCCCGTCATGGACATTCCAGGTAA
```

Data S3. CBLN2-promoter driven AAV vector sequences, related to Figure 2 and 3

AAV1-tsC2Pro-GFP (6345 bp)

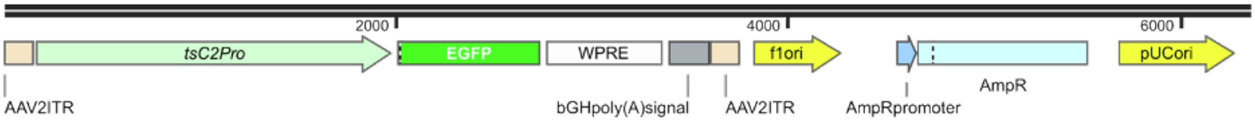


```
cctgcaggcagctgcgcgctcgctcgctcactgaggccgcccgggcaaagcccgggcgtcgggcgacctttggtcgcccggcctcagtgagcgagcgagcgcgcagagagg
gagtggccaactccatcactaggggttccttctagacaactttgtatagaaaagttggcagaggccagcactgtttttttggggtgaaagcccatcagcaaacctcgtcctt
gtctctaggactcccccctcttctgccaagtccttgcccgcagcctgcggcagggacgccccagcgcccgtaggtggcgccccgcgtccgtgcttgtgcgtctcgcccgcgc
tccaggcgcagctcggaaacccggcgcgtctccccggtcatcagggctcagaacccgcattttaataaataagcaccagtgccaacgcgctccgcgagcccggaggttcag
aggagcagacacataaacccagagaggcacctcttcgatccctgcccaaagcctggcatttcccaaactggagattccggaaccacagccctcccatcattacgtccccag
gcgttttctctccatctcagtcttttccttaagagaactgtttttttcccccttcattatgaaagtggtcacactatccaagactaagacttggagggaatttggaaaaga
aaagaaacaatctaaaagaagagacgcgaccggtgcttccgagggcgcctaatcttagcggcgtctgggaggatttcgctcggggtgtttgggtgagagcctggccagggc
gttgcagggtgcggcgggtgcgggctggggcatcagggcgggaggagcgcggtgcgcggagtcctcagggtgagggcagcgctcggcggagccctcgggagtctggcgggg
aggctgggactgagggtccccgccagagccgcgctgtggacacgcgggttagggagcactcggccgcgcggagcagccggccgaagctgggtgagggctccgtgccgggc
gcgaagccttcggtagtggcgctgggcttctgcggtgccgggtcgcagcgcacgggaccggagctggggccggagctggggccggagccggggccagagcccgggccggac
cgggcgcagcggcgcacgagtgcggggcgtcggtgcgtgcggagcccgctcggccggcgcgggaaagcggcagggcggggcggcgcggttattggccgtgcggcagaagg
gtcgcgctccgagtcctcctcccgccagctcccgtccgcctcatttctctgagcagaacttcgcggacagcagtgctcgcccatcatggcagttccaggtaacggagccc
ggccgtgcgcttcgggatcgtggcttcatggctttcttgcgtacgagctaacgaggagcgtggcgagcctgcgttctggggtgcaagagccagaggcagcgggcgcgcctc
tcggtcggtctgccctgggctgacgacagggtgttagccgagaagcaacttctgtcttctcggaaagaatattgcaagagttgttcggaggccgtgggcggggagggggga
ggcttctgtgcgttttgtttctgggctgggaattgaaacgccctgtctggcttagtgggagaagagggacggggaaagtcgaaacggacgcgtaagttagccgaaccccac
aagtgctgcctcccagccctggaggccggaccggctcagaacaatagctggggaatacgccgagcgtggattcccgtcgctggagcctgcccttggcagagaggggggacgc
ggggtttctcgcagggatcagacagcggctgcggagacctgaaagtgggtatttggctttggtccctctgtctctcgctccttcctcagtccctctgtctttgcttttttt
tttttttctctctcccttttagtctttttcaccctaagtaacttcctgggatttgtttttttgggtggaaaatggatagaatctttactaaagggaccaagtttgtacaaa
aaagcaggctgccaccatggtgagcaagggcgaggagctgttcaccggggtggtgcccatcctggtcgagctggacggcgacgtaaacggccacaagttcagcgtgtccgg
cgagggcgagggcgatgccacctacggcaagctgaccctgaagttcatctgcaccaccggcaagctgcccgtgccctggcccaccctcgtgaccaccctgacctacggcgt
gcagtgcttcagccgctaccccgaccacatgaagcagcacgacttcttcaagtccgccatgcccgaaggctacgtccaggagcgcaccatcttcttcaaggacgacggcaa
ctacaagacccgcgccgaggtgaagttcgagggcgacaccctggtgaaccgcatcgagctgaagggcatcgacttcaaggaggacggcaacatcctggggcacaagctgga
gtacaactacaacagccacaacgtctatatcatggccgacaagcagaagaacggcatcaaggtgaacttcaagatccgccacaacatcgaggacggcagcgtgcagctcgc
cgaccactaccagcagaacacccccatcggcgacggccccgtgctgctgcccgacaaccactacctgagcacccagtccgccctgagcaaagaccccaacgagaagcgcga
tcacatggtcctgctggagttcgtgaccgccgccgggatcactctcggcatggacgagctgtacaagtaaacccagctttcttgtacaaagtgggaattccgataatcaac
ctctggattacaaaatttgtgaaagattgactggtattcttaactatgttgctccttttacgctatgtggatacgctgctttaatgcctttgtatcatgctattgcttccc
gtatggctttcattttctcctccttgtataaatcctggttgctgtctctttatgaggagttgtggcccgttgtcaggcaacgtggcgtggtgtgcactgtgtttgctgacg
caacccccactggttggggcattgccaccacctgtcagctcctttccgggactttcgctttccccctccctattgccacggcggaactcatcgccgcctgccttgcccgct
gctggacaggggctcggctgttgggcactgacaattccgtggtgttgtcggggaagctgacgtcctttccatggctgctcgcctgtgttgccacctggattctgcgcggga
cgtccttctgctacgtcccttcggccctcaatccagcggaccttccttcccgcggcctgctgccggctctgcggcctcttccgcgtcttcgccttcgccctcagacgagtc
ggatctccctttgggccgcctccccgcatcgggaattcctagagctcgctgatcagcctcgactgtgccttctagttgccagccatctgttgtttgcccctcccccgtgcc
ttccttgaccctggaaggtgccactcccactgtcctttcctaataaaatgaggaaattgcatcgcattgtctgagtaggtgtcattctattctggggggtggggtggggca
ggacagcaagggggaggattgggaagagaatagcaggcatgctggggagggccgcaggaacccctagtgatggagttggccactccctctctgcgcgctcgctcgctcact
gaggccgggcgaccaaaggtcgcccgacgcccgggctttgcccgggcggcctcagtgagcgagcgagcgcgcagctgcctgcaggggcgcctgatgcggtattttctcctt
acgcatctgtgcggtatttcacaccgcatacgtcaaagcaaccatagtacgcgccctgtagcggcgcattaagcgcggcgggtgtggtggttacgcgcagcgtgaccgcta
cacttgccagcgccttagcgcccgctcctttcgctttcttcccttcctttctcgccacgttcgccggctttccccgtcaagctctaaatcgggggctccctttagggttcc
gatttagtgctttacggcacctcgaccccaaaaaacttgatttgggtgatggttcacgtagtgggccatcgccctgatagacggtttttcgccctttgacgttggagtcca
cgttctttaatagtggactcttgttccaaactggaacaacactcaactctatctcgggctattcttttgatttataagggattttgccgatttcggtctattggttaaaaa
atgagctgatttaacaaaaatttaacgcgaattttaacaaaatattaacgtttacaattttatggtgcactctcagtacaatctgctctgatgccgcatagttaagccagc
cccgacacccgccaacacccgctgacgcgccctgacgggcttgtctgctcccggcatccgcttacagacaagctgtgaccgtctccgggagctgcatgtgtcagaggtttt
caccgtcatcaccgaaacgcgcgagacgaaagggcctcgtgatacgcctatttttataggttaatgtcatgataataatggtttcttagacgtcaggtggcacttttcggg
gaaatgtgcgcggaacccctatttgtttatttttctaaatacattcaaatatgtatccgctcatgagacaataaccctgataaatgcttcaataatattgaaaaaggaaga
gtatgagtattcaacatttccgtgtcgcccttattcccttttttgcggcattttgccttcctgtttttgctcacccagaaacgctggtgaaagtaaaagatgctgaagatc
agttgggtgcacgagtgggttacatcgaactggatctcaacagcggtaagatccttgagagttttcgccccgaagaacgttttccaatgatgagcacttttaaagttctgc
tatgtggcgcggtattatcccgtattgacgccgggcaagagcaactcggtcgccgcatacactattctcagaatgacttggttgagtactcaccagtcacagaaaagcatc
ttacggatggcatgacagtaagagaattatgcagtgctgccataaccatgagtgataacactgcggccaacttacttctgacaacgatcggaggaccgaaggagctaaccg
cttttttgcacaacatgggggatcatgtaactcgccttgatcgttgggaaccggagctgaatgaagccataccaaacgacgagcgtgacaccacgatgcctgtagcaatgg
caacaacgttgcgcaaactattaactggcgaactacttactctagcttcccggcaacaattaatagactggatggaggcggataaagttgcaggaccacttctgcgctcgg
cccttccggctggctggtttattgctgataaatctggagccggtgagcgtggaagccgcggtatcattgcagcactggggccagatggtaagccctcccgtatcgtagtta
tctacacgacggggagtcaggcaactatggatgaacgaaatagacagatcgctgagataggtgcctcactgattaagcattggtaactgtcagaccaagtttactcatata
tactttagattgatttaaaacttcatttttaatttaaaaggatctaggtgaagatcctttttgataatctcatgaccaaaatcccttaacgtgagttttcgttccactgag
cgtcagaccccgtagaaaagatcaaaggatcttcttgagatcctttttttctgcgcgtaatctgctgcttgcaaacaaaaaaaccaccgctaccagcggtggtttgtttgc
cggatcaagagctaccaactctttttccgaaggtaactggcttcagcagagcgcagataccaaatactgttcttctagtgtagccgtagttaggccaccacttcaagaact
ctgtagcaccgcctacatacctcgctctgctaatcctgttaccagtggctgctgccagtggcgataagtcgtgtcttaccgggttggactcaagacgatagttaccggata
aggcgcagcggtcgggctgaacggggggttcgtgcacacagcccagcttggagcgaacgacctacaccgaactgagatacctacagcgtgagctatgagaaagcgccacgc
ttcccgaagggagaaaggcggacaggtatccggtaagcggcagggtcggaacaggagagcgcacgagggagcttccagggggaaacgcctggtatctttatagtcctgtcg
ggtttcgccacctctgacttgagcgtcgatttttgtgatgctcgtcaggggggcggagcctatggaaaaacgccagcaacgcggcctttttacggttcctggccttttgct
ggccttttgctcacatgt
```

Data S3. CBLN2-promoter driven AAV vector sequences, related to Figure 2 and 3, continued.

AAV9-tsC2ProT-mScarlet3-Cre (6775 bp)

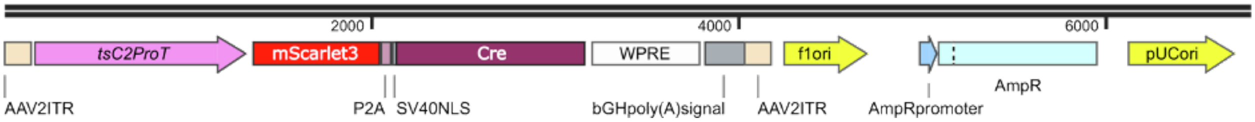

cctgcaggcagctgcgcgctcgctcgctcactgaggccgcccgggcaaagcccgggcgtcgggcgacctttggtcgcccggcctcagtgagcgagcgagcgcgcagagagg
gagtggccaactccatcactaggggttccttctagacaactttgtatagaaaagttggcagaggccagcactgtttttggggtgaaagcccatcagcaaacctcgtcctt
gtctctaggactcccccctcttctgccaagtccttgcccgcagcctgcggcagggacgccccagcgcccgtaggtggcgccccgcgtccgtgcttgtgcgtctcgcccgcgc
tccaggcgcagctcggaaacccggcgcgtctccccggtcatcagggctcagaacccgcattttaataaataagcaccagtgccaacgcgctccgcgagcccggaggttcag
aggagcagacacataaacccagagaggcacctcttcgatccctgcccaaagcctggcatttcccaaactggagattccggaaccacagccctcccatcattacgtccccag
gcgttttctctccatctcagtcttttccttaagagaactgtttttcccccttcattatgaaagtggtcacactatccaagactaagacttggagggaatttggaaaaga
aaagaaacaatctaaaagaagagacgcgaccggtgcttccgagggcgcctaatcttagcggcgtctgggaggatttcgctcggggtgtttgggtgagagcctggccagggc
gttgcagggtgcggcgggtgcgggctggggcatcagggcgggaggagcgcggtgcgcggagtcctcagggtgagggcagcgctcggcggagccctcgggagtctggcgggg
aggctgggactgagggtccccgccagagccgcgctgtggacacgcgggttagggagcactcgggccgcgcggagcagccggccgaagctgggtgagggctccgtgccgggc
gcgaagccttcggtagtggcgctgggcttctgcggtgccgggtcgcagcgcacgggaccggagctggggccggagctggggccggagccggggccagagcccgggccggac
cgggcgcagcggcgcacgagtgcggggcgtcggtgcgtgcggagcccgctcggccggcgcgggaaagcggcagggcggggcggcgcggttattggccgtgcgggcagaagg
gtcgcgctccgagtcctcctcccgccagctcccgtccgcctcattttctctgagcagaacttcgcggacagcagtgctcgcccatcatggcagttccaggtaaccaagttt
gtacaaaaaagcaggctgccaccatggatagcaccgaggcagtgatcaaggagttcatgcggttcaaggtgcacatggagggctccatgaacggccacgagttcgagatcg
agggcgagggcgagggccgcccctacgagggcacccagaccgccaagctgagggtgaccaagggtggcccccctgcccttctcctgggacatcctgtcccctcagttcatgt
acggctccagggccttcacgaagcaccccgccgacatccccgactactggaagcagtccttccccgagggcttcaagtgggagcgcgtgatgaacttcgaggacggcggcg
ccgtgtccgtggcccaggacacctccctggaggacggcaccctgatctacaaggtgaagctccgcggcaccaacttccctcctgacggccccgtaatgcagaagaagacaa
tgggctgggaagcatccaccgagcggttgtaccccgaggacgtcgtgctgaagggcgacattaagatggccctgcgcctgaaggacggcggccgctacctggcggacttca
agaccacctacagggccaagaagcccgtgcagatgcccggcgccttcaacatcgaccgcaagttggacatcacatcccacaacgaggactacaccgtggtggaacagtacg
aacgctccgtggcccgccactccaccggcggctccggtggctccggaagcggagccacgaacttctctctgttaaagcaagcaggagatgttgaagaaaaccccggccta
tgggcccaaagaagaagagaaaggtttcgaatttactgaccgtacaccaaaatttgcctgcattaccggtcgatgcaacgagtgatgaggttcgcaagaacctgatggaca
tgttcagggatcgccaggcgttttctgagcatacctggaaaatgcttctgtccgtttgccggtcgtgggcggcatggtgcaagttgaataaccggaaatggtttcccgcag
aacctgaagatgttcgcgattatcttctatatcttcaggcgcgcggtctggcagtaaaaactatccagcaacatttgggccagctaaacatgcttcatcgtcggtccgggc
tgccacgaccaagtgacagcaatgctgtttcactggttatgcggcggatccgaaaagaaaacgttgatgccggtgaacgtgcaaaacaggctctagcgttcgaacgcactg
atttcgaccaggttcgttcactcatggaaaatagcgatcgctgccaggatatacgtaatctggcatttctggggattgcttataacaccctgttacgtatagccgaaattg
ccaggatcagggttaaagatatctcacgtactgacggtgggagaatgttaatccatattggcagaacgaaaacgctggttagcaccgcaggtgtagagaaggcacttagcc
tgggggtaactaaactggtcgagcgatggatttccgtctctggtgtagctgatgatccgaataactacctgttttgccgggtcagaaaaaatggtgttgccgcgccatctg
ccaccagccagctatcaactcgcgccctggaagggatttttgaagcaactcatcgattgatttacggcgctaaggatgactctggtcagagatacctggcctggtctggac
acagtgcccgtgtcggagccgcgcgagatatggcccgcgctggagtttcaataccggagatcatgcaagctggtggctggaccaatgtaaatattgtcatgaactatatcc
gtaacctggatagtgaaacaggggcaatggtgcgcctgctggaagatggcgattagacccagctttcttgtacaaagtgggaattccgataatcaacctctggattacaaa
atttgtgaaagattgactggtattcttaactatgttgctccttttacgctatgtggatacgctgctttaatgcctttgtatcatgctattgcttcccgtatggctttcatt
ttctcctccttgtataaatcctggttgctgtctctttatgaggagttgtggcccgttgtcaggcaacgtggcgtggtgtgcactgtgtttgctgacgcaacccccactggt
tggggcattgccaccacctgtcagctcctttccgggactttcgctttccccctccctattgccacggcggaactcatcgccgcctgccttgcccgctgctggacaggggct
cggctgttgggcactgacaattccgtggtgttgtcggggaagctgacgtcctttccatggctgctcgcctgtgttgccacctggattctgcgcgggacgtccttctgctac
gtcccttcggccctcaatccagcggaccttccttcccgcggcctgctgccggctctgcggcctcttccgcgtcttcgccttcgccctcagacgagtcggatctccctttgg
gccgcctccccgcatcgggaattcctagagctcgctgatcagcctcgactgtgccttctagttgccagccatctgttgtttgcccctcccccgtgccttccttgaccctgg
aaggtgccactcccactgtcctttcctaataaaatgaggaaattgcatcgcattgtctgagtaggtgtcattctattctggggggtggggtggggcaggacagcaagggggg
aggattgggaagagaatagcaggcatgctggggagggccgcaggaacccctagtgatggagttggccactccctctctgcgcgctcgctcgctcactgaggccgggcgacc
aaaggtcgcccgacgcccgggctttgcccgggcggcctcagtgagcgagcgagcgcgcagctgcctgcaggggcgcctgatgcggtattttctccttacgcatctgtgcgg
tatttcacaccgcatacgtcaaagcaaccatagtacgcgccctgtagcggcgcattaagcgcggcgggtgtggtggttacgcgcagcgtgaccgctacacttgccagcgcc
ttagcgcccgctcctttcgctttcttcccttcctttctcgccacgttcgccggctttccccgtcaagctctaaatcgggggctccctttagggttccgatttagtgcttta
cggcacctcgaccccaaaaaacttgatttgggtgatggttcacgtagtgggccatcgccctgatagacggtttttcgccctttgacgttggagtccacgttctttaatagt
ggactcttgttccaaactggaacaacactcaactctatctcggctattcttttgatttataagggattttgccgatttcggtctattggttaaaaaatgagctgatttaa
caaaaatttaacgcgaattttaacaaaatattaacgtttacaattttatggtgcactctcagtacaatctgctctgatgccgcatagttaagccagccccgacacccgcca
acacccgctgacgcgccctgacgggcttgtctgctcccggcatccgcttacagacaagctgtgaccgtctccgggagctgcatgtgtcagaggttttcaccgtcatcaccg
aaacgcgcgagacgaaagggcctcgtgatacgcctatttttataggttaatgtcatgataataatggtttcttagacgtcaggtggcacttttcggggaaatgtgcgcgga
acccctatttgtttatttttctaaatacattcaaatatgtatccgctcatgagacaataaccctgataaatgcttcaataatattgaaaaaggaagagtatgagtattcaa
catttccgtgtcgcccttattcccttttttgcggcattttgccttcctgtttttgctcacccagaaacgctggtgaaagtaaaagatgctgaagatcagttgggtgcacga
gtgggttacatcgaactggatctcaacagcggtaagatccttgagagttttcgccccgaagaacgttttccaatgatgagcacttttaaagttctgctatgtggcgcggta
ttatcccgtattgacgccgggcaagagcaactcggtcgccgcatacactattctcagaatgacttggttgagtactcaccagtcacagaaaagcatcttacggatggcatg
acagtaagagaattatgcagtgctgccataaccatgagtgataacactgcggccaacttacttctgacaacgatcggaggaccgaaggagctaaccgcttttttgcacaac
atgggggatcatgtaactcgccttgatcgttgggaaccggagctgaatgaagccataccaaacgacgagcgtgacaccacgatgcctgtagcaatggcaacaacgttgcgc
aaactattaactggcgaactacttactctagcttcccggcaacaattaatagactggatggaggcggataaagttgcaggaccacttctgcgctcggcccttccggctggc
tggtttattgctgataaatctggagccggtgagcgtggaagccgcggtatcattgcagcactggggccagatggtaagccctcccgtatcgtagttatctacacgacgggg
agtcaggcaactatggatgaacgaaatagacagatcgctgagataggtgcctcactgattaagcattggtaactgtcagaccaagtttactcatatatactttagattgat
ttaaaacttcatttttaatttaaaaggatctaggtgaagatcctttttgataatctcatgaccaaaatcccttaacgtgagttttcgttccactgagcgtcagaccccgta
gaaaagatcaaaggatcttcttgagatcctttttttctgcgcgtaatctgctgcttgcaaacaaaaaaaccaccgctaccagcggtggtttgtttgccggatcaagagcta
ccaactctttttccgaaggtaactggcttcagcagagcgcagataccaaatactgttcttctagtgtagccgtagttaggccaccacttcaagaactctgtagcaccgcct
acatacctcgctctgctaatcctgttaccagtggctgctgccagtggcgataagtcgtgtcttaccgggttggactcaagacgatagttaccggataaggcgcagcggtcg
ggctgaacggggggttcgtgcacacagcccagcttggagcgaacgacctacaccgaactgagatacctacagcgtgagctatgagaaagcgccacgcttcccgaagggaga
aaggcggacaggtatccggtaagcggcagggtcggaacaggagagcgcacgagggagcttccagggggaaacgcctggtatctttatagtcctgtcgggtttcgccacctc
tgacttgagcgtcgatttttgtgatgctcgtcaggggggcggagcctatggaaaaacgccagcaacgcggcctttttacggttcctggccttttgctggccttttgctcac
atgt
